# Revealing the Biological Effect of the *N*‑glycosylation of High-Mobility Group Box 1 (HMGB1) Facilitated by Chemical Protein Synthesis

**DOI:** 10.1021/jacs.6c03294

**Published:** 2026-06-25

**Authors:** Zhixiang Zhong, Haiyan Zhou, Zirong Huang, Yaoyue Zhang, Jiaqi Wang, Han Liu, Xuechen Li

**Affiliations:** a Department of Chemistry, State Key Laboratory of Synthetic Chemistry, 25809 The University of Hong Kong, Pokfulam Road, Hong Kong SAR 999077, P. R. China; b Chemistry and Chemical Engineering Guangdong Laboratory, Shantou, Guangdong 515063, P. R. China; c Shanghai-Hong Kong Joint Laboratory in Chemical Synthesis, Shanghai Institute of Organic Chemistry, University of Chinese Academy of Sciences, Chinese Academy of Sciences, 345 Lingling Road, Shanghai 200032, P. R. China

## Abstract

*N*-glycosylation is one of the most ubiquitous protein post-translational modifications with both high structural diversity and critical functions in broad molecular biological processes. The effect of *N*-glycans on high-mobility group box 1 (HMGB1) has not been clearly elucidated. Chemical protein synthesis, in combination with enzymatic transglycosylation, provides a robust approach to obtain homogeneous *N*-glycoproteins with well-defined glycan structures on designated sites, therefore allowing the investigation of the biological effects of *N*-glycosylation on site- and structure-specific levels. In this work, the synthetic route toward full-length fully reduced HMBG1 was established, which was adopted to the syntheses of *N*-glycosylated HMBG1 variants bearing sialylated biantennary *N*-glycans. The synthetic *N*-glycoproteins were employed to investigate the effects of *N*-glycosylation on the interactions of HMGB1 with receptors RAGE, as well as the HMGB1-stimulated cell migration and CXCL12 secretion. The potential effect of *N*-glycans on the HMGB1/RAGE interaction was investigated via molecular dynamics simulations.

## Graphical abstract

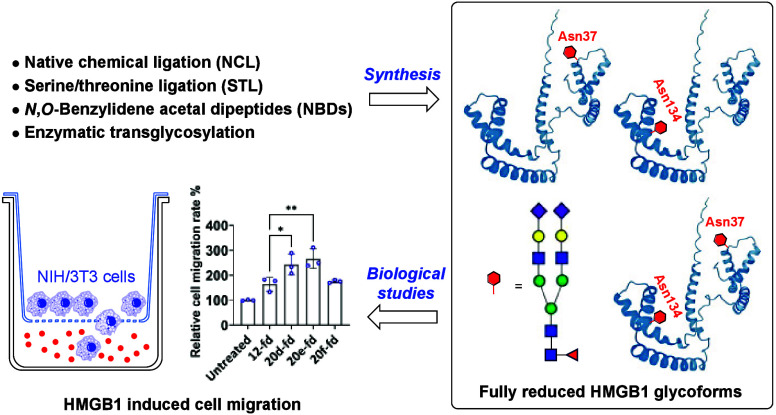

## Introduction

High-mobility group box 1 (HMGB1, formerly known as HMG-1 and amphoterin) protein was discovered in 1973 as a nonhistone nuclear protein playing important roles in DNA packing in chromosomes and named based on their high-electrophoresis mobility on polyacrylamide gels.^1^ HMGB1 is highly conserved during evolution, with more than 95% sequence shared in mammals and 99% homology between rodent and human.^2^ Structurally, the 215-amino-acid sequence of HMGB1 is composed of three domains arranged in an L-shaped fold (Figure 1a). Two positively charged domains A-box (residues 9–79) and B-box (residues 95–163) dominate the DNA binding, while the negatively charged C-terminal acidic tail (residues 186–215) serves as an autoinhibitory factor.^3^ Unlike other HMG proteins mainly residing in the cell nucleus, HMGB1 also functions in the cytosol to promote autophagy^4^ and serves as a danger-associated molecular pattern extracellularly.^5^ The pivotal roles of HMGB1 in pathological processes make it an appealing target for the development of new therapeutics.^6^

**Figure 1:**
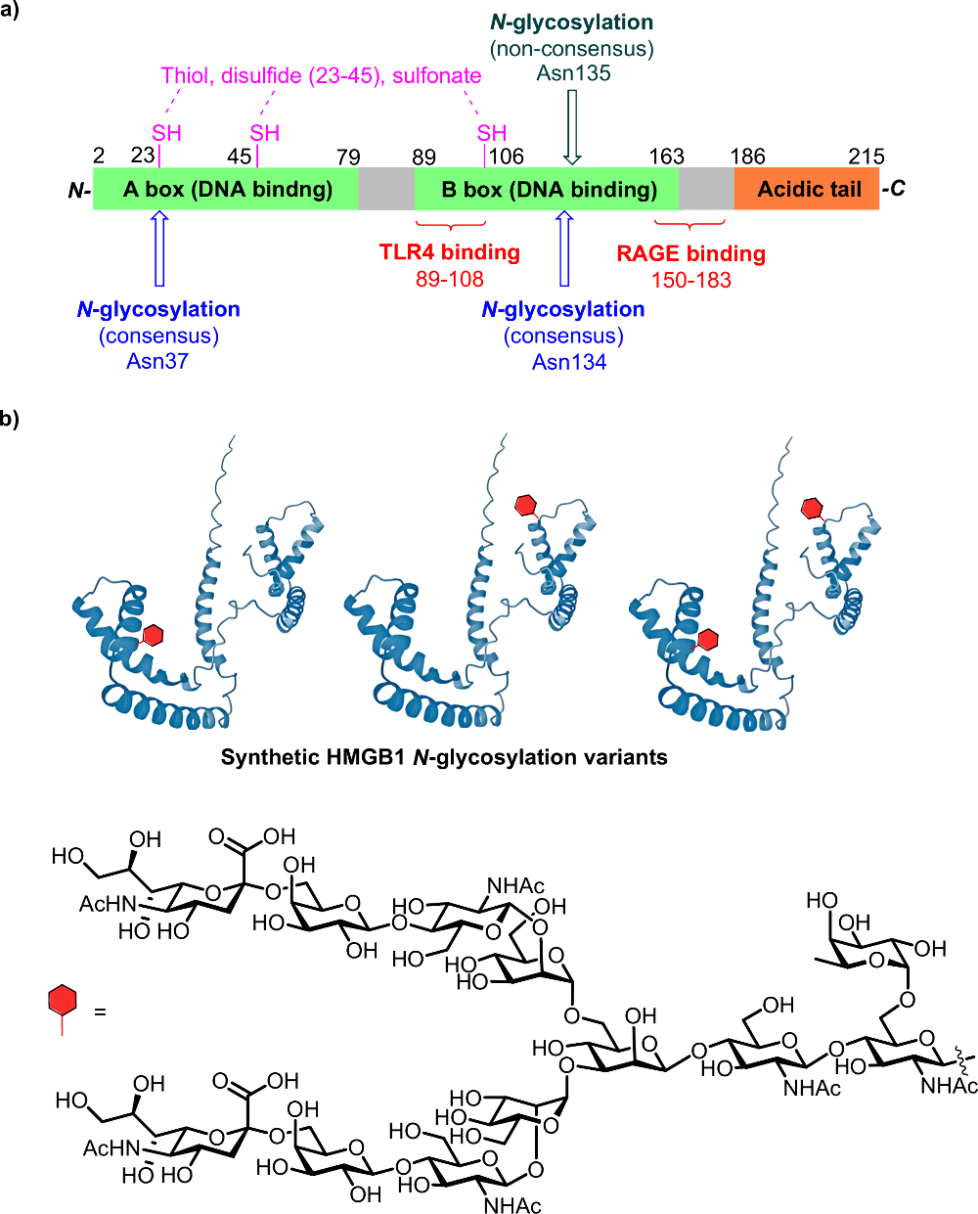
Structure of HMGB1 and *N*-glycosylated variants achieved by chemical synthesis. (a) Functional domains and *N*-glycosylation sites of HMGB1. (b) HMGB1 variants bearing sialylated biantennary complex-type *N*-glycans synthesized in this work.

HMGB1 undergoes extensive redox and nonredox post-translational modifications, which affect its subcellular location, secretion, and functions.^7–9^ The variation of redox status of the three Cys residues (Cys23, 45, and 106) determines the immunological functions of HMGB1.^9^ The fully reduced form of HMGB1 (three feel thiol groups) elicits proinflammatory responses via binding to the receptor for advanced glycation end-products (RAGE)^10^ and forms heterodimer with C-X-C motif chemokine 12 (CXCL12) to promote cell migration via signaling through C-X-C chemokine receptor type 4 (CXCR4).^11^ The disulfide variant of HMGB1 (disulfide linkage between Cys23 and 45) interacts with toll-like receptor 4 (TLR4) in an MD2-dependent manner to stimulate inflammatory cytokine release, where the free thiol on Cys106 is also essential.^12,13^ Unlike the former two redox variants, the sulfonyl HMGB1 (three thiols are oxidized to sulfonates) serves as an anti-inflammatory signaling molecule and recruits immunocompetent cells by signaling via RAGE.^14^ HMGB1 also undergoes nonredox post-translational modifications, including acetylation (Lys), phosphorylation (Ser/Thr), methylation (Lys), *O*-GlcNAcylation (Ser/Thr), and *N*-glycosylation (Asn), within which the *N*-glycosylation shows the highest structural diversity and complexity.^7,8^

As the most ubiquitous PTM of proteins, *N*-glycosylation participates in almost all biological processes, ranging from homeostasis maintenance and life reproduction to interaction with the environment.^15–17^ Initiated in endoplasmic reticulum (ER) and maturated in Golgi apparatus, *N*-glycosylation generates *N*-glycans in a nonlinear and nontemplated manner on ∼75% human proteins.^18^ *N*-glycans assist the folding process during the translation process and participate in the glycoprotein quality control via calnexin/calreticulin cycle.^19,20^ The large-sized highly hydrated *N*-glycans affect the protein conformation and dynamics^21^ and suppress the aggregation when located near the N-termini of aggregation-prone regions (APRs).^22^ *N*-glycans contribute to the stability and pharmacokinetic properties of therapeutic proteins^23^ and serve as handles for engineering and payload incorporation.^24^ Aberrant *N*-glycosylation patterns have been correlated to diseases like brain disorders,^25^ cardiovascular diseases,^26^ autoimmune disease,^27^ and cancers.^28–30^ Revealing the molecular mechanism behind those correlations is highly important for developing sensitive diagnostic and precise therapeutic agents, which is challenging due to the insufficient accessibility of homogeneous *N*-glycoproteins. Along with the maturation of chemical protein synthesis in recent years^31,32^ and improved availability of complex *N*-glycans via chemical^33–39^ and chemoenzymatic^40–47^ approaches, chemically synthesized homogeneous *N*-glycoproteins have become appealing probes to reveal the effect of *N*-glycoforms on biofunctions. Since 2012, *N*-glycoproteins erythropoietin (EPO),^48–54^ interferon-β (IFN-β),^52,55^ interleukin 6,^56–58^ saposin D,^59^ interleukin 3,^60^ chemokine (C–C motif) ligand 1,^60^ interleukin 8,^52^ SARS-CoV-2 spike receptor binding domain (RBD),^61–63^ interleukin 17A,^64^ herpes simplex virus-1 glycoprotein D ectodomain,^65^ receptor for advanced glycation end products (RAGE) V domain,^66^ programmed death 1 (PD-1) IgV domain,^67^ and tau protein^68^ have been chemically synthesized to investigate the effect of *N*-glycans on their stability,^55,57^ binding affinity,^54,59,61–67^ folding quality control,^52^ immunogenicity,^62,65^ and bioactivity.^50,51,53–60,64,66,68^ Those successes encourage scientists to decipher the buried functional code of *N*-glycosylation on more proteins with biological and pathological importance.

The *N*-glycosylation of HMGB1 was first discovered in the 1980s,^69,70^ but the details were not identified until the past decade. In 2016, Kim et al. reported the glycoproteomic and glycomic studies of HMGB1 to identify two consensus (Asn37 and Asn134) and one nonconsensus (Asn135) *N*-glycosylation sites as well as the *N*-glycan structures (Figure 1a).^71^ Though HMGB1 does not possess an N-terminal signal peptide directing it to the ER where *N*-glycan installation happens, experimental evidence shows that HMGB1 still can reach ER possibly through a signal peptide-independent translocation pathway and be *N*-glycosylated.^71^ Treating cells with tunicamycin that inhibits the ER-located enzyme *N*-acetylglucosamine-1-phosphate transferase blocked the *N*-glycosylation of HMGB1. Meanwhile, the identification of sialic acid on the biantennary complex-type *N*-glycan of HMGB1 indicates the *N*-glycan maturation in Golgi apparatus where the sialyltransferase resides.^72^ In terms of function, through Asn-to-Gln mutation, *N*-glycan removal led to increased DNA binding, decreased CRM1 binding, and enhanced nuclear localization. Later, the potential effect of *N*-glycan on the binding of HMGB1 with glycyrrhizin (small-molecule inhibitor) was investigated by molecular simulation.^73^ Until today, HMGB1-bearing acetylation^74^ and *O*-GlcNAcylation^75^ PTMs have been achieved via semisynthesis to reveal their functions, where the synthetic peptide fragments bearing PTMs were joint chemically with unmodified expressed fragments. No total chemical synthesis of HMGB1 and its *N*-glycosylated forms has been reported. Herein, we report our first total chemical synthesis of fully reduced HMGB1 and its *N*-glycosylated variants (Figure 1b) and the effects of *N*-glycosylation on the HMGB1/RAGE interaction, HMGB1-stimulated CXCL12 secretion, and cell migration.

## Results and Discussion

### Chemical Synthesis of Full-Length HMGB1

To maximize the synthetic efficiency for this 200+ amino acids protein, we designed a convergent approach by separating the whole sequence into N-terminal half and C-terminal half with comparable lengths (Figure 2a). Cys106 locating in the middle of the whole sequence makes it an ideal site for final native chemical ligation (NCL),^76,77^ which has been validated in Pratt’s semisynthesis of HMGB1.^75^ The N-terminal half (2–105) would be synthesized through two N-to-C sequential serine/threonine ligations (STL)^78–80^ as a peptide MPAA ester, while the C-terminal half (106–215) would be synthesized through NCL followed by STL in C-to-N manner. As illustrated in Figure 2b, all six segments **1–6** were prepared via standard Fmoc-based solid-phase peptide synthesis (SPPS) joint with C-terminal manipulations when required. An *N*,*O*-benzylidene acetal dipeptide (NBD) building block^81^ was introduced to the Gln21-Thr22 site to improve the SPPS performance of segment **1**. Peptide salicylaldehyde SAL^on^ esters **1** and **4** were synthesized in 28 and 33% yields via “N+1” strategy following the reported procedures, while **2** was kept in the semicarbazone-based SAL^off^ form with 17% yield.^79^ Peptide hydrazide **5** was prepared directly from NH_2_NH_2_ pretreated 2-chlorotrityl resin. Peptide MPAA thioester **3** was generated from the corresponding hydrazide through NaNO_2_-mediated hydrazide activation and thiolysis of peptidyl azide following Liu’s protocol.^82,83^ The N-terminal Cys171 located in peptide **6** was artificially inserted to facilitate NCL, which could be reduced back to Ala via our add-and-done desulfurization.^84,85^

**Figure 2:**
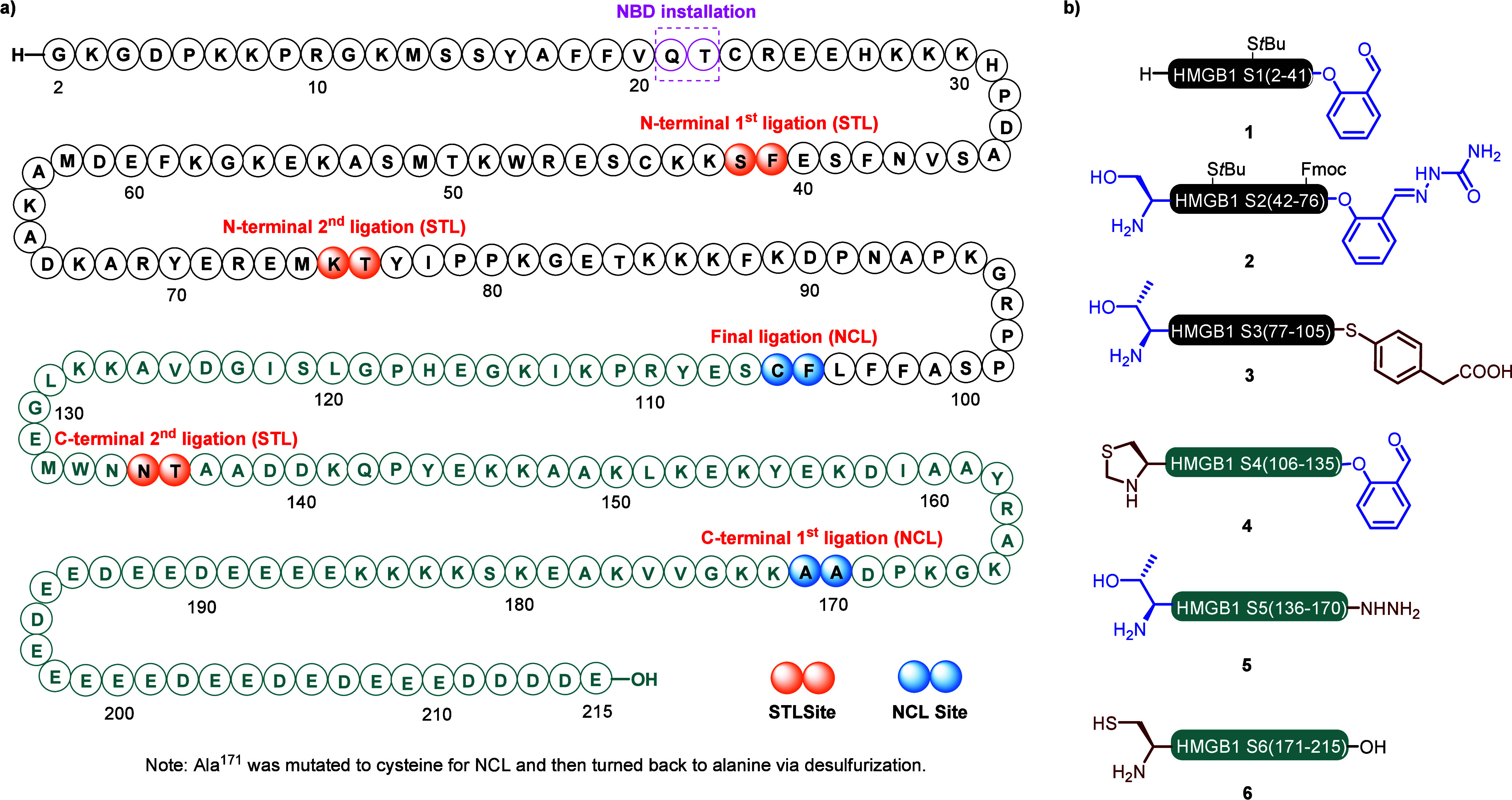
Retrosynthetic analysis of HMGB1. (a) Planned ligation sites for HMGB1 synthesis. (b) Structures of peptide segments.

The synthesis started from the assembly of N-terminal half (2–105) as illustrated in Figure 3a. Peptide SAL^on^ ester **1** (1.4 equiv) and SAL^off^ ester **2** (1 equiv) were subjected to STL in 2,4,6-collidine/HOAc buffer (1:6 *mol*/*mol*, 10 mM) for 13 h. The *N*,*O*-benzylidene acetal intermediate (see Supporting Information for structure details) was treated with TFA/H_2_O/acetylacetone 95/2.5/2.5 *v*/*v*/*v* for acidolysis and SAL ester switch-on,^86^ affording peptide SAL^on^ ester **7** in 25% yield. In the next step, **7** (1 equiv) was joined with peptide MPAA thioester **3** (1.3 equiv) in pyridine/HOAc (1:9, *mol*/*mol*, 10 mM) via threonine ligation. To facilitate the separation of the ligation product from unreacted segment **3**, Fmoc-Gly-SAL^on^ **8** was added to transform the excess **3** into more hydrophobic species. After acidolysis in TFA/H_2_O 95/5 *v*/*v*, the N-terminal half (2–105)-derived peptide MPAA ester **9** was isolated in 29% yield. For the C-terminal half (106–215), peptide hydrazide **5** (1 equiv) was transformed into MPAA ester following Liu’s protocols,^82,83^ followed by the NCL with peptide segment **6** (1.1 equiv) for 12 h. The ligation product was purified by preparative reversed-phase HPLC. After transforming the Cys171 back to Ala171 via NaBEt_4_-mediated ADD desulfurization,^84,85^ peptide **10** was obtained in 30% yield over 3 steps. Subsequently, **10** (1 equiv) was subjected to threonine ligation with peptide SAL^on^ ester **4** (1.5 equiv) in 2,4,6-collidine/HOAc buffer (1:3, *mol*/*mol*, 5 mM) with addition of 30% DMSO to improve the solubility. After 12 h of ligation and acidolysis in TFA/H_2_O/Me_2_S 95/2.5/2.5 *v*/*v*/*v*, the crude peptide precipitated out by cold ether was treated with methoxyamine hydrochloride at pH 4 in the presence of 6 M guanidine to remove Thz protection, and the C-terminal half (106–215) **11** was isolated in 23% yield (over 3 steps). Finally, the N-terminal half thioester **9** (1 equiv) and C-terminal half **11** (1 equiv) were subjected to final NCL (phosphate buffer, 6 M guanidine, pH = 7, 2.5 mM) with MPAA addition (50 equiv) for 12 h. After ligation, the *tert*-butylthio (S*t*Bu) protection on cysteine residues as well as the disulfide linkage between Cys106 and MPAA were cleaved by TCEP, and the Fmoc protection on Lys76 was removed by 20% piperidine. The full-length HMGB1 (2–215) peptide **12** was isolated in 31% yield over three steps. The UPLC trace (Figure 3b) and high-resolution ESI-MS (Figure 3c) indicated high purity and correctness of **12**. The unmodified synthetic HMGB1 peptide **12** was folded following the previously reported method to yield **12-fd**,^74^ and the existence of free thiol groups in the folded protein was confirmed by comparing the high-resolution ESI-MS of **12-fd** with the TCEP-treated sample (Figures S63 and S64). For comparison, we expressed HMGB1 protein with the same sequence in the *E. coli* system.^74^ The folded synthetic HMGB1 **12-fd** showed the same circular dichroism (CD) spectrum as the expressed one (Figure 3d). Furthermore, we assessed the binding affinities between fully reduced HMGB1 and RAGE via microscale thermophoresis (MST) measurement. The synthetic HMGB1 **12-fd** bound to HEK 293T-derived hRAGE (MedChemExpress) with an affinity (K_D_ = 44.9 ± 30.4 nM) similar to that for *E. coli* expressed HMGB1 (K_D_ = 40.3 ± 28.5 nM) (Figure 3e). These data further confirmed the formation of the correct tertiary structure of synthetic protein and suggested that our synthetic route was ready for constructing HMGB1 *N*-glycosylation variants for functional studies.

**Figure 3:**
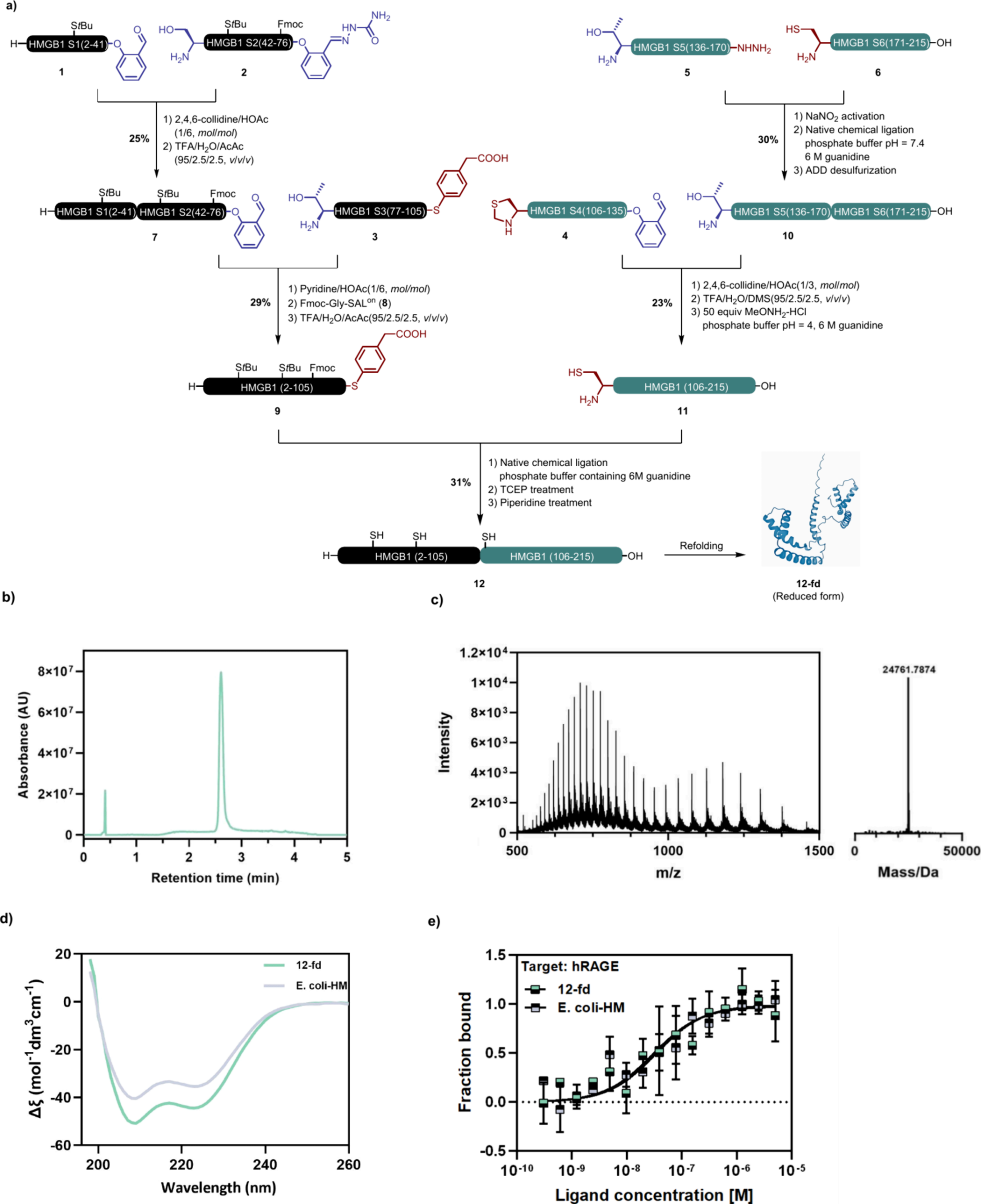
Total chemical synthesis of full-length fully reduced HMGB1 protein. (a) Synthetic route for unmodified HMGB1. (b) UPLC trace of synthetic full-length HMGB1. (c) High-resolution ESI-MS spectrum of synthetic HMGB1 and deconvoluted HR-ESI-MS result. (d) CD spectra of synthetic and *E. coli* expressed HMGB1. (e) Binding affinities of synthetic and *E. coli* expressed HMGB1 with hRAGE measured by MST. The curve points were shown with mean ± SD of 3–4 independent experiments.

### Chemical and Chemoenzymatic Synthesis of *N*-glycosylated HMGB1 Variants

To illustrate the function of glycosylated HMGB1 variants, we selected two consensus *N*-glycosylation sites Asn37 and Asn134 on HMGB1 for *N*-glycan installation. To facilitate the installation of sialylated biantennary glycan, we leveraged the chemoenzymatic approach developed by Wang et al., which involves the introduction of Fuc-α1,6-GlcNAc disaccharide to the designated *N*-glycosylation sites via SPPS followed by enzymatic transglycosylation for glycan extension.^87,88^ The building block Fmoc-Asn­(Fuc-α1,6-GlcNAc)–OH **13** was prepared following the published route^89^ and was inserted to glycopeptide SAL ester segments **14** and **15** in the same way as the nonglycosylated ones (Scheme 1a). Thereafter, the *N*-glycosylated N-terminal half **17** and C-terminal half **19** of HMGB1 were synthesized by adopting the same ligation sequences as described above (Scheme 1b,c) with comparable efficiency. The *O*-acetyl groups on the disaccharide were removed by hydrazine treatment to yield **18**, while kept intact in case of **17** due to the incompatibility of acetyl removal conditions with SAL ester and Fmoc.

**Scheme 1:**
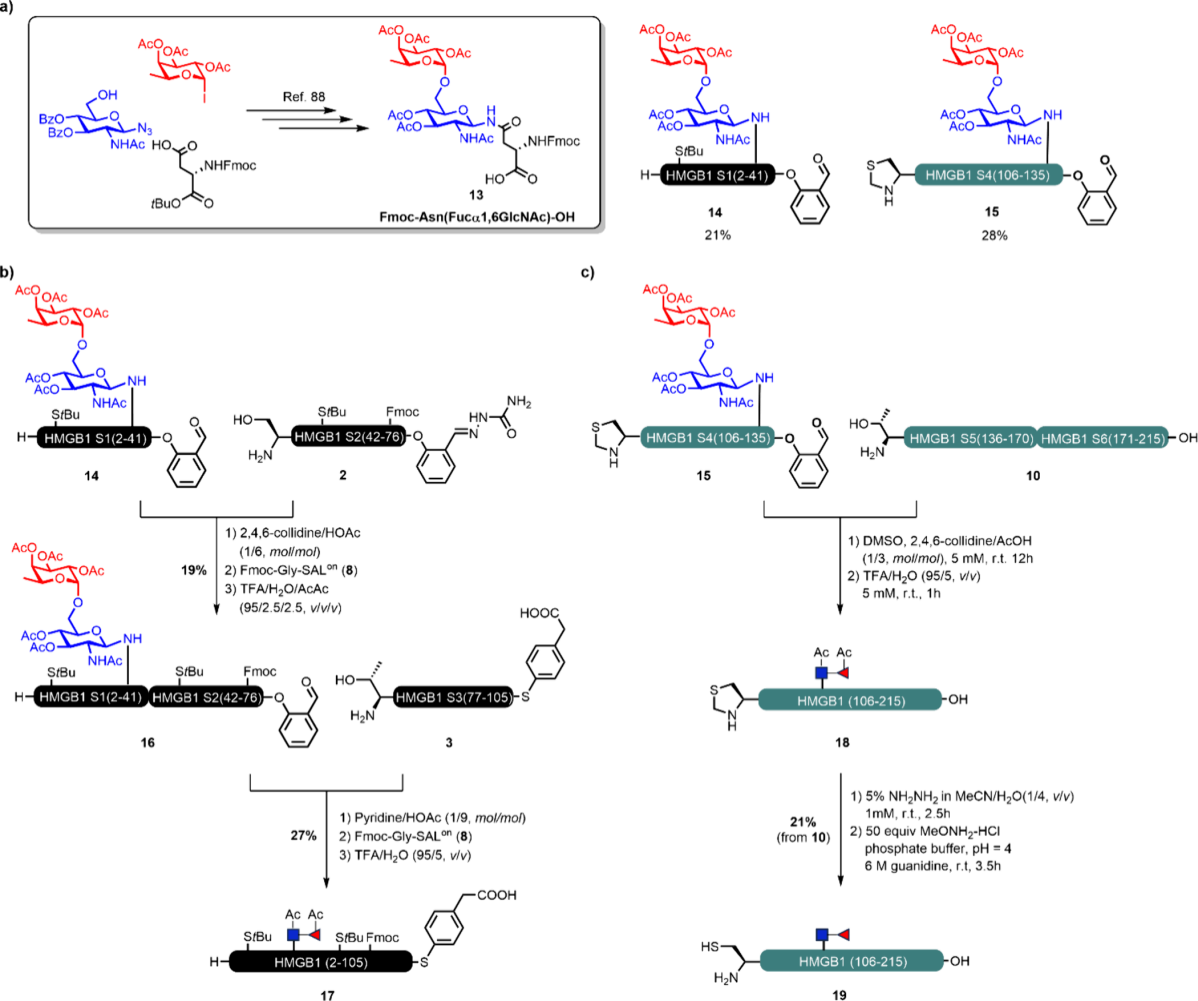
Synthesis of *N*-Glycosylated Peptide Segments of HMGB1^a^

a (a) Synthesis of disaccharide-bearing Asn building block and glycopeptides. (b) Synthesis of *N*-glycosylated N-terminal half of HMGB1. (c) Synthesis of *N*-glycosylated C-terminal half of HMGB1.

Next, we incorporated both glycosylated and nonglycosylated segments to assemble *N*-glycosylated HMGB1 variants. As illustrated in Figure 4a, three HMGB1 variants **20a**–**c** bearing single and double *N*-glycan modifications were easily generated via ligating corresponding N- and C-terminal half segments in the final NCL step (**17** + **11** for **20a**, **9** + **19** for **20b**, **17** + **19** for **20c**) followed by one-pot sequential removal of S*t*Bu, *O*-acetyl, and Fmoc groups. The HMGB1 *N*-glycosylated variants **20a** to **20c** were isolated in 28–37% yields, comparable to the nonglycosylated HMGB1 **12**. The high purity of the synthetic glycoproteins was demonstrated by UPLC traces and HR-ESI-MS analyses (Figures 4b–5d).

**Figure 4:**
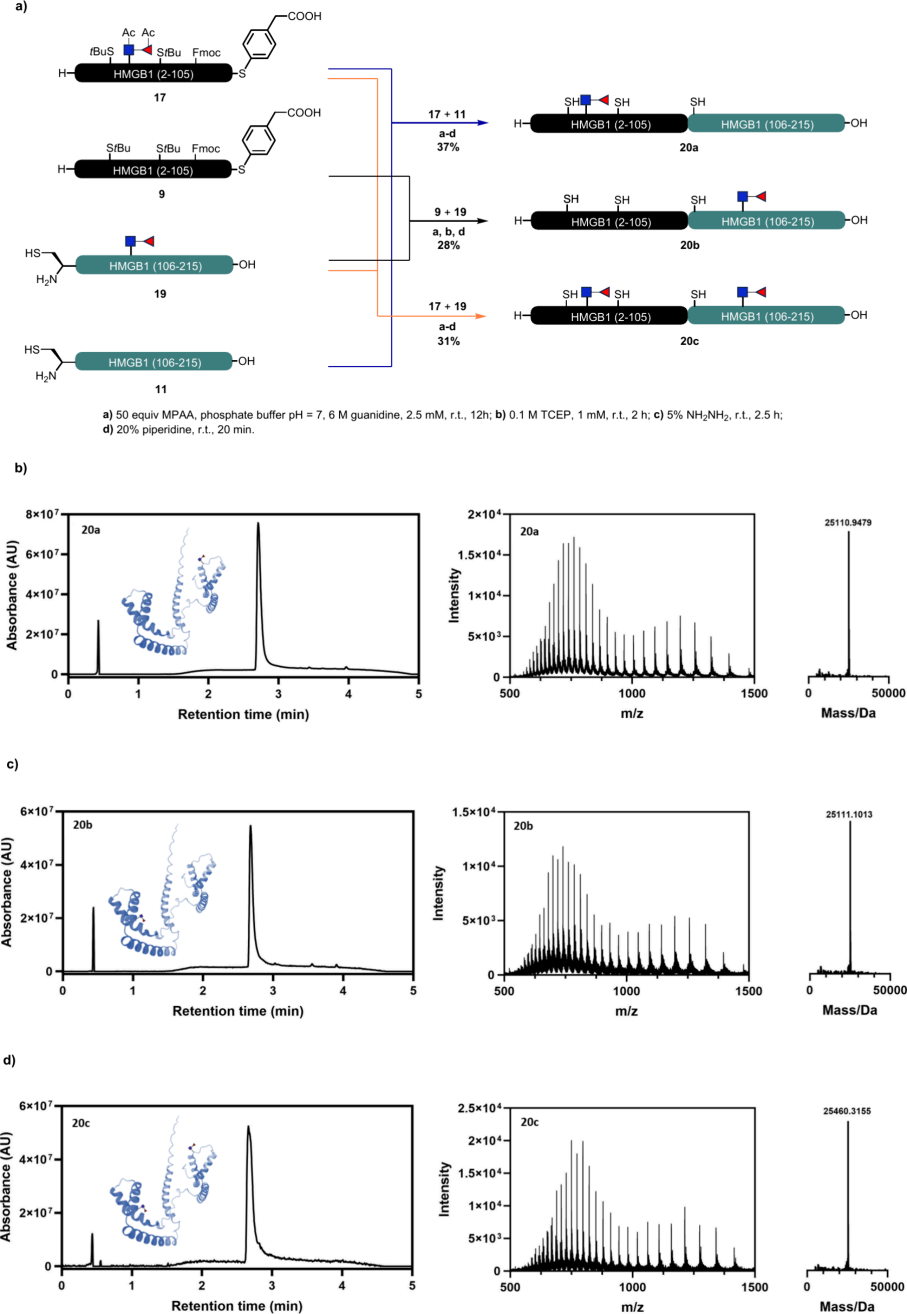
Synthetic scheme and characterization of HMGB1 variants bearing disaccharide. (a) Construction procedures for HMGB1 variants bearing disaccharide *N*-glycans (**20a** to **c**). (b–d) UPLC analysis and high-resolution ESI mass spectrometry analysis of **20a** to **20c.**.

**Figure 5:**
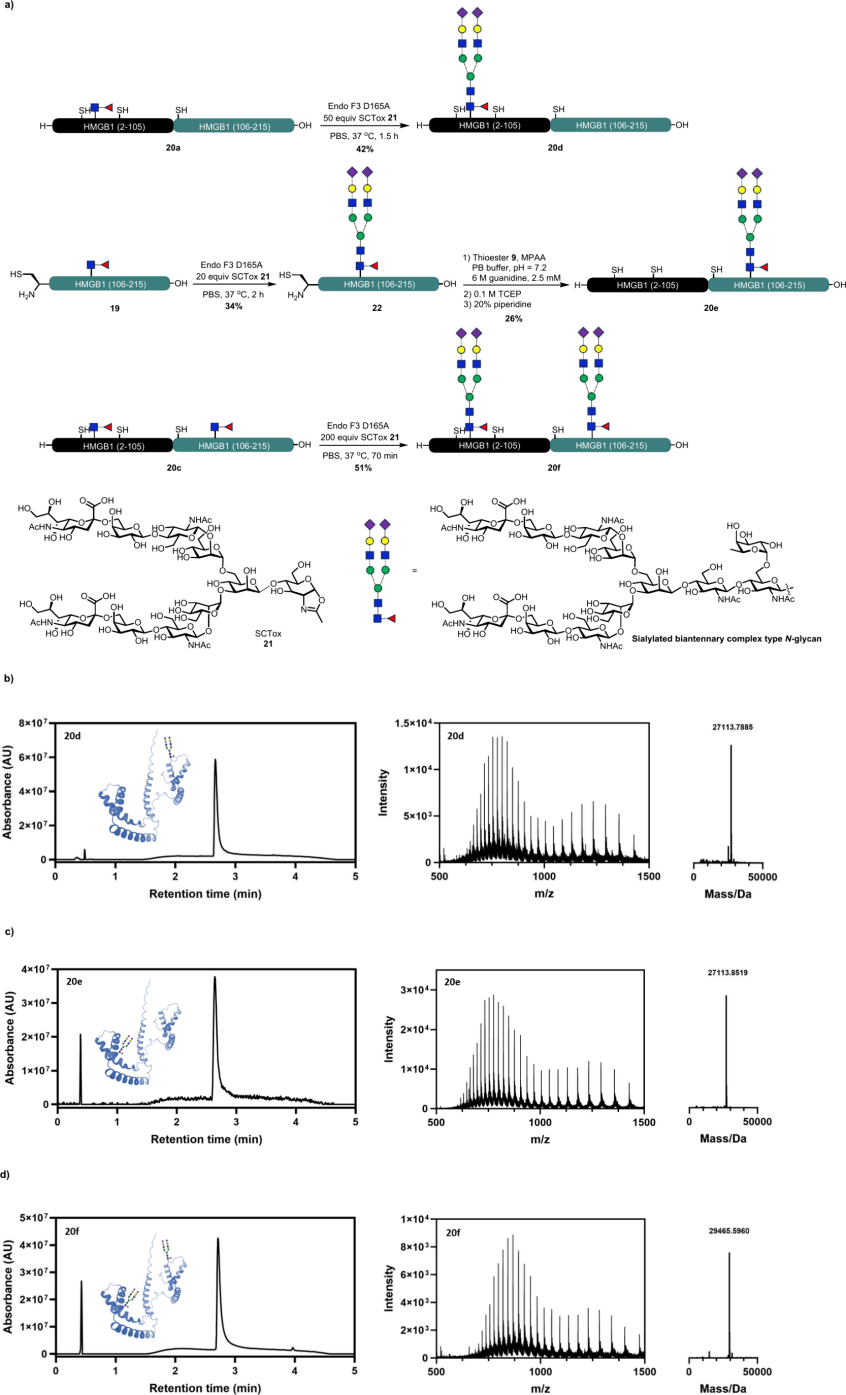
Synthesis of fully reduced HMGB1 variants bearing sialylated biantennary complex-type *N*-glycans via enzymatic transglycosylation. (a) Transglycosylation on glycopeptide and full-length HMGB1. (b–d) UPLC analysis and high-resolution ESI mass spectrometry analysis of **20d** to **20f.**.

With the disaccharide modified HMGB1 variants in hand, we pursued the ones bearing single- and double-sialylated biantennary complex-type *N*-glycans. Endo F3 D165A mutant developed by Wang et al.^90^ was applied to facilitate the transglycosylation of sialylated biantennary complex-type *N*-glycan decasaccharide-derived oxazoline (SCTox) **21** ^91^ onto *C*4-OH of the *O*6-α-fucosylated GlcNAc to form β-glycosidic linkage.^88^ As shown in Figure 5a, glycopeptide **19** was treated with 20 equiv of SCTox in neutral PBS buffer for 2 h to yield glycopeptide **22** in 34% yield. Elongation of the reaction time led to decreased yield due to the inversed glycosidic bond cleavage process catalyzed by the same enzyme. Peptide **22** was then ligated with nonglycosylated N-terminal half thioester **9** under NCL conditions, followed by S*t*Bu and Fmoc removal. The HMGB1 variant **20e** bearing undecasaccharide on Asn134 was isolated in 26% yield. To avoid the decrease of the final ligation efficiency caused by large *N*-glycan, we tested the transglycosylation to full-length *N*-glycosylated HMGB1 variants **20a** and **20c**. To our delight, treating **20a** with 50 equiv of SCTox for 1.5 h afforded glycoprotein **20d** in 42% yield. When **20c** containing two *N*-glycosylation sites was used as substrate, glycoprotein product **20f** was obtained in 51% yield by using 200 equiv of SCTox. The synthetic glycoproteins **20d** to **20f** were characterized by UPLC and high-resolution ESI mass spectrometry to demonstrate purity and correctness. Finally, after folding under reported conditions,^74^ all the six *N*-glycosylated HMGB1 variants **20a-fd** to **20f-fd** showed CD spectra well aligned with *E. coli* expressed HMGB1 without *N*-glycan, indicating the formation of correct 3D structures (Figure 6). The three *N*-glycosylated variants bearing undecasaccharide glycans (**20d-fd** to **20f-fd**) showed improved conformational stability compared to the *E. coli* expressed HMGB1, as indicated by the thermal denaturation assay (see Figure S65).

**Figure 6:**
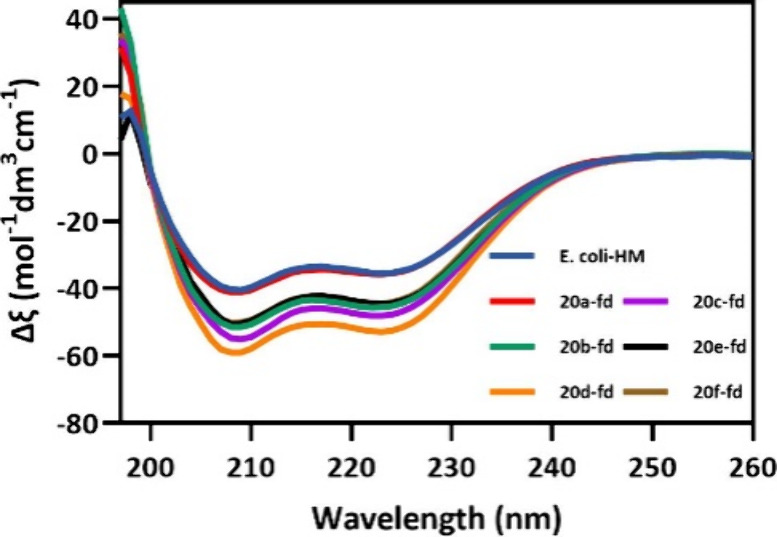
Comparison of CD spectra of refolded synthetic *N*-glycosylated fully reduced HMGB1 variants with expressed nonglycosylated HMGB1.

### Biological Studies

With the *N*-glycosylated HMGB1 variants **20d-fd** to **20f-fd** bearing native sialylated biantennary complex-type *N*-glycans as well as the nonglycosylated one in hand, we investigated their affinities to receptor RAGE. The *K_D_* values were measured by microscale thermophoresis (MST) using fluorophore-labeled human RAGE (hRAGE). As summarized in Figure 7a, the *K_D_* value of binding among glycosylated HMGB1 variants with hRAGE ranged from 21.1 to 35.6 nM, which indicates the high binding ability of *N*-glycosylated HMGB1 variants. All three HMGB1 variants showed potentially small improvement (not statistically significant due to the fluctuation of MST data) on binding affinity with hRAGE (Figure 7b). HMGB1 is reported to bind to RAGE via two interacting motifs: residues 23 to 45 in the A-box^92^ and residues 150 to 183 in the B-box.^93,94^ The *N*-glycan on Asn34 located in the former motif may directly participate in the interaction with the V–C1 region of RAGE, while the one on Asn137 may contribute indirectly by stabilizing the binding conformation of HMGB1. The molecular dynamics simulation was also conducted to further elucidate the contribution of *N*-glycan to the receptor binding interaction (*vide infra*).

**Figure 7:**
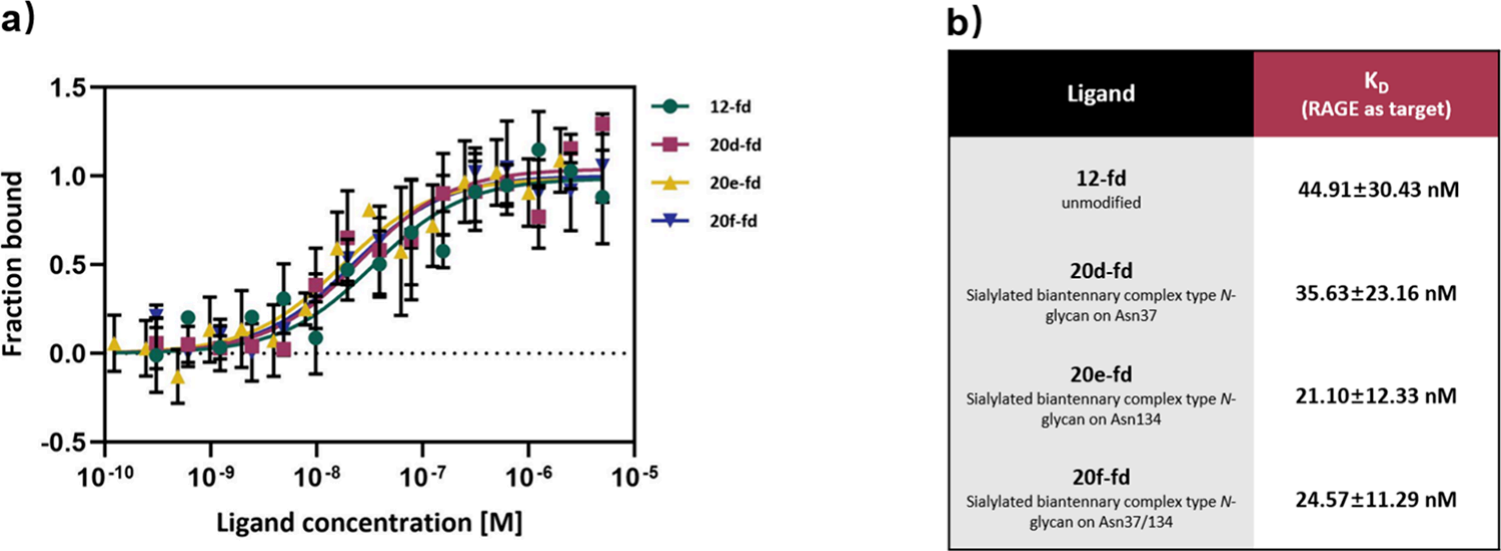
Binding affinity measurements among synthetic HMGB1 variants bearing sialylated biantennary complex-type *N*-glycan and RAGE. (a) MST binding affinity curves of **20d-fd** to **20f-fd** and **12-fd** to HEK 293T cell-derived hRAGE (MedChemExpress). The curve points were shown with mean ± SD of 3–4 independent experiments. (b) Summarized *K_D_* values based on the MST binding affinity measurements. The error represents the 95% confidence intervals of the curve fitting.

Next, we investigated the effects of the *N*-glycosylated HMGB1 variants on cell migration. The fully reduced HMGB1 is responsible for cell migration related with damage repair and tissue regeneration.^95,96^ HMGB1 is reported to stimulate the migration of mouse embryonic fibroblasts (MEFs), human monocytes, and Schwann cells.^11,96,97^ As immortalized MEFs, NIH/3T3 cells undergo promoted cell migration and scratch closure when treated with HMGB1.^96^ To verify the effects of *N*-glycosylation of HMGB1 on cell chemotaxis, transwell migration assay was used for assessment of the cell migration of NIH/3T3 cells. The cells were seeded in 24-well transwell inserts with serum-free medium, and synthetic unmodified HMGB1 **12-fd** and its *N*-glycosylated variants **20d-fd** to **20f-fd** were placed in the lower chamber premixed with culture medium. In this assay (Figure 8), we observed an increased relative cell migration rate in the groups treated with HMGB1 proteins compared to the untreated group, which was consistent with the reported migration-promoting activity of HMGB1.^11,96^ Besides, *N*-glycosylation can further increase the chemoattractive effect of HMGB1 in NIH/3T3 cells. Among the three *N*-glycosylated HMGB1 variants, **20d-fd** and **20e-fd** showed a significant enhancement of the relative cell migration rate, while the effect of **20f-fd** was marginal.

**Figure 8:**
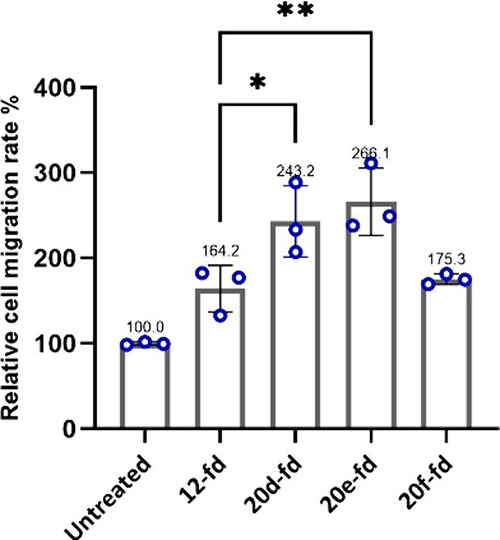
*N*-glycosylated HMGB1 variants promoted migration of NIH/3T3 cells. NIH/3T3 cells were seeded in 24-well transwell inserts (CoStar, polycarbonate membrane, 8 μm pore diameter, USA) with serum-free medium. The lower chambers were placed with normal culture medium with or without HMGB1. The cells were incubated for 9 h with 5% CO_2_ in 37 °C. After incubation, inserts were removed, fixed and stained with 0.1% crystal violet solution. 33% acetic acid was used to elute out the staining in the cells. The absorbance at 590 nm was read with a microplate reader, which is positively correlated with the number of migrated cells. The data was presented by the mean ± SEM of three independent experiments. Statistical significance was measured by one-way ANOVA using GraphPad Prism 10 software. (*P < 0.05; **P < 0.01).

HMGB1-induced cell migration is RAGE and C-X-C chemokine receptor type 4 (CXCR4) dependent, where CXCL12 serves as a key intermediary molecule.^98^ CXCL12, also known as stromal cell-derived factor 1 (SDF-1), is a small-molecule cytokine that plays an important role in various physiological processes such as embryogenesis, hematopoiesis, and angiogenesis.^99^ CXCL12 mediates cell chemotaxis and adhesion by signaling through its receptor CXCR4.^98^ It is reported that HMGB1 stimulates the CXCL12 production in mouse embryonic fibroblasts in a RAGE-dependent manner,^11,100^ and the HMGB1-CXCL12 heterodimer formation is essential for attracting monocytes to injured tissues via interacting with CXCR4.^11^ To understand the enhancing effect of HMGB1 *N*-glycosylation on induced cell migration, we hypothesized that *N*-glycosylation could increase the capability of HMGB1 on the stimulation of CXCL12 secretion. To verify our hypothesis, we investigated the secretion level of CXCL12 in NIH/3T3 cells stimulated by our synthetic, fully reduced HMGB1 *N*-glycosylation variants. We measured the secreted CXCL12 levels in NIH/3T3 cells after incubation with HMGB1 and its variants via enzyme-linked immunosorbent assay (ELISA) (Figure 9), where *E. coli* **-HM** served as a positive control. An elevated CXCL12 secretion level was observed when treated with *E. coli* **-HM** and the synthetic unmodified HMGB1 (**12-fd**), which well matched with the reported results^11^ and further validated the biological equivalence between chemically synthesized HMGB1 and *E. coli*-expressed one on cell level. HMGB1 variants **20d-fd** and **20e-fd** bearing a single *N*-glycan on Asn37 and Asn134, respectively, showed a significant increase in CXCL12 secretion levels, while **20f-fd** bearing *N*-glycans on both sites showed a moderate increase. This result is consistent with the cell migration assay, verifying our hypothesis that *N*-glycosylation enhances the chemoattractive effect of HMGB1 by increasing its capability of stimulating CXCL12 secretion.

**Figure 9:**
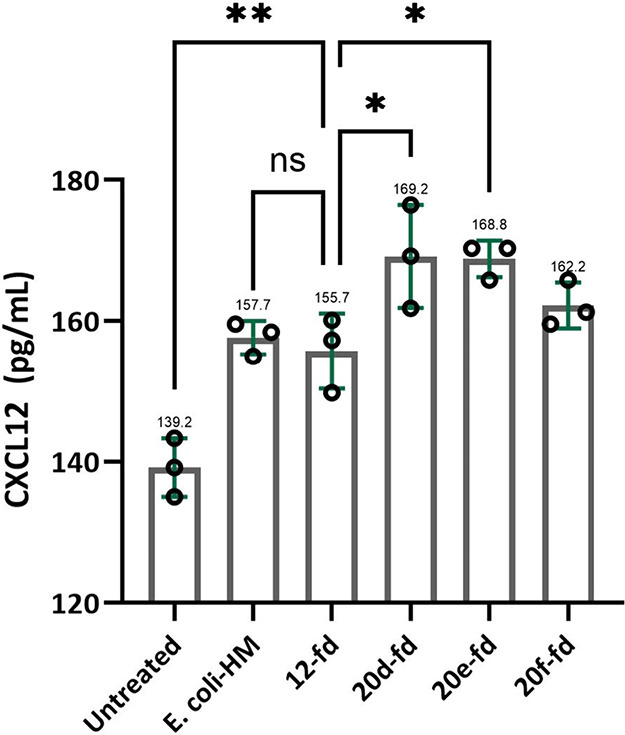
HMGB1 and its variants upregulate CXCL12 secretion levels in NIH/3T3 cells. NIH/3T3 cells were seeded in 96-well plate simultaneously with 180 nM HMGB1 addition. The cells were incubated for 12 h with 5% CO_2_ in 37 °C. CXCL12 levels in cell supernatants were detected by sandwich ELISA following the manufacture’s protocols (R&D systems, USA). The data was presented by mean ± SEM of three independent experiments. Statistical significance was measured by one-way ANOVA using GraphPad Prism 10 software. (ns, not significant; *P < 0.05; **P < 0.01).

### Molecular Dynamics Simulation

To elucidate the molecular mechanism by which HMGB1 *N*-glycosylation modulates receptor recognition, we performed 200 ns all-atom molecular dynamics (MD) simulations on hRAGE in complex with four HMGB1 variants: **12-fd** (nonglycosylated), **20d-fd** (glycan on Asn37), **20e-fd** (glycan on Asn134), and **20f-fd** (glycans on both sites) (Figure 10). The calculation details are provided in the Supporting Information. Root-mean-square deviation (RMSD) analysis revealed that all three *N*-glycosylated HMGB1 variants exhibited more stable trajectories than nonglycosylated **12-fd** throughout the 200 ns simulation (Figure 10a,b). The violin plot distributions showed narrower RMSD ranges and lower median values for the glycosylated variants, indicating that *N*-glycosylation stabilizes the RAGE-HMGB1 complex. This enhanced stability was further confirmed by the root-mean-square fluctuation (RMSF) analysis of atoms (Figure 10c,d), which showed progressively smaller areas under the curve (S values) for glycosylated variants: *S* = 47.21 for **12-fd**, 34.18 for **20d-fd**, 27.50 for **20e-fd**, and 21.32 for **20f-fd**. These results indicate that glycosylation reduces atomic fluctuations around the equilibrium positions, consistent with a more rigid and stable binding interface.

**Figure 10:**
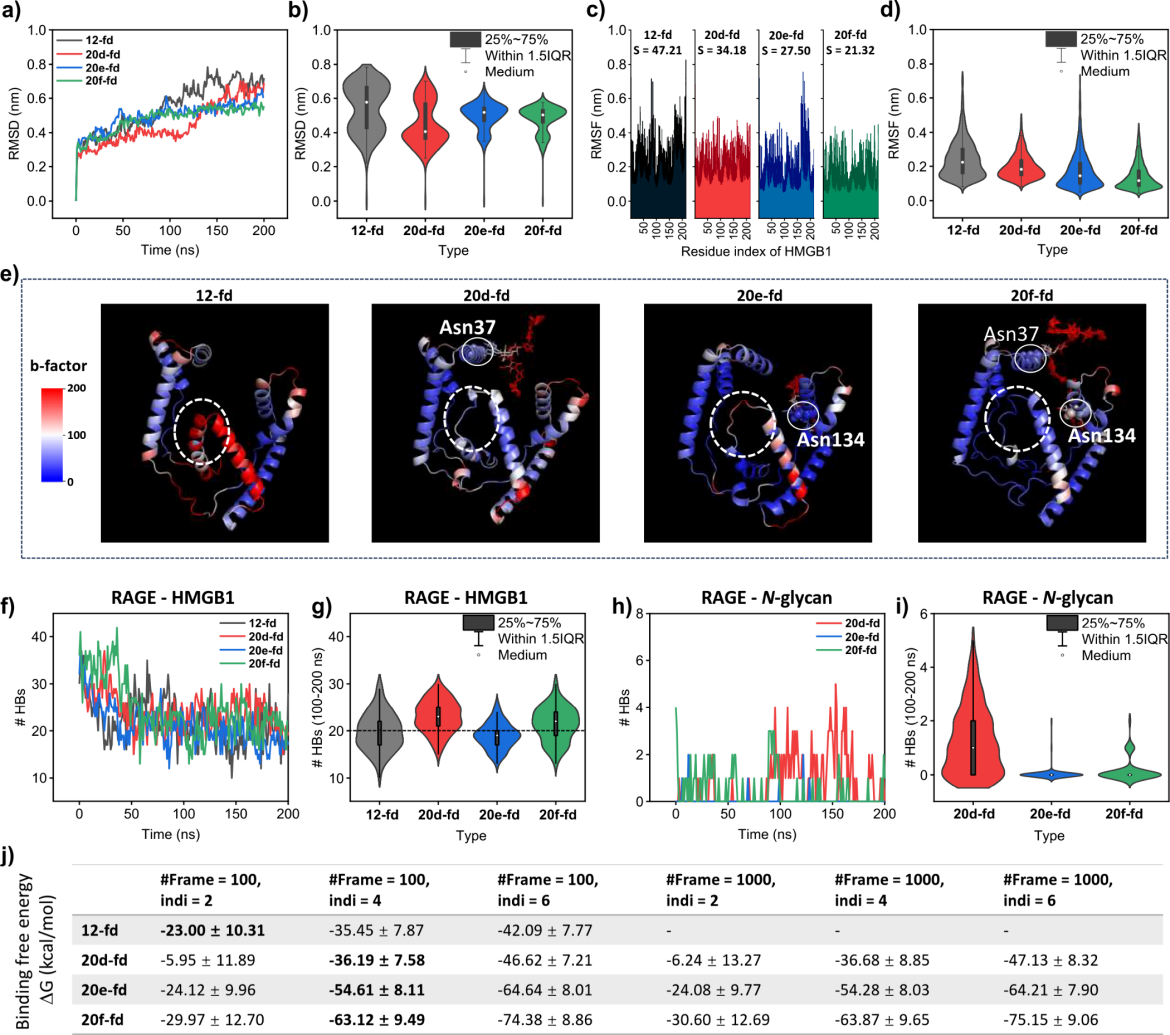
Molecular dynamics simulation of RAGE in complex with HMGB1 and its three glycosylated variants: **12-fd** (nonglycosylated HMGB1), **20d-fd** (glycosylated at Asn37), **20e-fd** (glycosylated at Asn134), and **20f-fd** (glycosylated at both Asn37 and Asn134). (a) RMSD evolution during the 200 ns simulation. (b) Violin distribution of RMSD values from panel (a). IQR stands for interquartile range, which is the range between the first quartile (Q1, 25th percentile) and the third quartile (Q3, 75th percentile). (c) RMSF during 100 to 200 ns. A smaller area under the curve (S) indicates smaller deviation of the atoms from their equilibrium positions. (d) Violin distribution of RMSF values from panel (c). (e) B-factor illustrations of the structures of HMGB1 and its three glycosylated variants. The structures represent averaged positions over 100 to 200 ns. Only the HMGB1 structures extracted from the RAGE–HMGB1 complexes are shown for simplicity. (f) Evolution of the number of hydrogen bonds (HBs) between RAGE and HMGB1 (including glycan) from 0 to 200 ns. (g) Violin distribution of HBs from panel (f) during 100 to 200 ns. (h) Evolution of the number of HBs between RAGE and the *N*-glycan from 0 to 200 ns. (i) Violin distribution of HBs from panel (h) during 100 to 200 ns. (j) Binding-free energy Δ*G* (kcal/mol), tested with different numbers of frames and different values of the parameter indi (i.e., solute dielectric constant, affecting the strength of Coulombic interactions). Indi = 4 is recommended for systems containing glycosylated proteins. Indi = 1 or 2 is recommended for canonical protein systems.

B-factor analysis of time-averaged structures (100–200 ns) revealed intriguing site-specific effects (Figure 10e). Notably, the acidic C-terminal tail region (residues 189–198, EDEEDEEDEE) exhibited distinct conformational behavior depending on glycosylation status. In nonglycosylated **12-fd**, this region maintained an α-helical conformation but showed high B-factors indicative of substantial mobility. In contrast, in all three glycosylated variants, this region lost helical structure but paradoxically displayed lower B-factors, suggesting a transition to a more stable extended conformation. This observation indicates that *N*-glycosylation induces long-range conformational effects that extend beyond the immediate glycosylation sites (Asn37 and Asn134), potentially through altered electrostatic interactions or changes in the overall conformational ensemble of HMGB1.

A key finding from our simulations is that the *N*-glycans are not passive structural elements but actively contribute to receptor binding. Analysis of hydrogen bonds (HBs) between RAGE and HMGB1 (including glycan) showed that glycosylated variants form more total HBs (**20d-fd** and **20f-fd**) or stabler HBs (**20e-fd**) than nonglycosylated **12-fd** (Figure 10f,g). Critically, when we specifically analyzed HBs between RAGE and the *N*-glycan moieties alone (Figure 10h,i), we observed intermittent glycan–RAGE interactions throughout the equilibrated portion of the trajectories (100–200 ns), especially in the case of **20d-fd**. These results support a model in which HMGB1 *N*-glycosylation contributes directly to the RAGE-HMGB1 interface.

To quantitatively assess the energetic impact of *N*-glycosylation, we performed MM/GBSA binding-free energy calculations using different sampling parameters (Figure 10j).^101^ Using the recommended solute dielectric constant for glycosylated proteins (indi = 4, indi = 2 for nonglycosylated HMBG1) and 100 frames from the equilibrated trajectory, the calculated Δ*G* values were −23.00 ± 10.31 kcal/mol for **12-fd**, −36.19 ± 7.58 kcal/mol for **20d-fd**, −54.61 ± 8.11 kcal/mol for **20e-fd**, and −63.12 ± 9.49 kcal/mol for **20f-fd**. This trend was maintained across different sampling conditions, confirming the robustness of the result. The enhancement in binding affinity upon glycosylation, particularly for **20e-fd** and **20f-fd**, is in agreement with our MST measurements.

While the MD simulations and MST data both indicate strong RAGE binding for Asn134-glycosylated variants **20e-fd** and doubly glycosylated **20f-fd**, our cell-based functional assays showed that singly glycosylated **20d-fd** and **20e-fd** were more effective than **20f-fd** in promoting NIH/3T3 cell migration and CXCL12 secretion. This apparent discrepancy provides important mechanistic insight: maximal receptor binding affinity does not necessarily translate to an optimal biological activity. We propose that productive HMGB1-RAGE signaling requires not only strong initial receptor engagement but also appropriate conformational dynamics to facilitate downstream events such as receptor clustering, coreceptor recruitment, or conformational changes required for signal transduction. The doubly glycosylated **20f-fd**, while forming the most stable binary complex, may be overstabilized in a conformation that is suboptimal for these subsequent steps. In contrast, singly glycosylated variants may achieve a favorable balance between enhanced receptor affinity (compared to nonglycosylated HMGB1) and retained conformational flexibility necessary for productive signaling.

In summary, our MD simulations provide atomic-level insights into how HMGB1 *N*-glycosylation modulates RAGE recognition through three synergistic mechanisms: (i) direct glycan-receptor hydrogen bonding, (ii) stabilization of the complex conformation with reduced fluctuations, and (iii) long-range conformational effects on HMGB1 structure. These computational findings not only validate our experimental binding measurements but also help to explain the complex relationship between binding affinity and biological activity observed in our cell-based assays.

## Conclusions

In this work, we finished the first chemical total synthesis of the full-length fully reduced HMGB1 protein via a convergent approach by jointly using serine/threonine ligation and native chemical ligation. This approach with good overall efficiency and modularity was then adopted for the synthesis of three *N*-glycosylated HMGB1 variants bearing sialylated biantennary complex-type N-glycans at Asn37 and/or Asn134. All of the synthetic proteins were well-folded and showed binding affinities with their receptor hRAGE. For HMGB1-hRAGE interaction, *N*-glycosylation improved the binding affinity compared to nonglycosylated HMGB1. We evaluated HMGB1-induced migration of NIH/3T3 cells via a transwell migration assay, where **20d-fd** and **20e-fd** bearing single biantennary *N*-glycan on Asn37 and Asn134, respectively, promoted the migration of NIH/3T3 cells significantly with higher efficacy compared to **12-fd** and **20f-fd**. We further investigated the HMGB1-stimulated CXCL12 secretion and found that *N*-glycosylated HMGB1 variants **20d-fd** and **20e-fd** stimulated a higher CXCL12 secretion level than **12-fd** and **20f-fd**. The consistency between CXCL12 secretion level and cell migration rate indicates that *N*-glycosylation on Asn37 or Asn134 improves HMGB1-promoted cell migration via enhancing the capability of stimulating CXCL12 secretion. To further elucidate the molecular mechanism of the stabilizing effect of *N*-glycan in the HMGB1/RAGE interaction, we conducted MD simulation studies. The introduction of *N*-glycans on single and double sites could enhance the stability of the HMGB1/RAGE complex through reducing conformational fluctuation, direct glycan-receptor hydrogen-bonding interaction, and the long-range conformational effect of HMGB1. Though the *N*-glycan structures in nature are far more complicated than the undecasaccharide we employed here,^71^ our work for the first time revealed the effects of HMGB1 *N*-glycosylation on its interactions with its major receptor RAGE, which is relevant to both its chemoattractant function as chemokine and its proinflammation function as cytokine. Since HMGB1 is rich in post-translational modifications (*N*/*O*-glycosylation, acetylation, methylation, phosphorylation, redox status, etc.),^8^ the synergistic effect of HMGB1 post-translational modifications on pathogenetic processes is under investigation in our lab.

## Supplementary Material



## Notes

Details of peptide and protein synthesis and biological experiments, characterizations of synthetic peptides, computational studies (PDF)
