# Supplementary material for: Revealing the Biological Effect of the N‑glycosylation of High-Mobility Group Box 1 (HMGB1) Facilitated by Chemical Protein Synthesis
Source: J Am Chem Soc. 2026 Jun 25;148(26):27219–33. doi: 10.1021/jacs.6c03294 (PMC13352598; doi:10.1021/jacs.6c03294)
Supplement: Supplementary file 1 [file ja6c03294_si_001.pdf]

Supplementary Information

Revealing the Biological Effect of the *N*-Glycosylation of High Mobility Group Box 1 (HMGB1) Facilitated by Chemical Protein Synthesis

Zhixiang Zhong,[a,b] Haiyan Zhou,[a] Zirong Huang,[a] Yaoyue Zhang,[a] Jiaqi Wang,[a*] Han Liu,[a*] Xuechen Li[a,c*]

[a] Department of Chemistry, State Key Laboratory of Synthetic Chemistry, The University of Hong Kong, Pokfulam Road, Hong Kong SAR, 999077, P. R. China

[b] Chemistry and Chemical Engineering Guangdong Laboratory, Shantou, Guangdong, 515063, P. R. China

[c] Shanghai-Hong Kong Joint Laboratory in Chemical Synthesis, Shanghai Institute of Organic Chemistry, University of Chinese Academy of Sciences, Chinese Academy of Sciences, 345 Lingling Road, Shanghai, 200032, P. R. China

E-mail: benwang@hku.hk; liuhan@hku.hk; xuechenl@hku.hk

I. General Experimental Procedures…………………………………….…... S4

A) Standard Protocol of Fmoc-based Solid-phase Peptide Synthesis (Fmoc-SPPS)

……...……………………………………………………………………..… S4

B) Cleavage of fully protected crude peptide from resin………………………... S4

C) Preparation of hydrazine 2-chlorotrityl chloride resin…………………...…. S5

D) Preparation of peptide salicylaldehyde ester by "N+1" strategy……………. S5

II. Synthesis of HMGB1 and its Variants………………………......... S5

1. Synthesis of peptide segments……………………………………………...… S5

1.1 Synthesis of *1*……………………………………………………………...… S6

1.2 Synthesis of *14*……………………………………………………………….… S7

1.3 Synthesis of *2*……………………………………………………………...… S8

1.4 Synthesis of *3*…………………………………………………………………S9

1.5 Synthesis of *4*…………………………………………………………...…...S12

1.6 Synthesis of *15*………………………………………………………...…. S13

1.7 Synthesis of *5*……………………………………………………………… S15

1.8 Synthesis of *6*…………………………………………………………….… S16

2. Synthesis of N-terminus part of HMGB1 and its variants……………………. S17

2.1 Synthesis of *7*…………………………………………………………......…S17

2.2 Synthesis of *16*………………………………………………………...…. S20

2.3 Synthesis of *9*……………………………………………………….……… S23

2.4 Synthesis of *17*……………………………………………………….…..... S26

3. Synthesis of C-terminus part of HMGB1 and its variants………………….… S29

3.1 Synthesis of *10*………………………………………………………...….... S30

3.2 Synthesis of *11*………………………………………………………...…. S32

3.3 Synthesis of *19*…………………………………………………………………S35

3.4 Synthesis of *22*………………………………………………………………...… S39

4. Assembly of N and C-terminus part of HMGB1 and its variants into linear proteins……………………………………………………………………………… S41

4.1 Synthesis of *12*………………………………………………………...…… S41

4.2 Synthesis of *20a*……………………………………………………………… S44

4.3 Synthesis of *20b*……………………………………………………...……… S46

4.4 Synthesis of *20c*……………………………………………………...…… S49

4.5 Synthesis of *20e*………………………………………………………...… S52

4.6 Synthesis of *20d*…………………………………………………........ S55

4.7 Synthesis of *20f*……………………………………………...……...… S57

5. Protein Folding and Circular Dichroism (CD) Measurement……..……...… S59

III. Microscale Thermophoresis (MST) Binding Affinity Assay…... S61

IV. Cell Culture and Enzyme-Linked Immunosorbent Assay (ELISA)

…...…………………………………………………………………...… S61

V. Cell Migration Assay……………………………….……………...... S62

VI. Molecular Dynamics Simulations……………….…………...…..…… S63

VII. Binding Free Energy Calculations Using gmx_MMPBSA……. S64

VIII. Reference…………………………………………………...………. S65

I. General Experimental Procedures

A) Standard Protocol of Fmoc-based Solid-phase Peptide Synthesis (Fmoc-SPPS)

Peptides were synthesized manually by Fmoc-SPPS on 2-chlorotrityl chloride resin (GL Biochem or CSBio, resin loading: 0.5 mmol/g). Firstly, the resin was swollen in the dry dichloromethane (DCM) for 30min, then the solvent was washed away. The first amino acid FmocHN-Xaa-COOH (4.0 equiv.) and diisopropylethylamine (DIEA) (8.0 equiv.) was dissolved in DCM and added into the resin and shaking for 2~3 h to load the amino acid. The resin was then washed with DCM (5 mL × 3 times), dimethylformamide (DMF) (5 mL × 3 times) and DCM (5 mL × 3 times). Subsequently, a mixture of Methanol (MeOH) /DIEA/DCM (2:1:17, v/v/v) was added into the resin and shaking for 30 min to cap the resin. After capping, the resin was submitted to iterative peptide assembly (Fmoc-SPPS). The deblock solution was a mixture of piperidine/DMF (20:80, v/v). FmocHN-Trp(Boc)-COOH, FmocHN-Arg(Pbf)-COOH, FmocHN-Lys(Boc)-COOH, FmocHN-Glu(O*t*Bu)-COOH, FmocHN-Gln(Trt)-COOH, FmocHN-Thr(t Bu)-COOH, FmocHN-Ser(tBu)-COOH, FmocHN-Pro-COOH, FmocHN-Phe-COOH, FmocHN-Leu-COOH, FmocHN-Ile-COOH, FmocHNTyr(*t*Bu)-COOH, FmocHN-Asp(O*t*Bu)-COOH, FmocHN-Val-COOH, FmocHN-Gly-COOH, FmocHN-Met-COOH, FmocHN-Ala-COOH, FmocHN-Cys(S*t*Bu)-COOH, FmocHN-Cys(Trt)-COOH, FmocHN-His(Trt)-COOH, FmocHN-Asn(Trt)-COOH, BocHN-Gly-COOH, BocHN-Ser(*t*Bu)-COOH, BocHN-Thz-COOH, BocHNThr(*t*Bu)-COOH were used for coupling. FmocHN-QT(NBD)-COOAllyl building block was prepared and assembled into peptide sequence following the previously reported protocol [1]. Fmoc-Asn(Fuc-α1,6-GlcNAc)-COOH building block was prepared following the previously reported protocol [2]. For the coupling step, a solution of Fmoc protected amino acid or Boc protected amino acid (4.0 equiv. according to the resin capacity), 2-(7-Azabenzotriazol-1-yl)-N,N,N',N'-tetramethyluronium hexafluorophosphate (HATU) (4.0 equiv.) and DIEA (8 equiv.) in DMF was gently agitated with the resin at room temperature for 1-3h.

B) Cleavage of fully protected crude peptide from resin

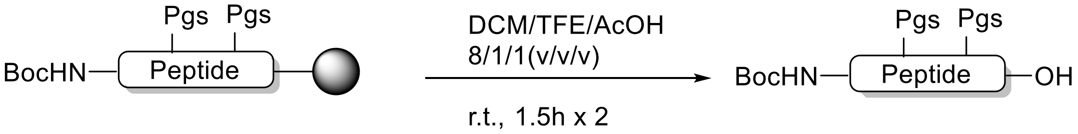

Figure S1. General scheme of soft cleavage.

The fully protected peptide on resin was treated with a cocktail of DCM/acetic acid (HOAc)/TFE=8:1:1 (v/v/v, 10-15 mL), 2 times and 1.5 h for each time. After cleavage, the resulting cleavage solutions were combined and concentrated to give crude protected peptide bearing the free carboxylic acid at the C-terminus.

C) Preparation of hydrazine 2-chlorotrityl chloride resin

2-chlorotrityl chloride resin (1 g, loading = ~0.5 mmol/g) was swelled in 10 mL DCM/DMF (1/1, v/v). Then 10 mL hydrazine monohydrate ($NH_2NH_2 \cdot H_2O$) /DMF (1/20, v/v) was added. The reaction was conducted for 30 min. 10 mL of methanol/DMF (1/20, v/v) was added to quench the remaining 2-chlorotrityl chloride resin. After 30 min, the resin was washed with DMF and DCM and ready for iterative peptide assembly (Fmoc-SPPS)

D) Preparation of peptide salicylaldehyde ester by "N+1" strategy

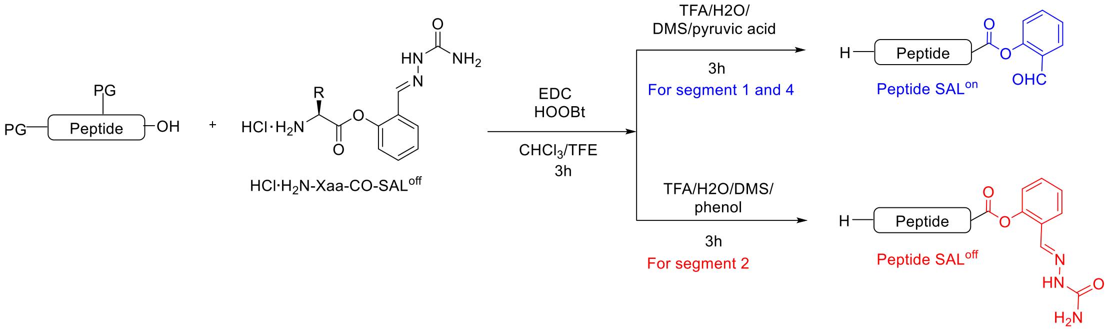

Figure S2. General scheme of "N+1" strategy.

Fully protected crude peptide (1.0 equiv., peptide concentration = 10 mM) and the corresponding L-Amino salicylaldehyde semicarbazone ester hydrochloride ($HCl \cdot H_2N$-Xaa-CO-SAL^{off}, 6 equiv.) was dissolved in chloroform ($CHCl_3$) / 2,2,2-trifluoroethanol (TFE) (9:1, v/v) and then Hydroxy-3,4-dihydro-4-oxo-1,2,3-benzotriazine (HOOBt) (3.0 equiv.) and N-(3- dimethylaminopropyl)-N'-ethylcarbodiimide (EDC) (3.0 equiv.) were added. The L-Amino salicylaldehyde semicarbazone ester hydrochloride was prepared following the previously reported protocols [3]. The reaction mixture was stirred at room temperature for 3h. The solvent was removed under vacuo. Then trifluoroacetic acid (TFA) /water (H_2O) /pyruvic acid/ dimethyl sulfide (DMS) (95/2.5/2.5/2.5, v/v/v/v) or TFA/H_2O/phenol/DMS (95/2.5/2.5/2.5, v/v/v/v) were added into the resulting residue, and the resulting mixture was stirred for 3 h at room temperature. After global protection, TFA was blown off and the residues was triturated with diethyl ether and then centrifuged. The crude peptide salicylaldehyde ester was dried under vacuum and purified by preparative reverse-phase HPLC (High Performance Liquid Chromatography).

II. Synthesis of linear HMGB1 and its variants

1. Synthesis of peptide segments

1.1 Synthesis of *1*

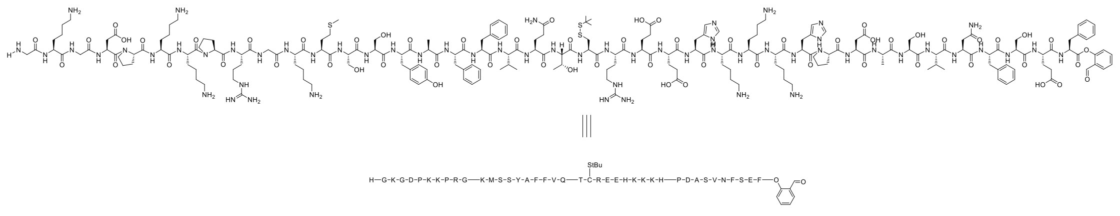

Figure S3. Chemical structure of ***1***.

Peptidyl salicylaldehyde ester ***1*** was synthesized according to ***General Experimental Procedure I-D***. The crude peptide salicylaldehyde ester was purified by preparative reverse-phase HPLC (20 to 70% Acetonenitrile (ACN) /H_2O over 45 min, 0.1% TFA). After lyophilization, 106 mg ***1*** (from 0.08mmol soft-cleaved crude peptide) was obtained in an isolated yield of 27.6%.

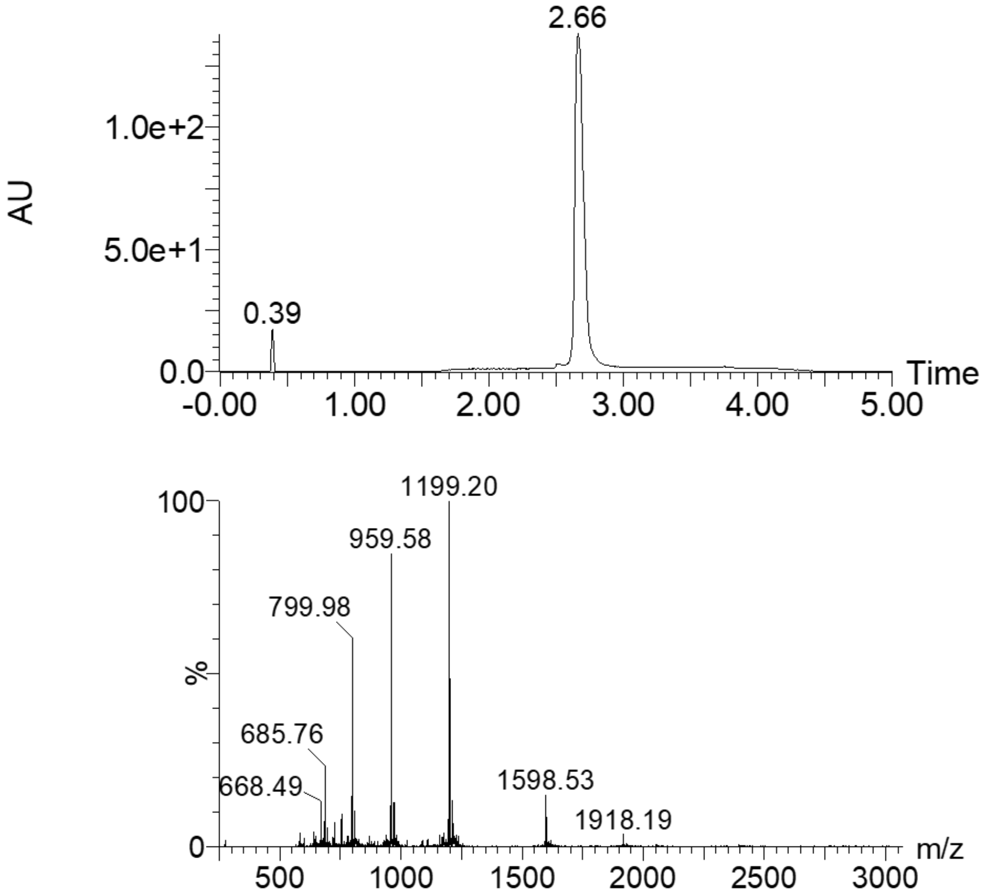

Figure S4. UV trace from analytical RP-UPLC (Reverse-phase Ultra Performance Liquid Chromatography) and its ESI-MS (Electrospray Ionization-Mass Spectrometry) of ***1***. Gradient: 5-95% ACN/H_2O containing 0.1% TFA over 5 min at a flow rate of 0.4 mL/min. ESI-MS calculated for $C_{215}H_{325}N_{59}O_{60}S_3$ $[M+3H]^{3+}$ m/z = 1598.50, found 1598.53, $[M+4H]^{4+}$ m/z = 1199.13, found 1199.20, $[M+5H]^{5+}$ m/z = 959.50, found 959.58, $[M+6H]^{6+}$ m/z = 799.75, found 799.98, $[M+7H]^{7+}$

m/z = 685.64, found 685.76.

1.2 Synthesis of *14*

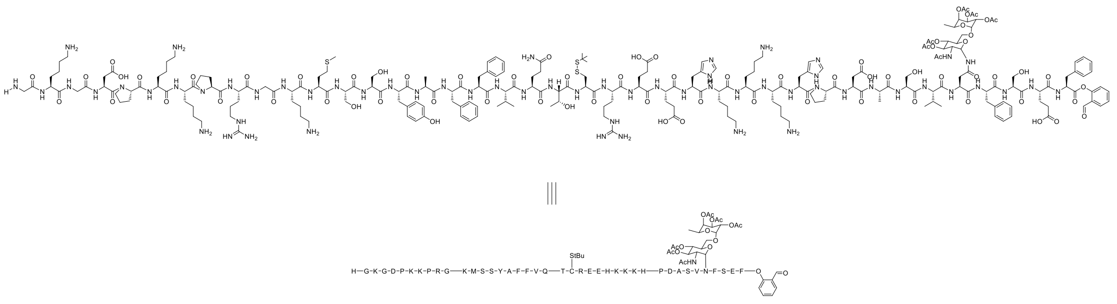

Figure S5. Chemical structure of ***14***.

Peptidyl salicylaldehyde ester ***14*** was synthesized according to ***General Experimental Procedure I-D***. The crude peptide salicylaldehyde ester was purified by preparative reverse-phase HPLC (20 to 70% ACN/H_2O over 45 min, 0.1% TFA). After lyophilization, 180 mg ***14*** (from 0.160mmol soft-cleaved crude peptide) was obtained in an isolated yield of 21.0%.

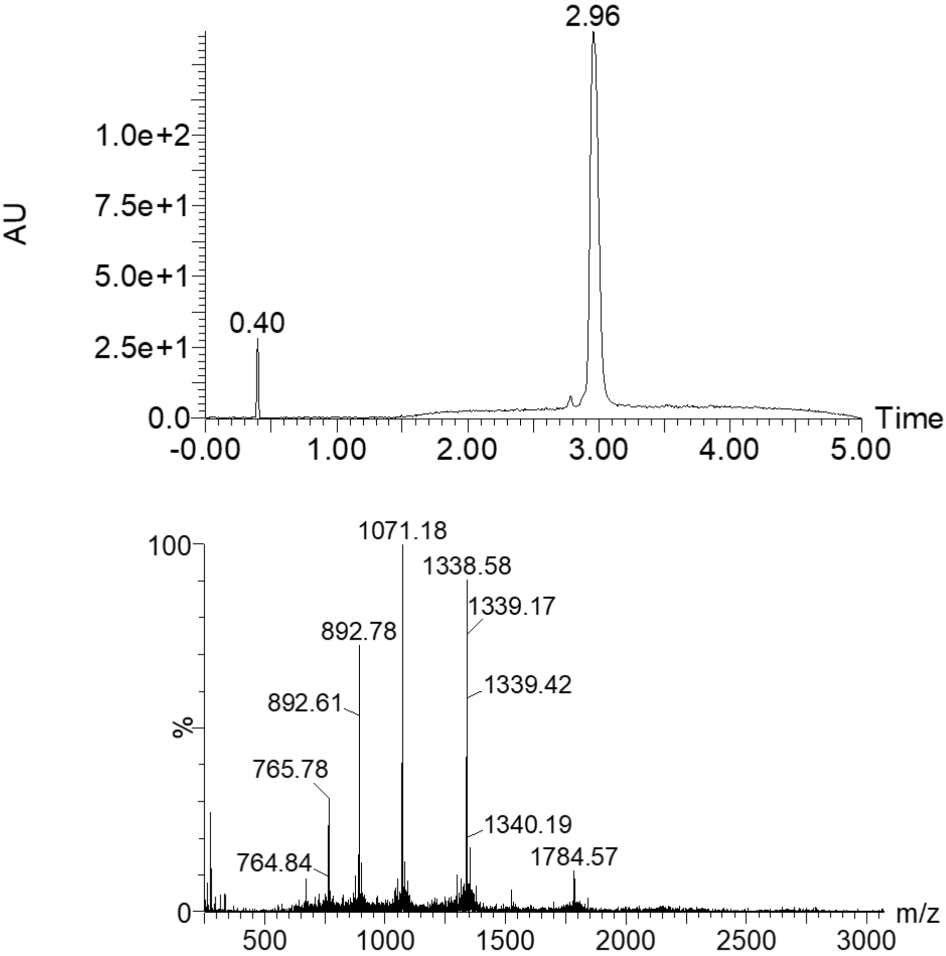

Figure S6. UV trace from analytical RP-UPLC and its ESI-MS of ***14***. Gradient: 5-95% ACN/H_2O containing 0.1% TFA over 5 min at a flow rate of 0.4 mL/min. ESI-MS calculated for $C_{239}H_{358}N_{60}O_{74}S_3$ $[M+3H]^{3+}$ m/z = 1785.01, found 1784.57, $[M+4H]^{4+}$ m/z = 1339.01, found 1338.58, $[M+5H]^{5+}$ m/z = 1071.40, found 1071.18, $[M+6H]^{6+}$ m/z = 893.00, found 892.78, $[M+7H]^{7+}$ m/z = 765.57, found 765.78.

1.3 Synthesis of *2*

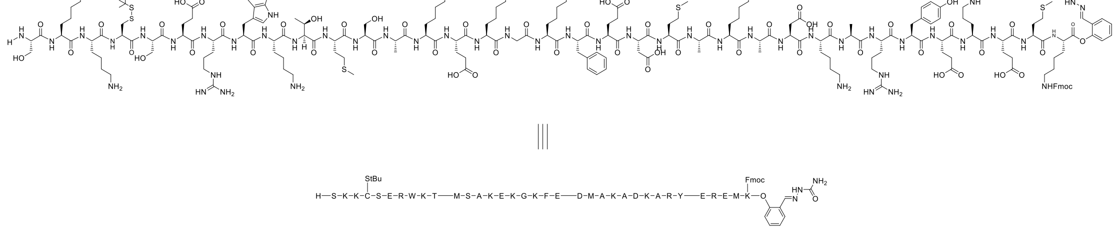

Figure S7. Chemical structure of ***2***.

Peptidyl salicylaldehyde ester ***2*** was synthesized according to ***General Experimental Procedure I-D***. The crude peptide salicylaldehyde ester was purified by preparative reverse-phase HPLC using gradient of 20% ACN/H_2O to 60% ACN/H_2O over 45 min. After lyophilization, 32 mg ***2*** (from 0.040mmol soft-cleaved crude peptide) was obtained in an isolated yield of 17.1 %.

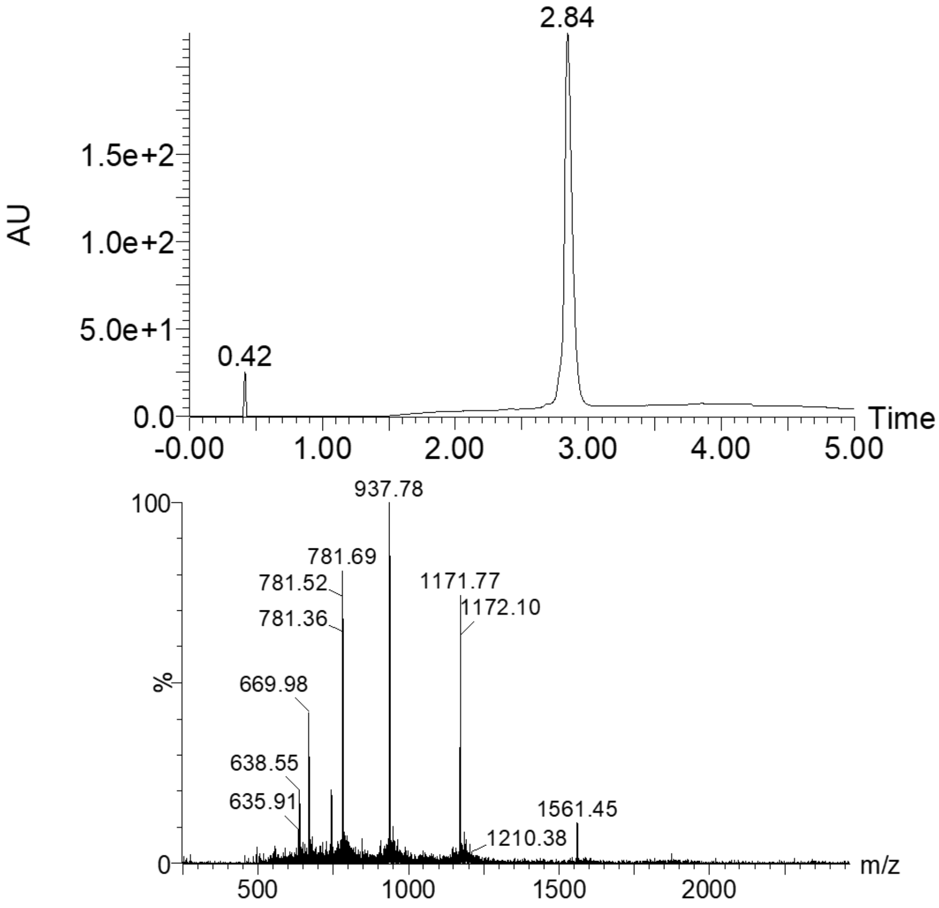

Figure S8. UV trace from analytical RP-UPLC and its ESI-MS of ***2***. Gradient: 5-95% ACN/H_2O containing 0.1% TFA over 5 min at a flow rate of 0.4 mL/min. ESI-MS calculated for $C_{206}H_{321}N_{57}O_{58}S_5$ $[M+3H]^{3+}$ m/z = 1562.49, found 1561.45, $[M+4H]^{4+}$ m/z = 1172.12, found 1171.77, $[M+5H]^{5+}$ m/z = 937.90, found 937.78, $[M+6H]^{6+}$ m/z = 781.75, found 781.69, $[M+7H]^{7+}$ m/z = 670.21, found 669.98.

1.4 Synthesis of *3*

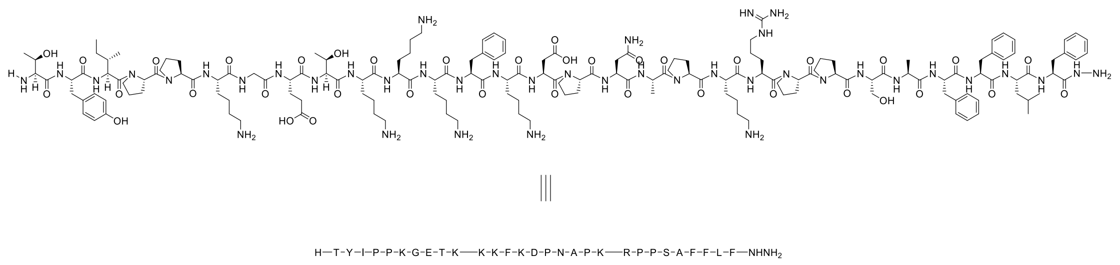

Figure S9. Chemical structure of ***3****.

Peptide hydrazide ***3**** was synthesized according to the ***General Experimental Procedure I-A*** using hydrazine 2-chlorotrityl chloride resin (prepared according to ***General Experimental Procedure I-C***). After Fmoc-SPPS, TFA/H_2O/ Triisopropylsilane (TIS) (95/2.5/2.5, v/v/v) was added to cleavage the peptide from resin and globally deprotect for 2 h at room temperature. The crude peptide was purified by preparative reverse-phase HPLC (10 to 50% ACN/H_2O over 45 min, 0.1% TFA) and then lyophilized as a white solid.

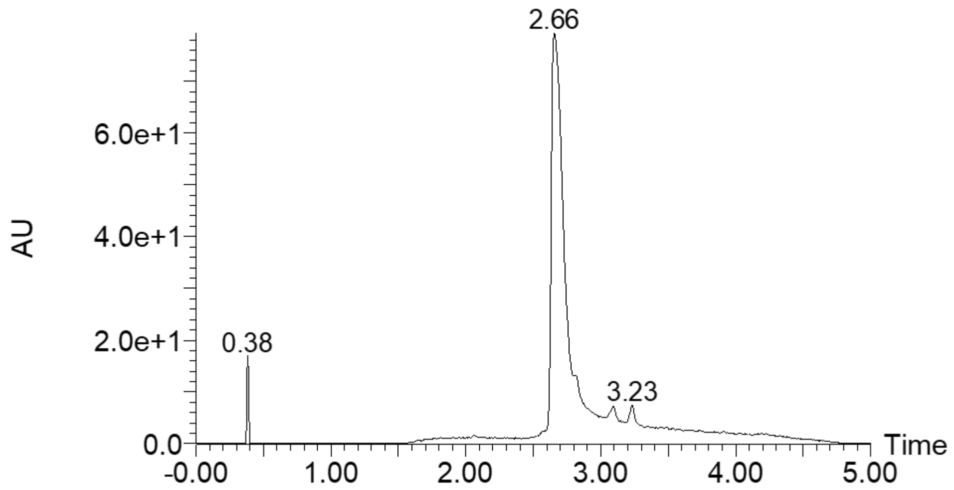

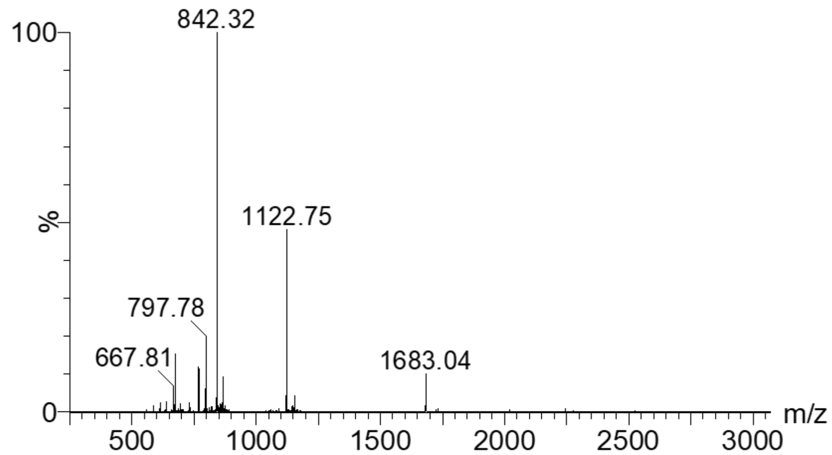

Figure S10. UV trace from analytical RP-UPLC and its ESI-MS of 3*. Gradient: 5-95% ACN/H_2O containing 0.1% TFA over 5 min at a flow rate of 0.4 mL/min. ESI-MS calculated for $C_{161}H_{247}N_{41}O_{38}$ $[M+2H]^{2+}$ m/z = 1683.50, found 1683.04, $[M+3H]^{3+}$ m/z = 1122.67, found 1122.75, $[M+4H]^{4+}$ m/z = 842.25, found 842.32.

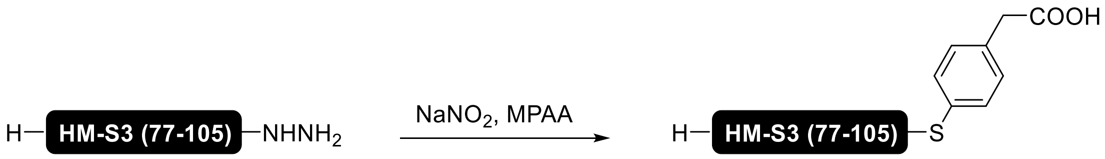

Figure S11. Transform of peptidyl hydrazide ***3**** to MPAA ester ***3***.

3* (1 eq., 2.5mM) was dissolved in aqueous buffer containing 6 M Gn-HCl and 0.2 M NaH_2PO_4 (pH = 3.0) and cooled to approximately -15°C in an ice-salt bath. 0.5 M $NaNO_2$ (10eq.) was then added to activate the peptide hydrazide and stirred at -15°C for 15 min. After that, 4-mercaptophenylacetic acid (MPAA, 100eq. ,250mM) was dissolved in 0.2 M NaH_2PO_4 solution containing 6 M Gn·HCl (pH 7.0), then this solution was added into the above mixture and stirred at room temperature for 30min. After the reaction completed, peptide MPAA thioester ***3*** was purified by preparative reverse-phase HPLC (10 to 50% ACN/H_2O over 45 min, 0.1% TFA) and then lyophilized as a white solid.

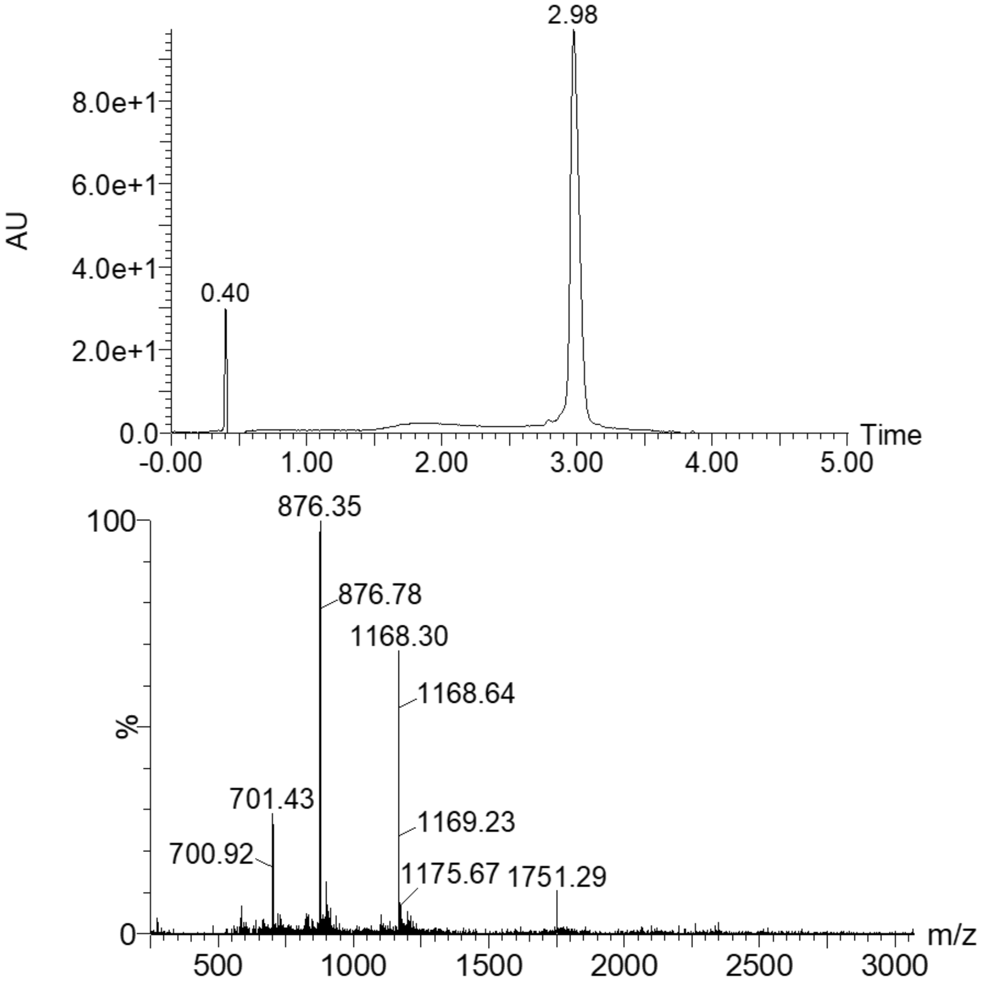

Figure S12. UV trace from analytical RP-UPLC and its ESI-MS of ***3***. Gradient: 5-95% ACN/H_2O containing 0.1% TFA over 5 min at a flow rate of 0.4 mL/min. ESI-MS calculated for $C_{169}H_{251}N_{39}O_{40}S$ $[M+2H]^{2+}$ m/z = 1751.58, found 1751.29, $[M+3H]^{3+}$ m/z = 1168.05, found 1168.30, $[M+4H]^{4+}$ m/z = 876.29, found 876.35, $[M+5H]^{5+}$ m/z = 701.23, found 701.43.

1.5 Synthesis of *4*

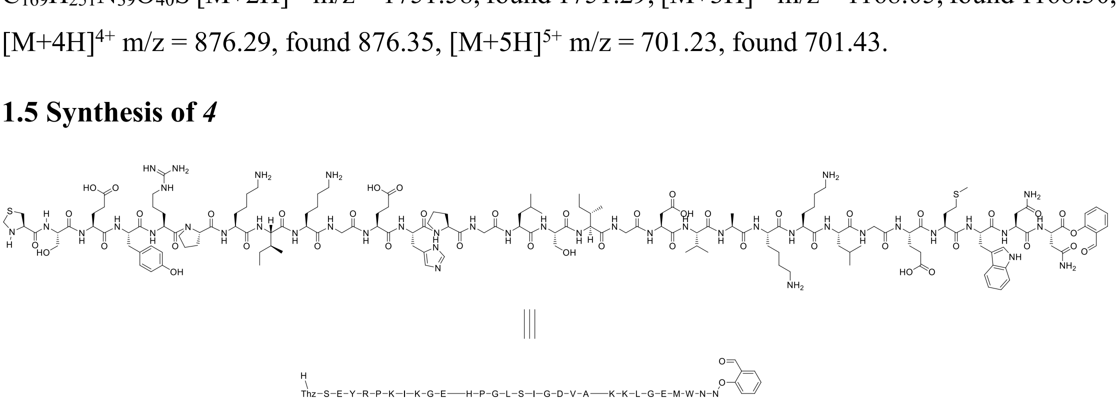

Figure S13. Chemical structure of ***4***.

Peptidyl salicylaldehyde ester ***4*** was synthesized according to ***General Experimental Procedure I-D***. The crude peptide salicylaldehyde ester was purified by preparative reverse-phase HPLC using gradient of 20% ACN/H_2O to 60% ACN/H_2O over 45 min. After lyophilization, 68 mg ***4*** (from 0.060mmol soft-cleaved crude peptide) was obtained in an isolated yield of 32.6 %.

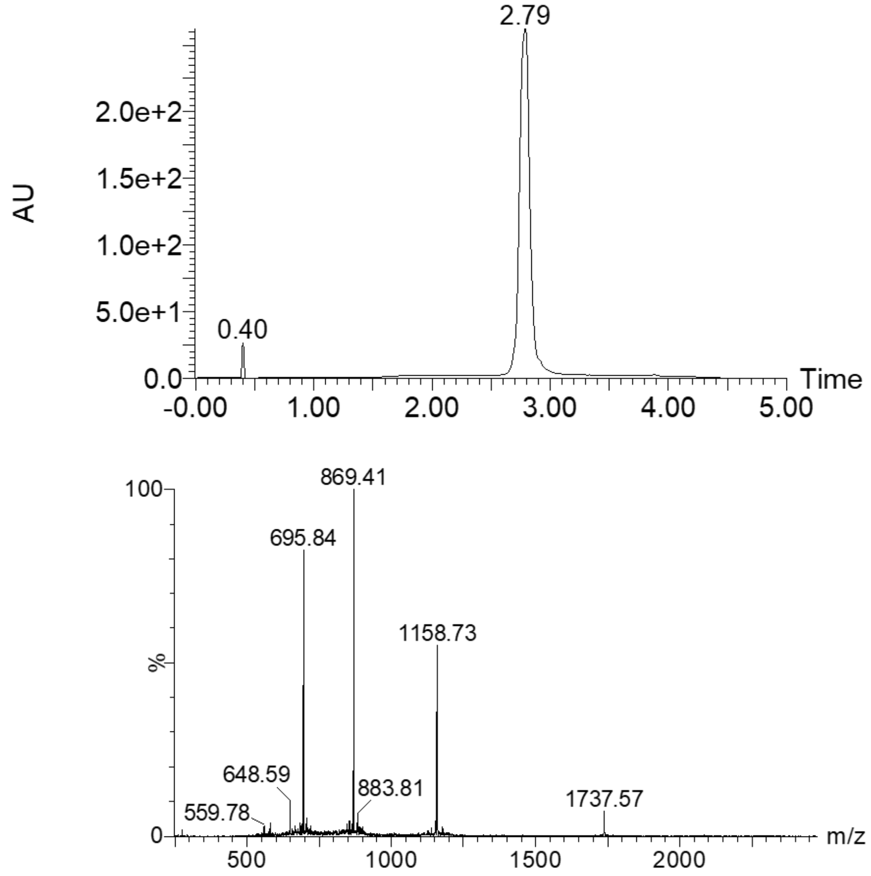

Figure S14. UV trace from analytical RP-UPLC and its ESI-MS of ***4***. Gradient: 5-95% ACN/H_2O containing 0.1% TFA over 5 min at a flow rate of 0.4 mL/min. ESI-MS calculated for $C_{155}H_{238}N_{42}O_{45}S_2$ $[M+2H]^{2+}$ m/z = 1737.99, found 1737.57, $[M+3H]^{3+}$ m/z = 1158.99, found 1158.73, $[M+4H]^{4+}$ m/z = 869.50, found 869.41, $[M+5H]^{5+}$ m/z = 695.80, found 695.84.

1.6 Synthesis of *15*

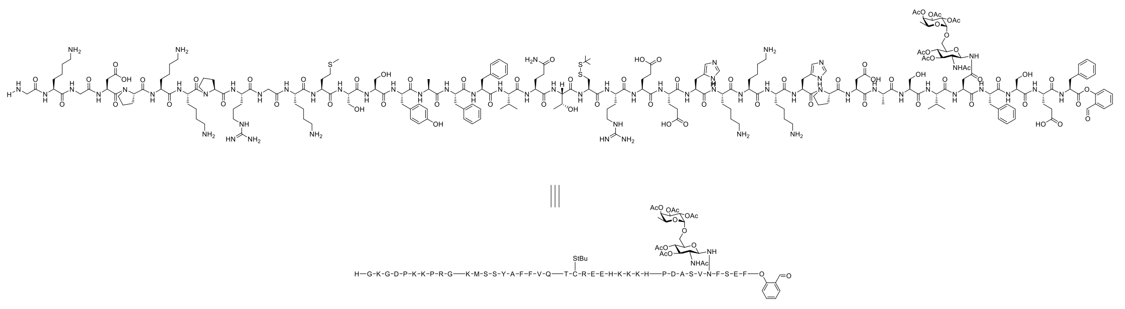

Figure S15. Chemical structure of ***15.***

Peptidyl salicylaldehyde ester ***15*** was synthesized according to ***General Experimental Procedure I-D***. The crude peptide salicylaldehyde ester was purified by preparative reverse-phase HPLC using gradient of 20% ACN/H_2O to 60% ACN/H_2O over 45 min. After lyophilization, 92 mg ***15*** (from 0.080mmol soft-cleaved crude peptide) was obtained in an isolated yield of 28.5 %.

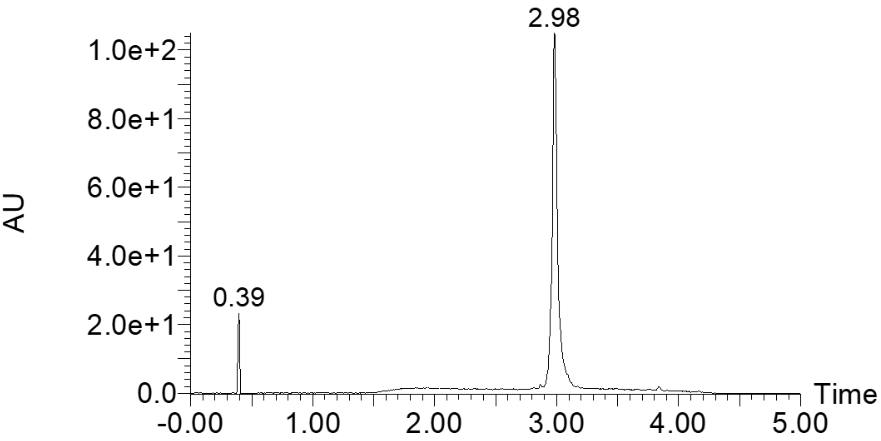

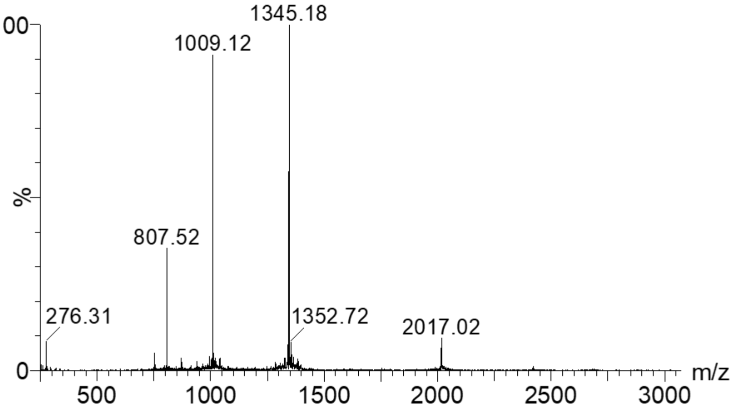

Figure S16. UV trace from analytical RP-UPLC and its ESI-MS of ***15***. Gradient: 5-95% ACN/H_2O containing 0.1% TFA over 5 min at a flow rate of 0.4 mL/min. ESI-MS calculated for $C_{179}H_{271}N_{43}O_{59}S_2$ $[M+2H]^{2+}$ m/z = 2017.75, found 2017.02, $[M+3H]^{3+}$ m/z = 1345.50, found 1345.18, $[M+4H]^{4+}$ m/z = 1009.38, found 1009.12, $[M+5H]^{5+}$ m/z = 807.70, found 807.52.

1.7 Synthesis of *5*

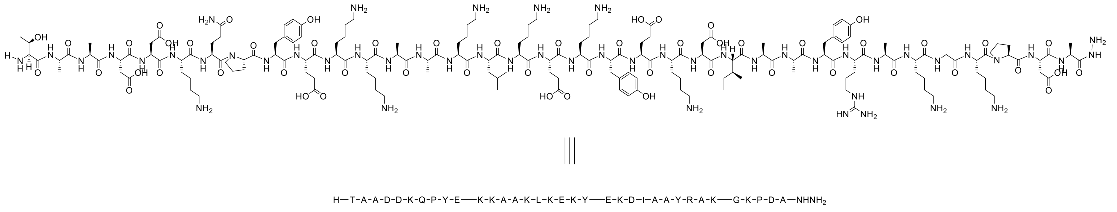

Figure S17. Chemical structure of **5**.

Peptide hydrazide ***5*** was synthesized according to the ***General Experimental Procedure I-A*** using hydrazine 2-chlorotrityl chloride resin (prepared according to ***General Experimental Procedure I-C***). After Fmoc-SPPS, TFA/H_2O/TIS (95/2.5/2.5, v/v/v) was added to cleavage the peptide from resin and globally deprotect for 2 h at room temperature. The crude peptide was purified by preparative reverse-phase HPLC (10 to 50% ACN/H_2O over 45 min, 0.1% TFA) and then lyophilized as a white solid.

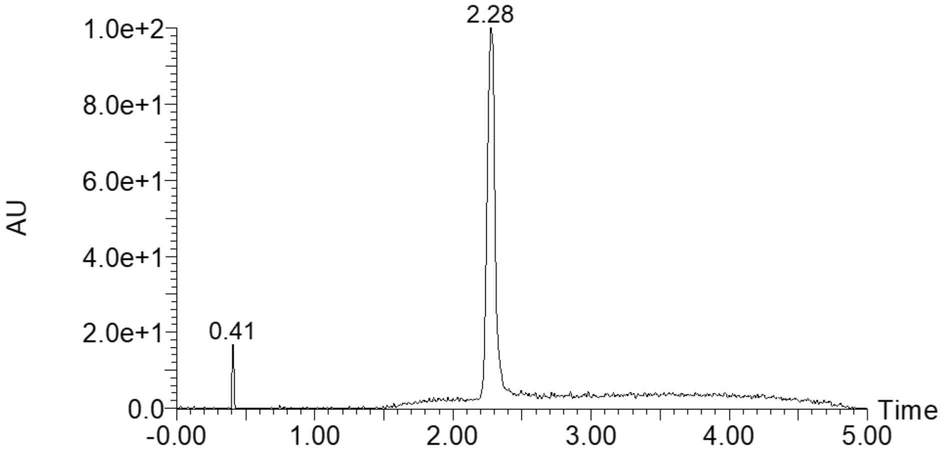

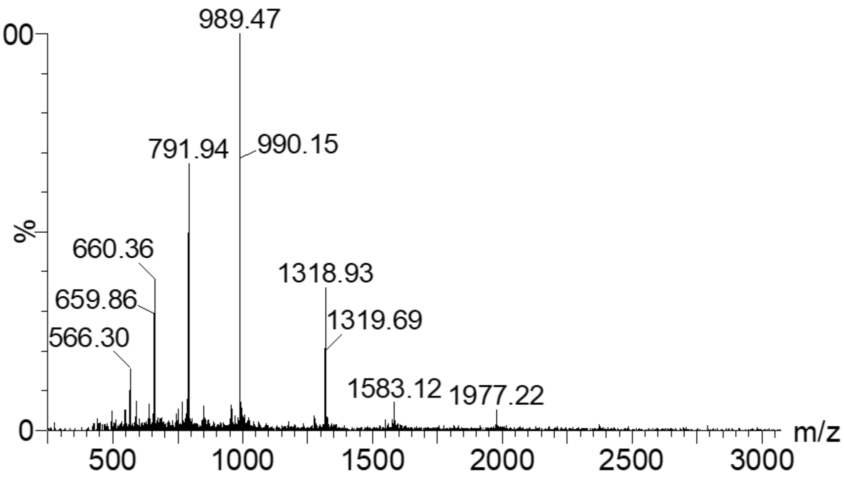

Figure S18. UV trace from analytical RP-UPLC and its ESI-MS of ***5***. Gradient: 5-95% ACN/H_2O containing 0.1% TFA over 5 min at a flow rate of 0.4 mL/min. ESI-MS calculated for $C_{175}H_{286}N_{50}O_{54}$ $[M+2H]^{2+}$ m/z = 1978.26, found 1977.22, $[M+3H]^{3+}$ m/z = 1319.17, found 1318.93, $[M+4H]^{4+}$ m/z = 989.63, found 989.47, $[M+5H]^{5+}$ m/z = 791.90, found 791.94, $[M+6H]^{6+}$ m/z = 660.08, found 660.36.

1.8 Synthesis of *6*

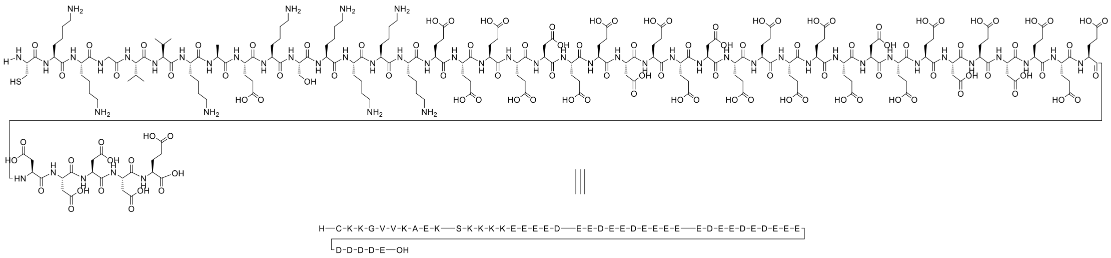

Figure S19. Chemical structure of ***6***.

Peptidyl carboxylic acid ***6*** was synthesized according to the ***General Experimental Procedure I-A*** using 2-chlorotrityl chloride resin. After Fmoc-SPPS, TFA/H_2O/1,2-ethanedithiol (EDT)/TIS (95/2.5/2.5/2.5, v/v/v/V) was added to cleavage the peptide from resin and globally deprotect for 2 h at room temperature. The crude peptide was purified by preparative reverse-phase HPLC (10 to 40% ACN/H2O over 45 min, 0.1% TFA) and then lyophilized as a white solid.

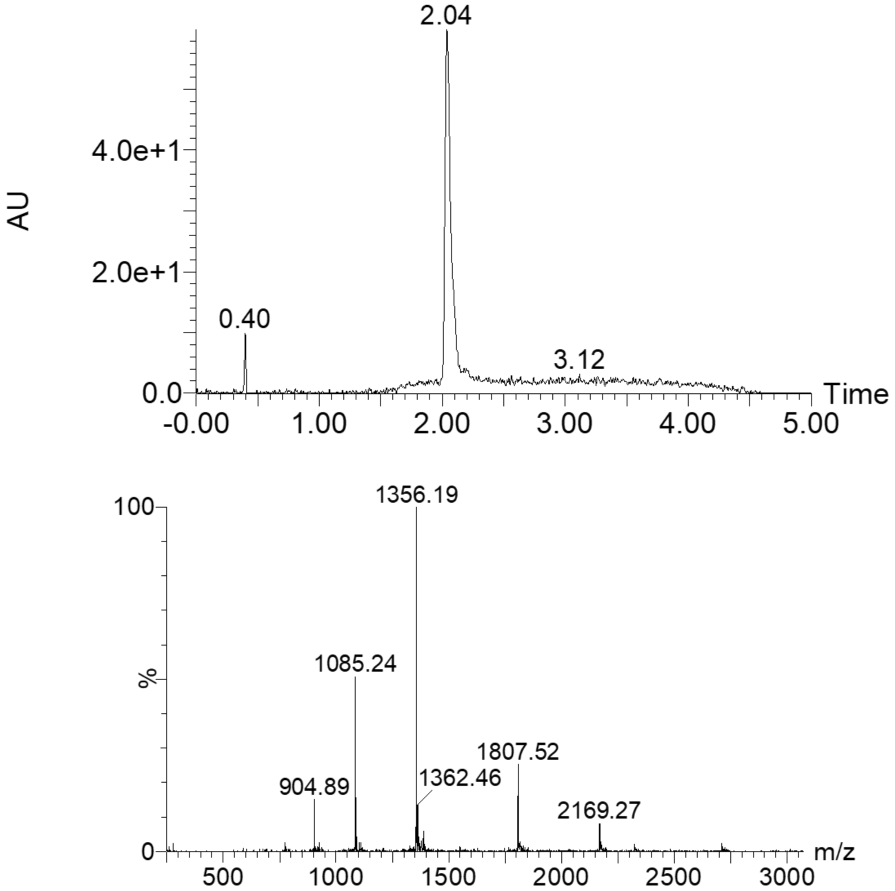

Figure S20. UV trace from analytical RP-UPLC and its ESI-MS of ***6***. Gradient: 5-95% ACN/H_2O containing 0.1% TFA over 5 min at a flow rate of 0.4 mL/min. ESI-MS calcd. for $C_{214}H_{331}N_{53}O_{109}S$ $[M+3H]^{3+}$ m/z = 1808.44, found 1807.52, $[M+4H]^{4+}$ m/z = 1356.58, found 1356.19, $[M+5H]^{5+}$ m/z = 1085.46, found 1085.24, $[M+6H]^{6+}$ m/z = 904.72, found 904.89.

2. Synthesis of N-terminus part of HMGB1 and its variants

2.1 Synthesis of *7*

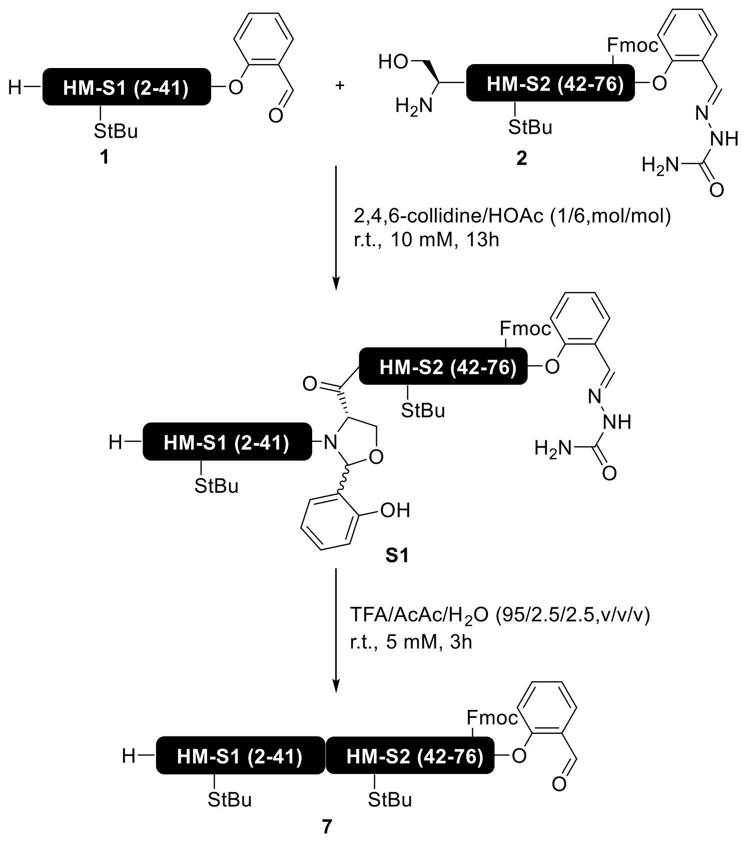

Figure S21. Synthetic route of *7*.

Peptide salicylaldehyde ester ***1*** (71.4 mg, 14.9 μmol) and peptide salicylaldehyde ester ***2*** (50.7 mg, 10.8 μmol) were dissolved in 2,4,6-collidine/HOAc (1/6, mol/mol) at a concentration of 10 mM at room temperature. The reaction mixture was stirred at room temperature for 13h to afford ***S1***. Then, the solvent was removed under condensed air and washed with diethyl ether and centrifuged. The residue was treated with TFA/H_2O/Acetylacetone (AcAc) (95/2.5/2.5, v/v/v) at a concentration of 5 mM for 3h to afford *7*. Then, TFA was blown off and the reside was washed with diethyl ether and centrifuged. The crude ligation product was purified by preparative reverse-phase HPLC (20 to 70% ACN/H_2O over 45 min, 0.1% TFA). After lyophilization, 6 mg *7* was obtained as a white solid in an isolated yield of 24.9%.

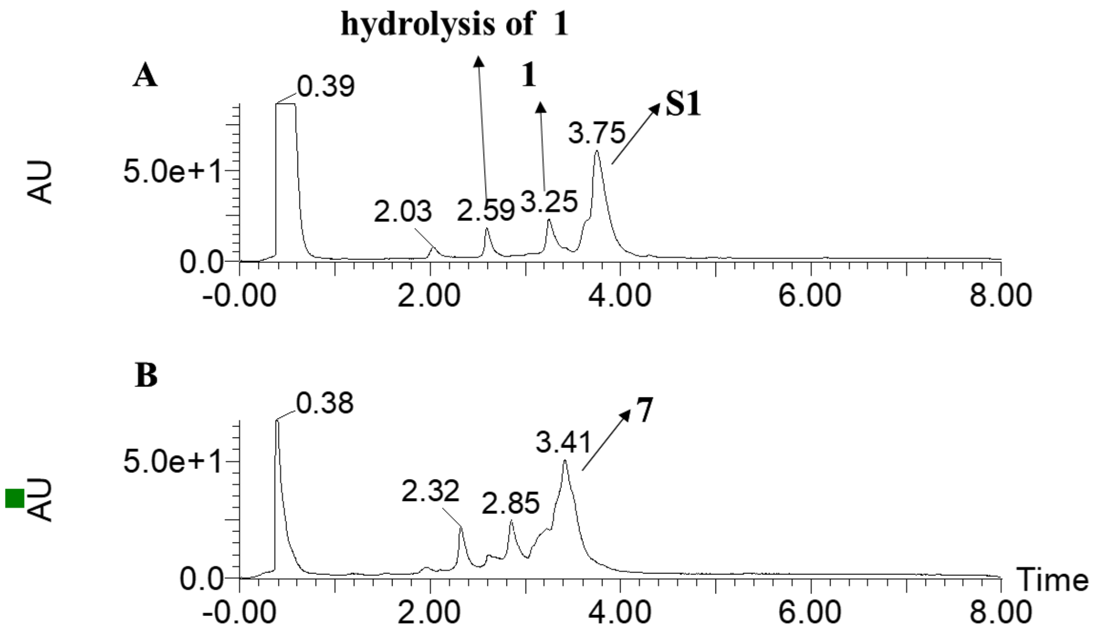

Figure S22. UV trace from analytical RP-UPLC of serine ligation reaction mixture between ***1*** and ***2***. (A) Serine ligation at 13h. Gradient: 20-60% ACN/H_2O containing 0.1% TFA over 8 min at a flow rate of 0.4 mL/min. (B) Acidolysis at 3h. Gradient: 20-70 % ACN/H_2O containing 0.1% TFA over 8 min at a flow rate of 0.4 mL/min.

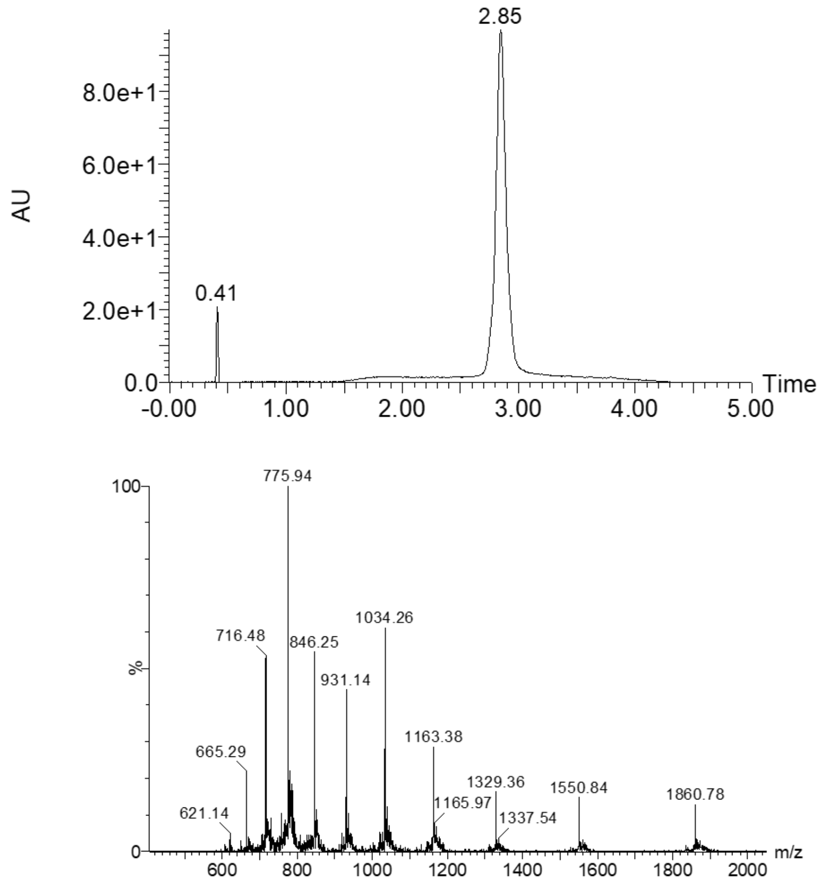

Figure S23. UV trace from analytical RP-UPLC and its ESI-MS of *7*. Gradient: 5-95% ACN/H_2O containing 0.1% TFA over 5 min at a flow rate of 0.4 mL/min. ESI-MS calculated for $C_{413}H_{637}N_{113}O_{116}S_8$ $[M+5H]^{5+}$ m/z = 1860.56, found 1860.78, $[M+6H]^{6+}$ m/z = 1550.63, found 1550.84, $[M+7H]^{7+}$ m/z = 1329.26, found 1329.36, $[M+8H]^{8+}$ m/z = 1163.22, found 1163.38, $[M+9H]^{9+}$ m/z = 1034.09, found 1034.26, $[M+10H]^{10+}$ m/z = 930.78, found 931.14, $[M+11H]^{11+}$ m/z = 846.25, found 846.25, $[M+12H]^{12+}$ m/z = 775.82, found 775.94, $[M+13H]^{13+}$ m/z = 716.21, found 716.48, $[M+14H]^{14+}$ m/z = 665.13, found 665.29.

2.2 Synthesis of *16*

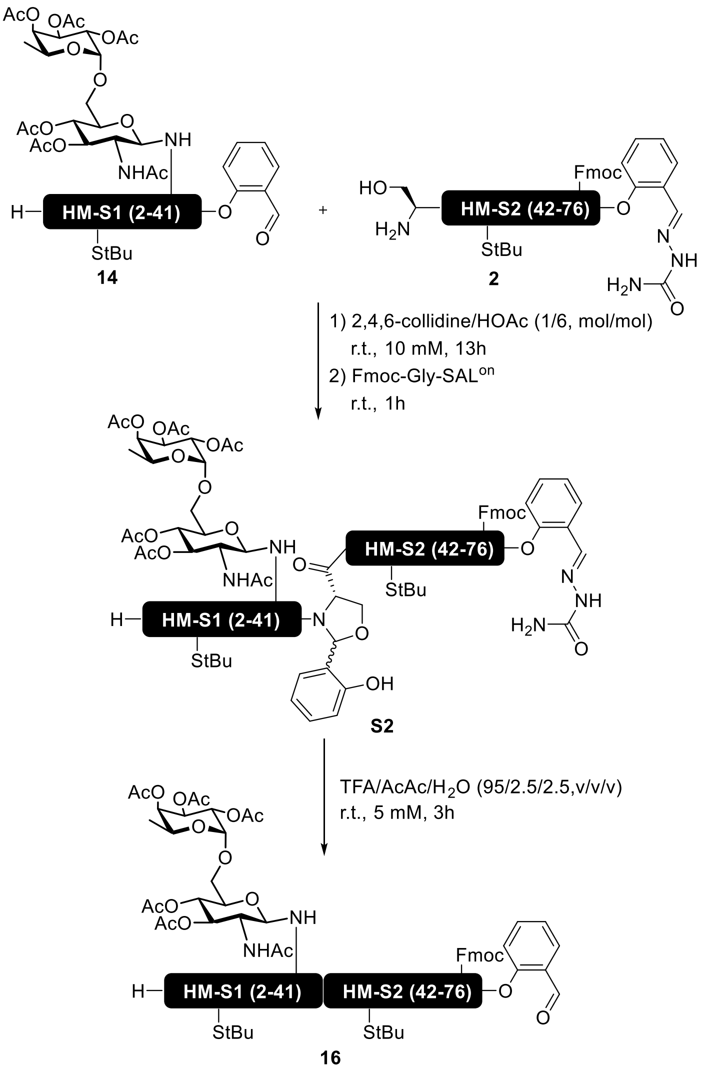

Figure S24. Synthetic route of ***16***.

Peptide salicylaldehyde ester ***14*** (126 mg, 23.5 µmol) and peptide salicylaldehyde ester ***2*** (100 mg, 21.4 µmol) were dissolved in 2,4,6-collidine/HOAc (1/6, mol/mol) at a concentration of 10 mM at room temperature. The reaction mixture was stirred at room temperature for 13h to afford ***S2***. After that, 10 equiv. Fmoc-Gly-SAL^{on} was dissolved in same amount of ligation buffer and added to the reaction for 1h to consume the unreacted ***2***. Then, the solvent was removed under condensed air and washed with diethyl ether and centrifuged. The residue was treated with TFA/H_2O/AcAc (95/2.5/2.5, v/v/v) at a concentration of 5 mM for 3h to afford ***16***. Then, TFA was blown off and the reside was washed with diethyl ether and centrifuged. The crude ligation product was purified by preparative reverse-phase HPLC (20 to 70% ACN/H_2O over 45 min, 0.1% TFA). After lyophilization, 40.7 mg ***16*** was obtained as a white solid in an isolated yield of 19.3%.

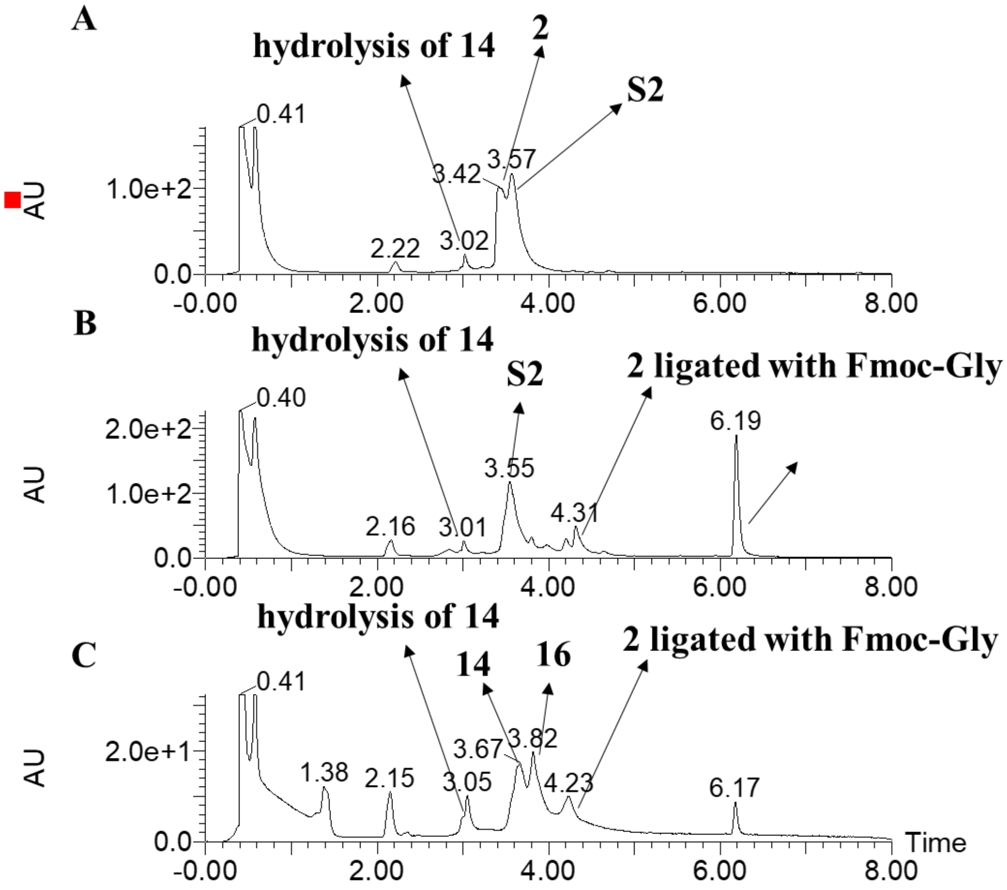

Figure S25. UV trace from analytical RP-UPLC of serine ligation reaction mixture between ***14*** and ***2***. (A) Serine ligation at 13h. Gradient: 20-70% ACN/H_2O containing 0.1% TFA over 8 min at a flow rate of 0.4 mL/min. (B) Adding Fmoc-Gly-SAL^{on} at 1h. Gradient: 20-70% ACN/H_2O containing 0.1% TFA over 8 min at a flow rate of 0.4 mL/min. (C) Acidolysis at 3h. Gradient: 20-70 % ACN/H_2O containing 0.1% TFA over 8 min at a flow rate of 0.4 mL/min.

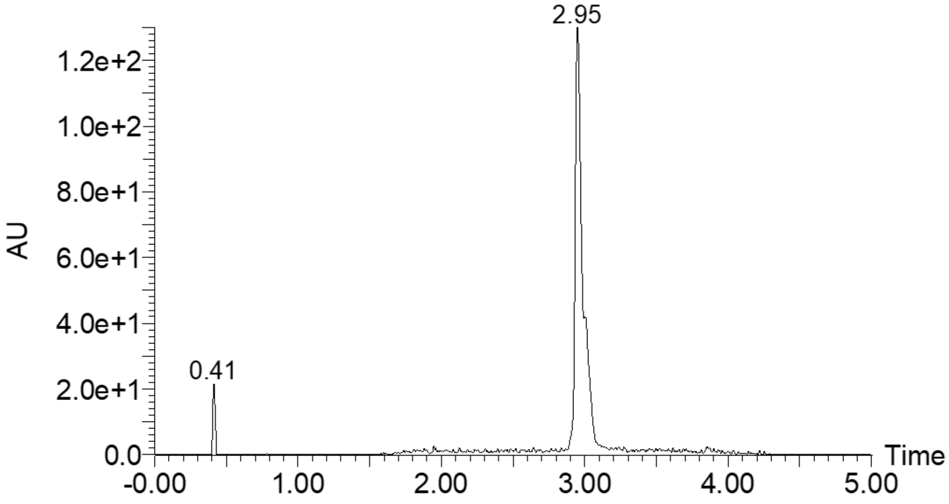

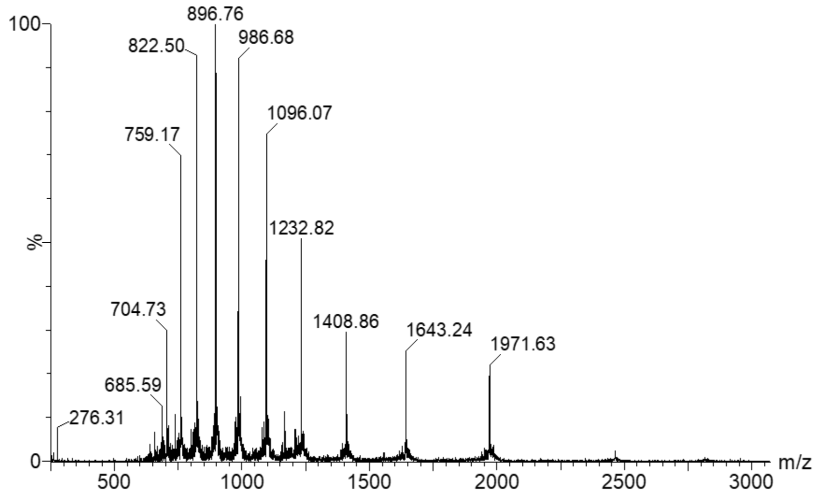

Figure S26. UV trace from analytical RP-UPLC and its ESI-MS of ***16***. Gradient: 5-95% ACN/H_2O containing 0.1% TFA over 5 min at a flow rate of 0.4 mL/min. ESI-MS calculated for $C_{437}H_{670}N_{114}O_{130}S_8$ $[M+5H]^{5+}$ m/z = 1972.46, found 1971.63, $[M+6H]^{6+}$ m/z = 1643.89, found 1643.24, $[M+7H]^{7+}$ m/z = 1409.19, found 1408.86, $[M+8H]^{8+}$ m/z = 1233.17, found 1232.82, $[M+9H]^{9+}$ m/z = 1096.26, found 1096.07, $[M+10H]^{10+}$ m/z = 986.73, found 986.68, $[M+11H]^{11+}$ m/z = 897.12, found 896.76, $[M+12H]^{12+}$ m/z = 822.44, found 822.50, $[M+13H]^{13+}$ m/z = 759.26, found 759.17, $[M+14H]^{14+}$ m/z = 705.09, found 704.73.

2.3 Synthesis of *9*

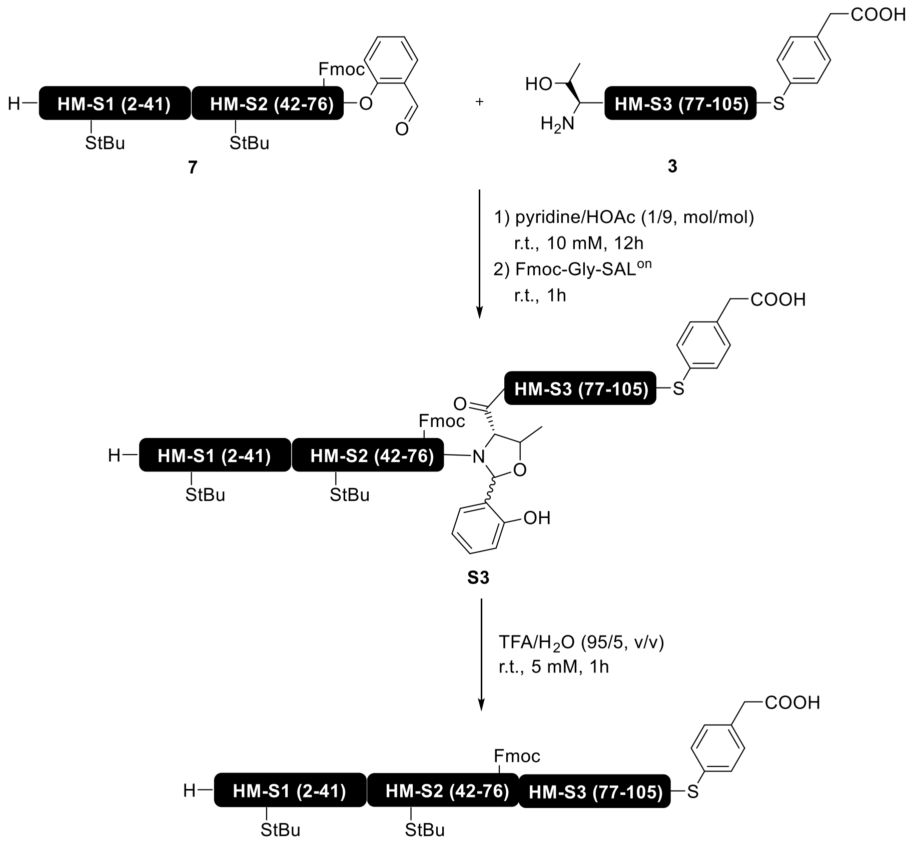

Figure S27. Synthetic route of ***9***.

Peptide salicylaldehyde ester *7* (27.0 mg, 2.9 μmol) and peptide MPAA ester ***3*** (13.2 mg, 3.8 μmol) were dissolved in pyridine/HOAc (1/9, mol/mol) at a concentration of 10 mM at room temperature. The reaction mixture was stirred at room temperature for 13h to afford ***S3***. After that, 10 equiv. Fmoc-Gly-SAL^{on} was dissolved in same amount of ligation buffer and added to the reaction for 1h to consume the unreacted ***3***. Then, the solvent was removed under condensed air and washed with diethyl ether and centrifuged. The residue was treated with TFA/H_2O (95/5, v/v) at a concentration of 5 mM for 1h to afford ***9***. Then, TFA was blown off and the reside was washed with diethyl ether and centrifuged. The crude ligation product was purified by preparative reverse-phase HPLC (20 to 70% ACN/H_2O over 45 min, 0.1% TFA). After lyophilization, 10.5 mg ***9*** was obtained as a white solid in an isolated yield of 28.6%.

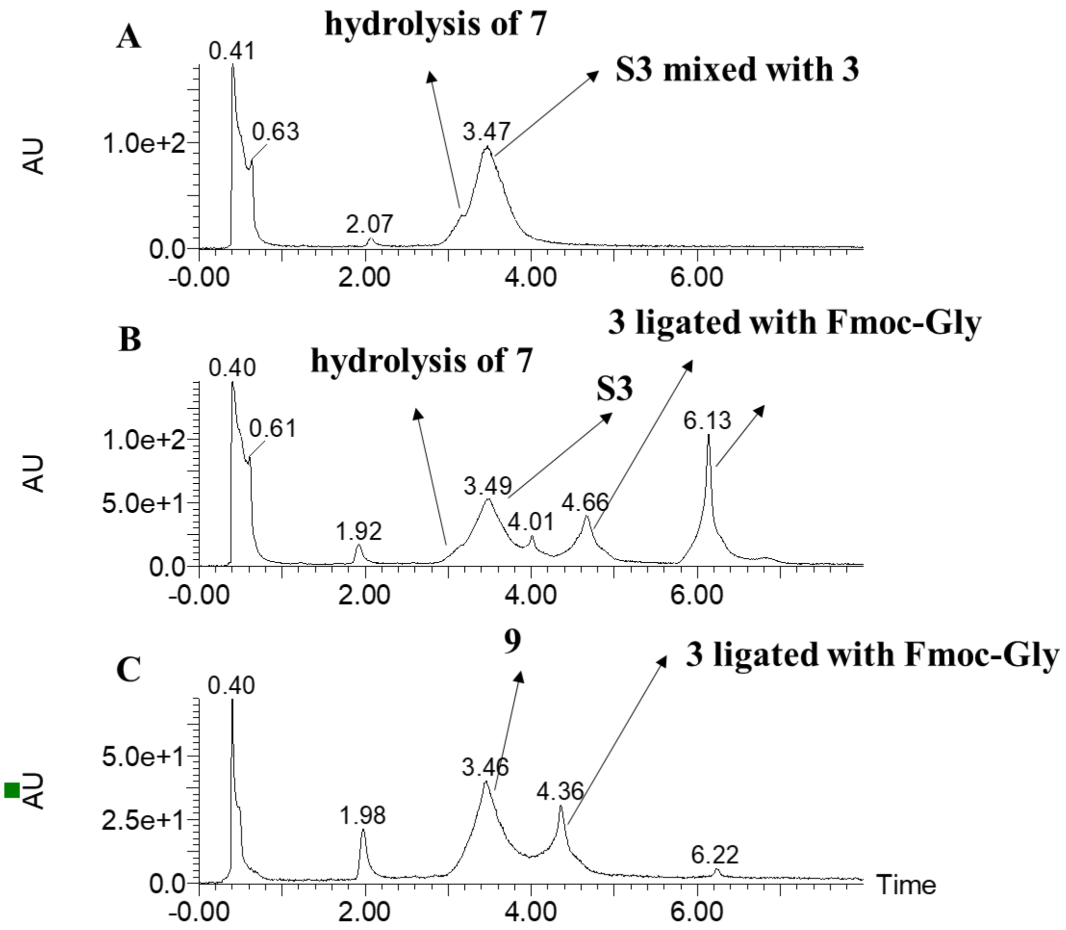

Figure S28. UV trace from analytical RP-UPLC of threonine ligation reaction mixture between *7* and *3*. (A) Serine ligation at 12h. Gradient: 20-70% ACN/H_2O containing 0.1% TFA over 8 min at a flow rate of 0.4 mL/min. (B) Adding Fmoc-Gly-SAL^{on} at 1h. Gradient: 20-70% ACN/H_2O containing 0.1% TFA over 8 min at a flow rate of 0.4 mL/min. (C) Acidolysis at 1h. Gradient: 20-70 % ACN/H_2O containing 0.1% TFA over 8 min at a flow rate of 0.4 mL/min.

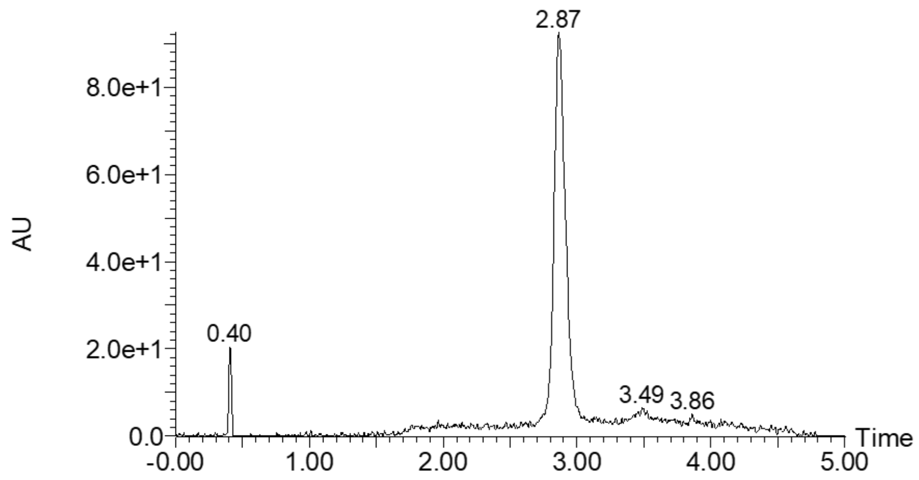

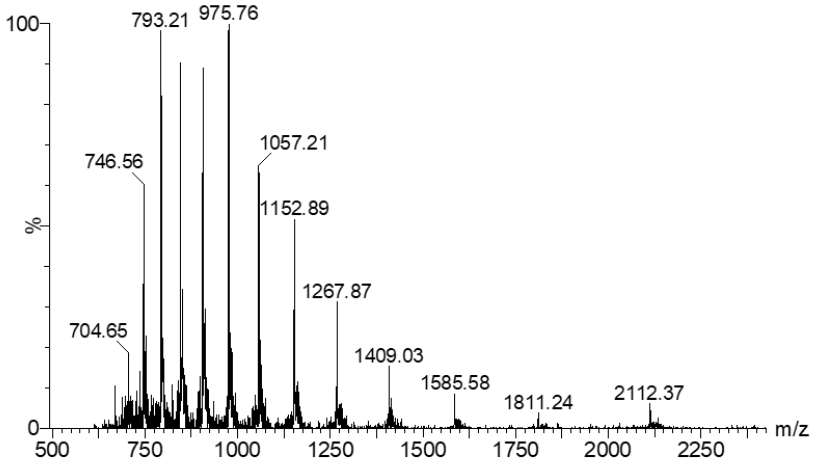

Figure S29. UV trace from analytical RP-UPLC and its ESI-MS of ***9***. Gradient: 5-95% ACN/H_2O containing 0.1% TFA over 5 min at a flow rate of 0.4 mL/min. ESI-MS calculated for $C_{575}H_{882}N_{152}O_{154}S_9$ $[M+6H]^{6+}$ m/z = 2113.80, found 2112.37, $[M+7H]^{7+}$ m/z = 1811.98, found 1811.24, $[M+8H]^{8+}$ m/z = 1585.60, found 1585.58, $[M+9H]^{9+}$ m/z = 1409.54, found 1409.03, $[M+10H]^{10+}$ m/z = 1268.68, found 1267.87, $[M+11H]^{11+}$ m/z = 1153.44, found 1152.89, $[M+12H]^{12+}$ m/z = 1057.40, found 1057.21, $[M+13H]^{13+}$ m/z = 976.14, found 975.76, $[M+14H]^{14+}$ m/z = 906.49, found 906.24, $[M+15H]^{15+}$ m/z = 846.12, found 846.04 , $[M+16H]^{16+}$ m/z = 793.30, found 793.21 , $[M+17H]^{17+}$ m/z = 746.70, found 746.56 , $[M+18H]^{18+}$ m/z = 705.27, found 704.65.

2.4 Synthesis of *17*

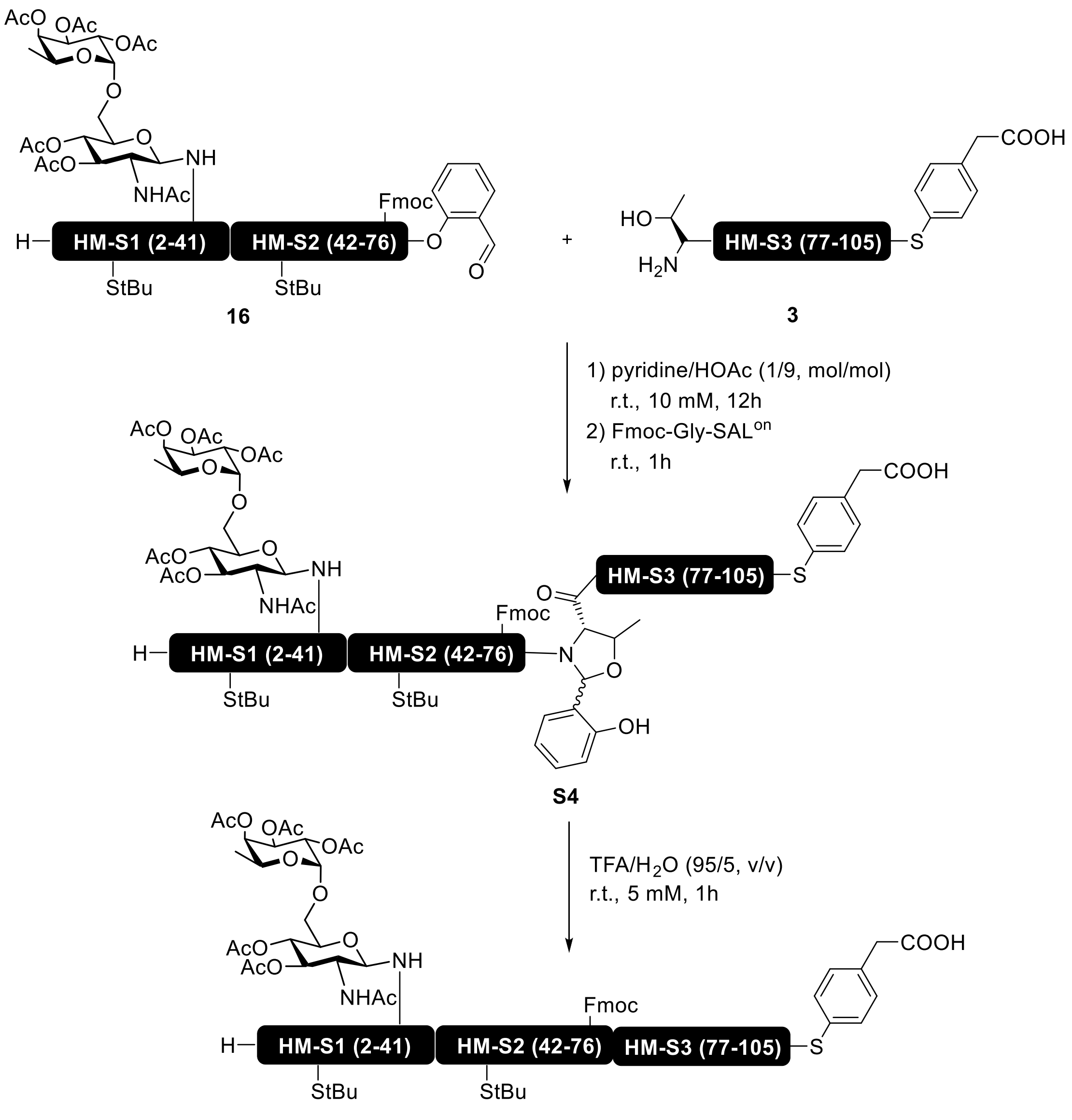

Figure S30. Synthetic route of ***17.***

Peptide salicylaldehyde ester ***16*** (19.0 mg, 1.9 μmol) and peptide MPAA ester ***3*** (8.8 mg, 2.5 μmol) were dissolved in pyridine/HOAc (1/9, mol/mol) at a concentration of 10 mM at room temperature. The reaction mixture was stirred at room temperature for 13h to afford ***S3***. After that, 10 equiv. Fmoc-Gly-SAL^{on} was dissolved in same amount of ligation buffer and added to the reaction for 1h to consume the unreacted ***3***. Then, the solvent was removed under condensed air and washed with diethyl ether and centrifuged. The residue was treated with TFA/H_2O (95/5, v/v) at a concentration of 5 mM for 1h to afford ***17***. Then, TFA was blown off and the reside was washed with diethyl ether and centrifuged. The crude ligation product was purified by preparative reverse-phase HPLC (20 to 70% ACN/H_2O over 45 min, 0.1% TFA). After lyophilization, 7.0 mg ***17*** was obtained as a white solid in an isolated yield of 27.4%.

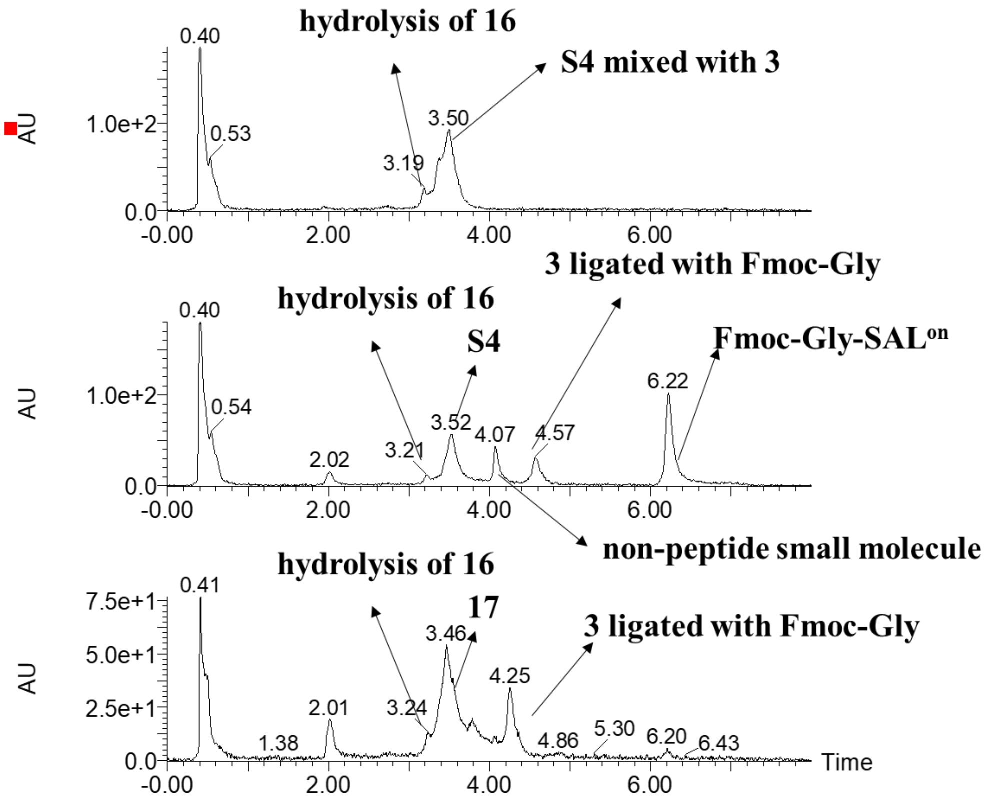

Figure S31. UV trace from analytical RP-UPLC of threonine ligation reaction mixture between ***16*** and ***3***. (A) Serine ligation at 13h. Gradient: 20-70% ACN/H_2O containing 0.1% TFA over 8 min at a flow rate of 0.4 mL/min. (B) Adding Fmoc-Gly-SAL^{on} at 1h. Gradient: 20-70% ACN/H_2O containing 0.1% TFA over 8 min at a flow rate of 0.4 mL/min. (C) Acidolysis at 1h. Gradient: 20-70 % ACN/H_2O containing 0.1% TFA over 8 min at a flow rate of 0.4 mL/min.

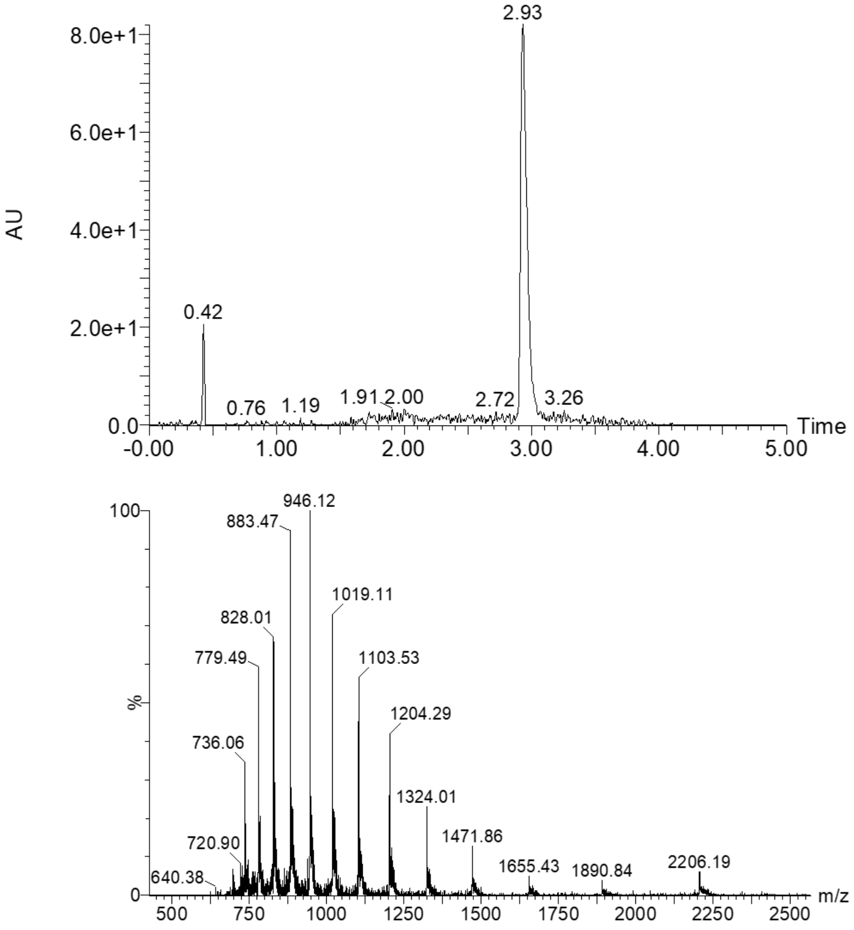

Figure S32. UV trace from analytical RP-UPLC and its ESI-MS of ***17***. Gradient: 5-95% ACN/H_2O containing 0.1% TFA over 5 min at a flow rate of 0.4 mL/min. ESI-MS calculated for $C_{599}H_{915}N_{153}O_{168}S_9$ $[M+6H]^{6+}$ m/z = 2207.06, found 2206.19, $[M+7H]^{7+}$ m/z = 1891.91, found 1890.84, $[M+8H]^{8+}$ m/z = 4655.54, found 1655.43, $[M+9H]^{9+}$ m/z = 1471.71, found 1471.86, $[M+10H]^{10+}$ m/z = 1324.64, found 1324.01, $[M+11H]^{11+}$ m/z = 1204.30, found 1204.29, $[M+12H]^{12+}$ m/z = 1104.03, found 1103.53, $[M+13H]^{13+}$ m/z = 1019.18, found 1019.11, $[M+14H]^{14+}$ m/z = 946.43, found 946.12, $[M+15H]^{15+}$ m/z = 883.42, found 883.47 , $[M+16H]^{16+}$ m/z = 828.27, found 828.01 , $[M+17H]^{17+}$ m/z = 779.61, found 779.49 , $[M+18H]^{18+}$ m/z = 736.35, found 736.06.

3. Synthesis of C-terminus part of HMGB1 and its variants

3.1 Synthesis of *10*

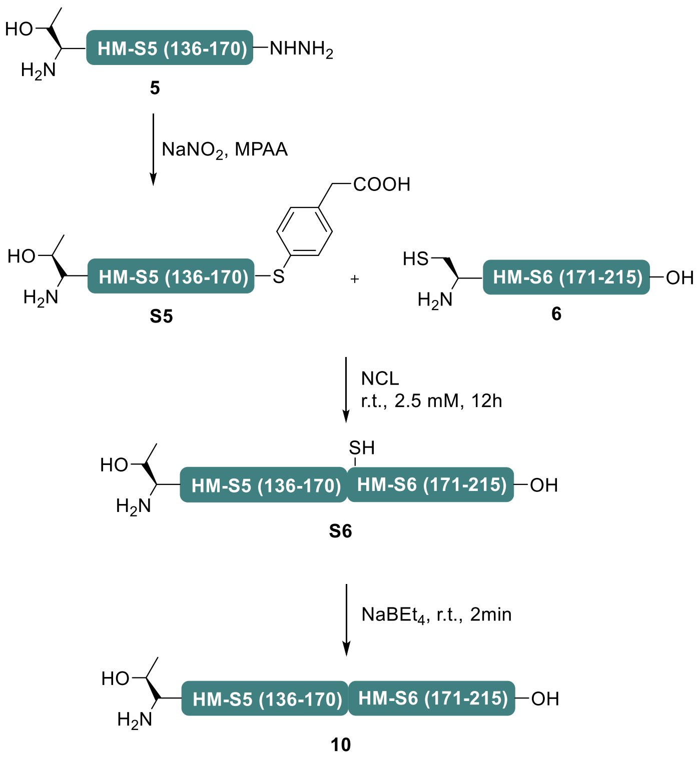

Figure S33. Synthetic route of ***10.***

Peptide hydrazide ***5*** (1 equiv.,132.0 mg, 33.4 μmol, 2.5 mM) was dissolved in aqueous buffer containing 6 M guanidine hydrochloride (Gn·HCl) and 0.2 M NaH_2PO_4 (pH 3.0) and cooled to approximately -15°C in an ice-salt bath. 0.5 M $NaNO_2$ (10 equiv.) was then added to activate the peptide hydrazide and stirred at -15°C for 15 min. After that, 4-mercaptophenylacetic acid (MPAA, 100 equiv., 250mM) was dissolved in 0.2 M NaH_2PO_4 solution containing 6 M Gn·HCl (pH 7.0), then this solution was added into the above mixture and stirred at room temperature for 30min to afford peptide MPAA ester ***S5***. After that, peptide carboxylic acid ***6*** (165.0 mg, 30.4 μmol) was added to the above buffer for 12h to afford ***S6***. The crude product was purified by preparative reverse-phase HPLC (10 to 50% ACN/H_2O over 45 min, 0.1% TFA) and then lyophilized as a white solid. Peptide carboxylic acid ***S6*** was dissolved in aqueous buffer containing 0.5 M sodium citrate, 6 M Gn·HCl and 0.1 M TCEP (pH 4.5). Then, 50 mM $NaBEt_4$ was added for 2min to afford ***10***. The crude product was purified by preparative reverse-phase HPLC (10 to 50% ACN/H_2O over 45 min, 0.1% TFA). After lyophilization, 86.5 mg ***10*** was obtained as a white solid in an isolated yield of 30.6% over 2 steps.

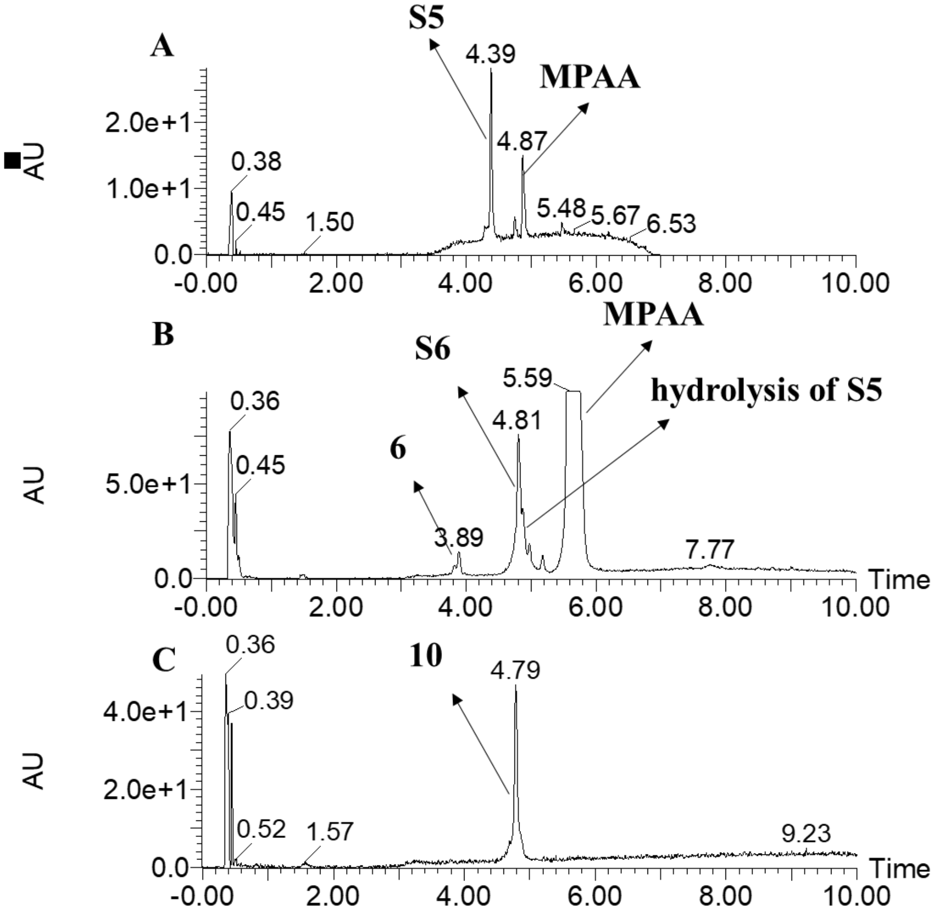

Figure S34. UV trace from analytical RP-UPLC of native chemical ligation reaction mixture between ***5*** and ***6***. (A) MPAA ester formation. Gradient: 5-95% ACN/H_2O containing 0.1% TFA over 7 min at a flow rate of 0.4 mL/min. (B) Native chemical ligation at 12h. Gradient: 5-10-60% ACN/H_2O containing 0.1% TFA over 10 min at a flow rate of 0.4 mL/min. (C) Desulfurization at 2min. Gradient: 5-10-60% ACN/H_2O containing 0.1% TFA over 10 min at a flow rate of 0.4 mL/min.

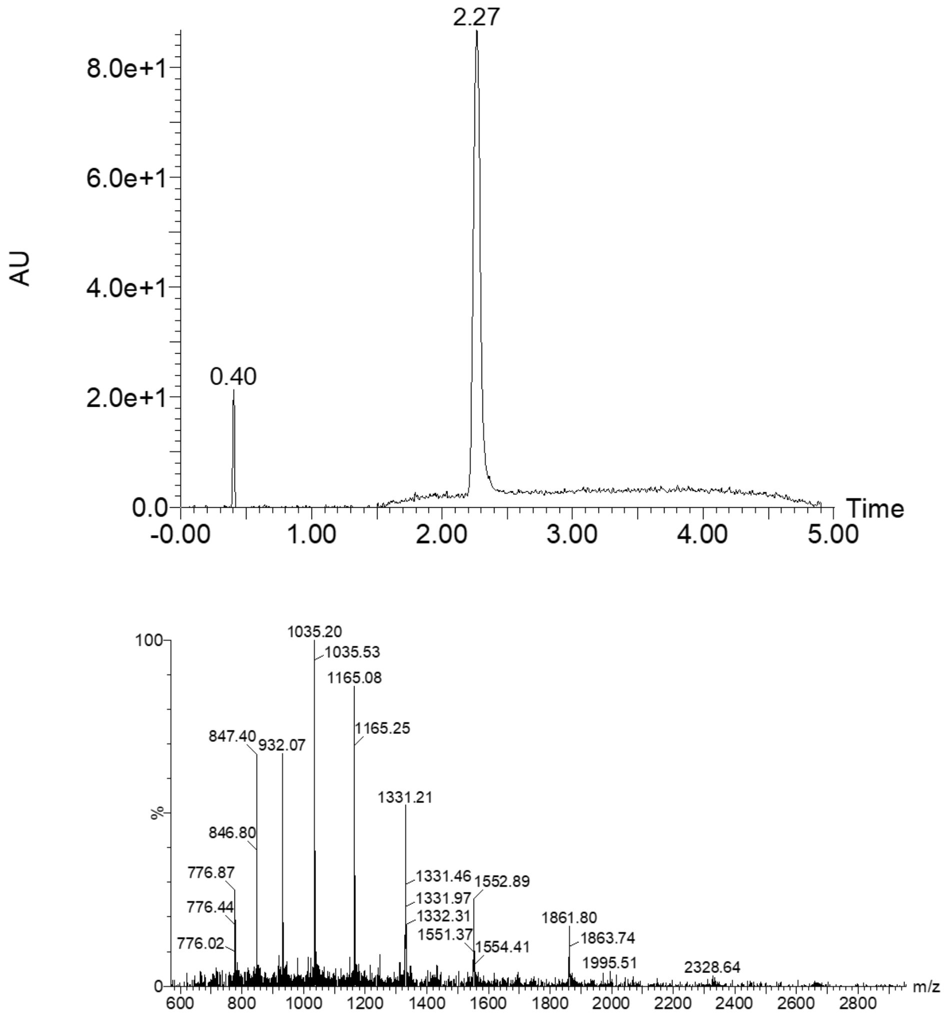

Figure S35. UV trace from analytical RP-UPLC and its ESI-MS of ***10***. Gradient: 5-95% ACN/H_2O containing 0.1% TFA over 5 min at a flow rate of 0.4 mL/min. ESI-MS calculated for $C_{389}H_{613}N_{101}O_{163}$ $[M+4H]^{4+}$ m/z = 2329.18, found 2328.64, $[M+5H]^{5+}$ m/z = 1863.55, found 1863.74, $[M+6H]^{6+}$ m/z = 1553.12, found 1552.89, $[M+7H]^{7+}$ m/z = 1331.39, found 1331.21, $[M+8H]^{8+}$ m/z = 1165.09, found 1165.08, $[M+9H]^{9+}$ m/z = 1035.75, found 1035.20, $[M+10H]^{10+}$ m/z = 932.27, found 932.07, $[M+11H]^{11+}$ m/z = 847.61, found 847.40, $[M+12H]^{12+}$ m/z = 777.06, found 776.87.

3.2 Synthesis of *11*

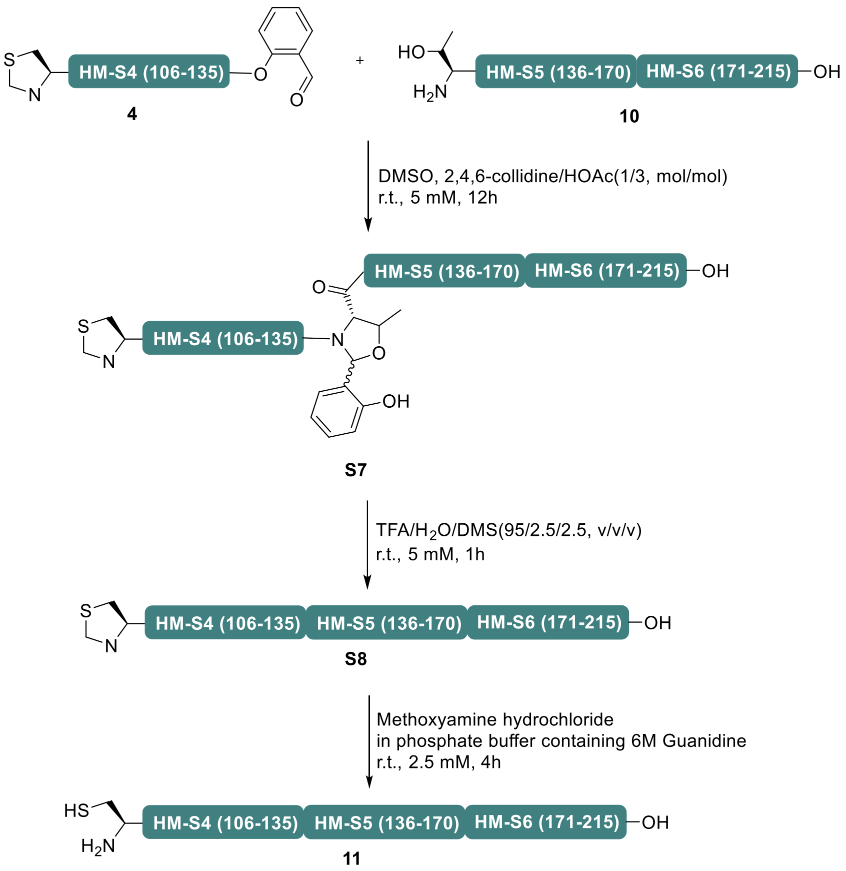

Figure S36. Synthetic route of ***11.***

Peptide salicylaldehyde ester ***4*** (16.0 mg, 4.6 μmol) and peptide carboxylic acid ***10*** (29.3 mg, 3.2 μmol) were dissolved in DMSO first, then 70% volume of 2,4,6-collidine/HOAc (1/3, mol/mol) at a concentration of 5 mM was added. The reaction mixture was stirred at room temperature for 12h to afford ***S7***. After that, the solvent was removed under condensed air and washed with diethyl ether and centrifuged. The residue was treated with TFA/H_2O (95/5, v/v) at a concentration of 5 mM for 1h to afford ***S8***. Then, TFA was blown off and the reside was washed with diethyl ether and centrifuged. The residue was dissolved in aqueous buffer containing 6 M Gn·HCl and 0.2 M NaH_2PO_4 (pH 3.0). 50 equiv. methoxyamine hydrochloride respective to ***4*** was added to the above solution and then adjust the pH value to around 4. The solution was stirred at room temperature for 4 hours to afford ***11***. The crude ligation product was purified by preparative reverse-phase HPLC (10 to 60% ACN/H_2O over 45 min, 0.1% TFA). After lyophilization, 10.0 mg ***11*** was obtained as a white solid in an isolated yield of 25.0%.

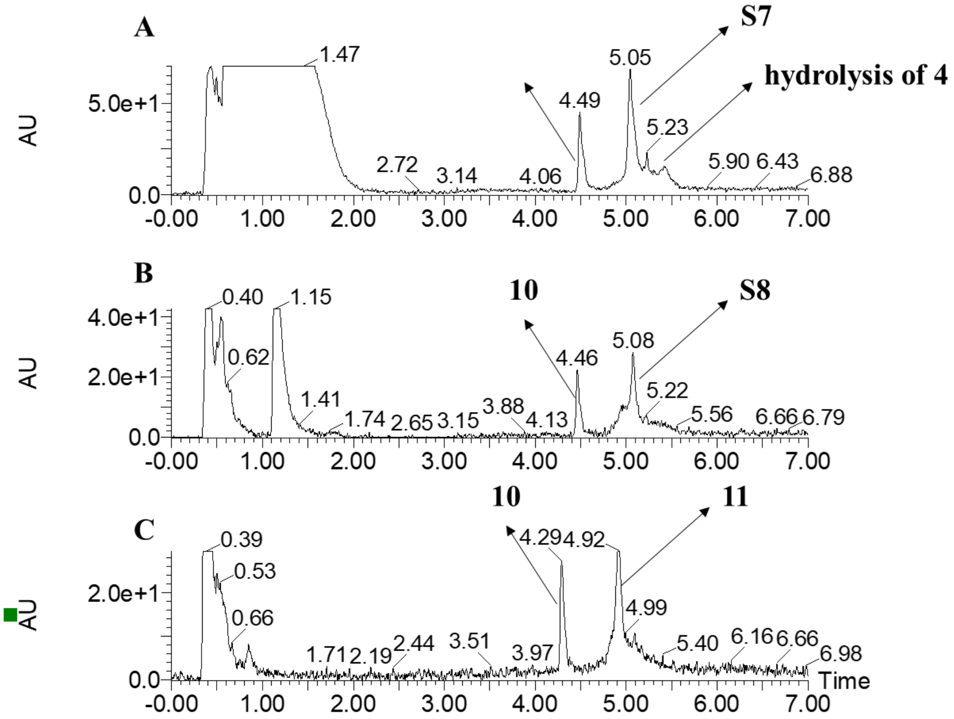

Figure S37. UV trace from analytical RP-UPLC of threonine ligation reaction mixture between ***4*** and ***10***. (A) Threonine ligation at 12h. Gradient: 5-10-60% ACN/H_2O containing 0.1% TFA over 7 min at a flow rate of 0.4 mL/min. (B) Acidolysis at 1h. Gradient: 5-10-60% ACN/H_2O containing 0.1% TFA over 7 min at a flow rate of 0.4 mL/min. (C) Thz opening at 4h. Gradient: 5-10-60 % ACN/H_2O containing 0.1% TFA over 7 min at a flow rate of 0.4 mL/min.

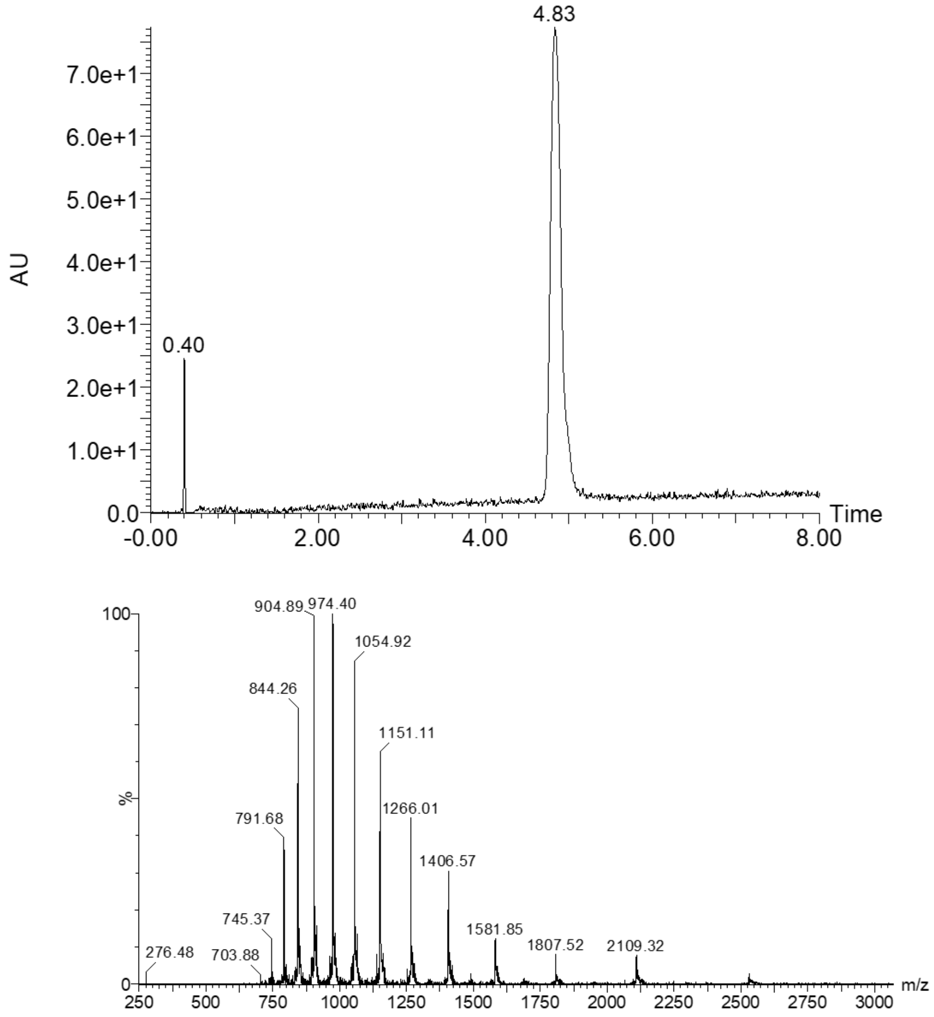

Figure S38. UV trace from analytical RP-UPLC and its ESI-MS of ***11***. Gradient: 10-50% ACN/H_2O containing 0.1% TFA over 8 min at a flow rate of 0.4 mL/min. ESI-MS calculated for $C_{536}H_{845}N_{143}O_{206}S_2$ $[M+6H]^{6+}$ m/z = 2109.76, found 2109.32, $[M+7H]^{7+}$ m/z = 1808.51, found 1807.52, $[M+8H]^{8+}$ m/z = 1582.57, found 1581.85, $[M+9H]^{9+}$ m/z = 1406.84, found 1406.57, $[M+10H]^{10+}$ m/z = 1266.26, found 1266.01, $[M+11H]^{11+}$ m/z = 1151.23, found 1151.11, $[M+12H]^{12+}$ m/z = 1055.38, found 1054.92, $[M+13H]^{13+}$ m/z = 974.27, found 974.40, $[M+14H]^{14+}$ m/z = 904.76, found 904.40, $[M+15H]^{15+}$ m/z = 844.50, found 844.26 , $[M+16H]^{16+}$ m/z = 791.79, found 791.68 , $[M+17H]^{17+}$ m/z = 745.27, found 745.37 , $[M+18H]^{18+}$ m/z = 703.92, found 703.88.

3.3 Synthesis of *19*

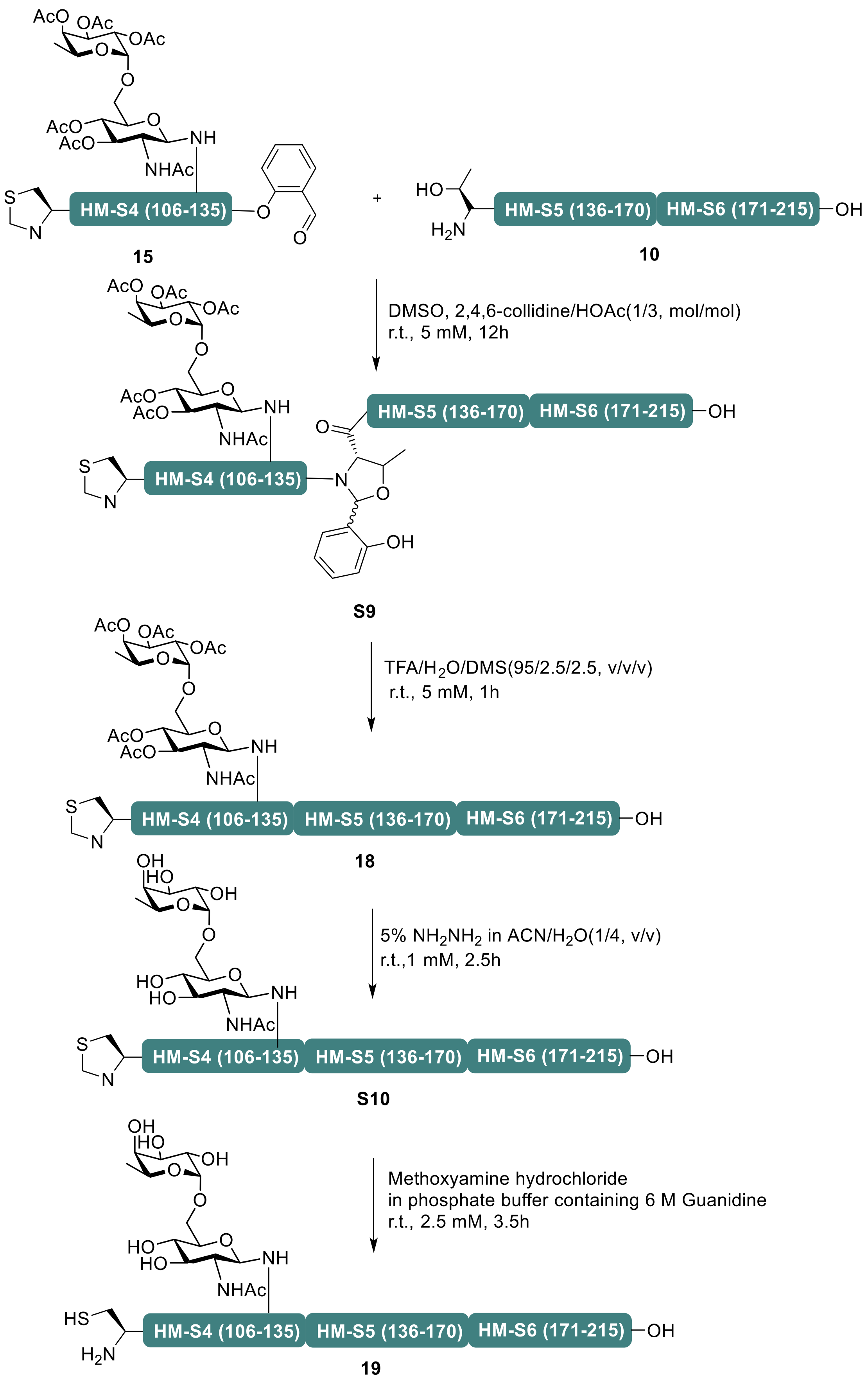
AcO OAc OAc
O
AcO
AcO
NH
NHAc
HM-S4 (106-135)
S
N
15
+
HO
H2N
HM-S5 (136-170)
HM-S6 (171-215)
OH
10
DMSO, 2,4,6-collidine/HOAc(1/3, mol/mol)
r.t., 5 mM, 12h
S9
TFA/H2O/DMS(95/2.5/2.5, v/v/v)
r.t., 5 mM, 1h
18
5% NH2NH2 in ACN/H2O(1/4, v/v)
r.t.,1 mM, 2.5h
S10
Methoxyamine hydrochloride
in phosphate buffer containing 6 M Guanidine
r.t., 2.5 mM, 3.5h
HS
19

Figure S39. Synthetic route of ***19.***

Peptide salicylaldehyde ester ***15*** (29.7 mg, 7.4 μmol) and peptide carboxylic acid ***10*** (51.5 mg, 5.5 μmol) were dissolved in DMSO first, then 70% volume of 2,4,6-collidine/acetic acid (1/3, mol/mol) at a concentration of 5 mM was added. The reaction mixture was stirred at room temperature for 12h to afford ***S9***. After that, Then, the solvent was removed under condensed air and washed with diethyl ether and centrifuged. The residue was treated with TFA/H_2O (95/5, v/v) at a concentration of 5 mM for 1h to afford ***18***. Then, TFA was blown off and the reside was washed with diethyl ether and centrifuged. The residue was dissolved in 20% ACN/H_2O and then 5% NH_2NH_2 (v/v) was added to the solution then stirred at room temperature for 2.5h to afford ***S10***. The crude ligation product was purified by preparative reverse-phase HPLC (10 to 60% ACN/H_2O over 45 min, 0.1% TFA). After lyophilization, ***S10*** was dissolved in aqueous buffer containing 6 M Gn·HCl and 0.2 M NaH_2PO_4 (pH 3.0). 50 equiv. methoxyamine hydrochloride was added to the above solution and then adjust the pH value to around 4. The solution was stirred at room temperature for 4h to afford ***19***. The crude product was purified by preparative reverse-phase HPLC (10 to 60% ACN/H_2O over 45 min, 0.1% TFA). After lyophilization, 15.0 mg ***19*** was obtained as a white solid in an isolated yield of 20.9% over 4 steps.

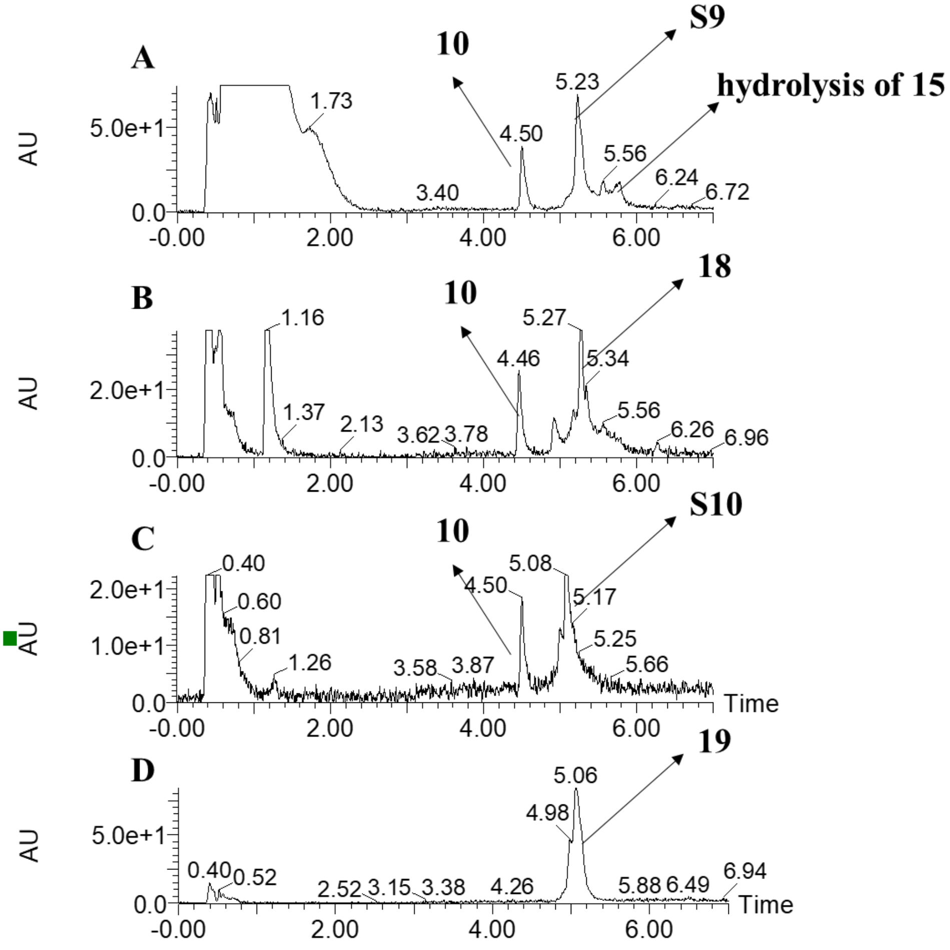

Figure S40. UV trace from analytical RP-UPLC of threonine ligation reaction mixture between ***15*** and ***10***. (A) Threonine ligation at 12h. Gradient: 5-10-60% ACN/H_2O containing 0.1% TFA over 7 min at a flow rate of 0.4 mL/min. (B) Acidolysis at 1h. Gradient: 5-10-60% ACN/H_2O containing 0.1% TFA over 7 min at a flow rate of 0.4 mL/min. (C) Ac removal at 2h30min. Gradient: 5-10-60 % ACN/H_2O containing 0.1% TFA over 7 min at a flow rate of 0.4 mL/min. (D) Thz opening at 4h. Gradient: 5-10-60 % ACN/H_2O containing 0.1% TFA over 7 min at a flow rate of 0.4 mL/min.

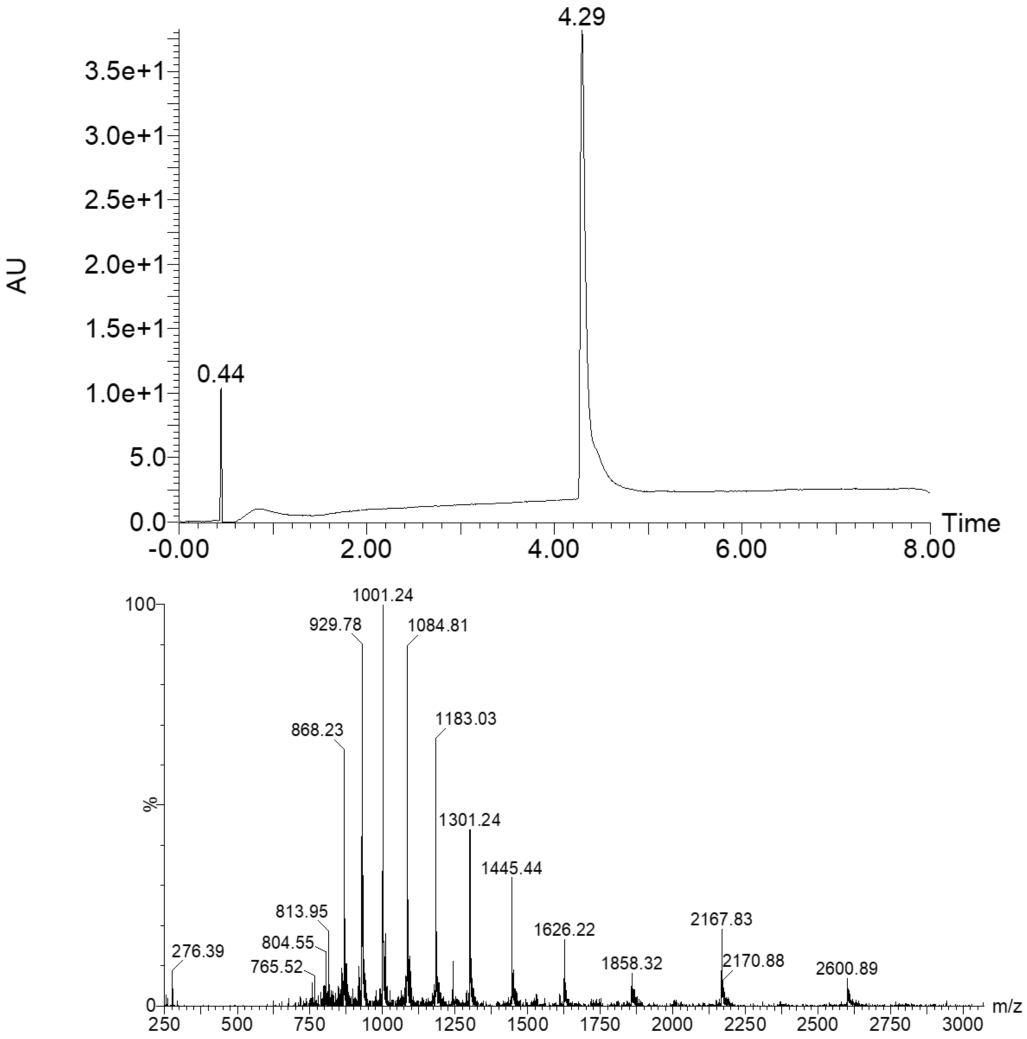

Figure S41. UV trace from analytical RP-UPLC and its ESI-MS of ***19***. Gradient: 10-50% ACN/H_2O containing 0.1% TFA over 8 min at a flow rate of 0.4 mL/min. ESI-MS calculated for $C_{550}H_{868}N_{144}O_{215}S_2$ $[M+5H]^{5+}$ m/z = 2601.38, found 2600.89, $[M+6H]^{6+}$ m/z = 2167.99, found 2167.83, $[M+7H]^{7+}$ m/z = 1858.42, found 1858.32, $[M+8H]^{8+}$ m/z = 1626.24, found 1626.22, $[M+9H]^{9+}$ m/z = 1445.66, found 1445.44, $[M+10H]^{10+}$ m/z = 1301.19, found 1301.24, $[M+11H]^{11+}$ m/z = 1182.99, found 1183.03, $[M+12H]^{12+}$ m/z = 1084.49, found 1084.81, $[M+13H]^{13+}$ m/z = 1001.15, found 1001.24, $[M+14H]^{14+}$ m/z = 929.71, found 929.78, $[M+15H]^{15+}$ m/z = 867.79, found 868.23 , $[M+16H]^{16+}$ m/z = 813.62, found 813.95.

3.4 Synthesis of *22*

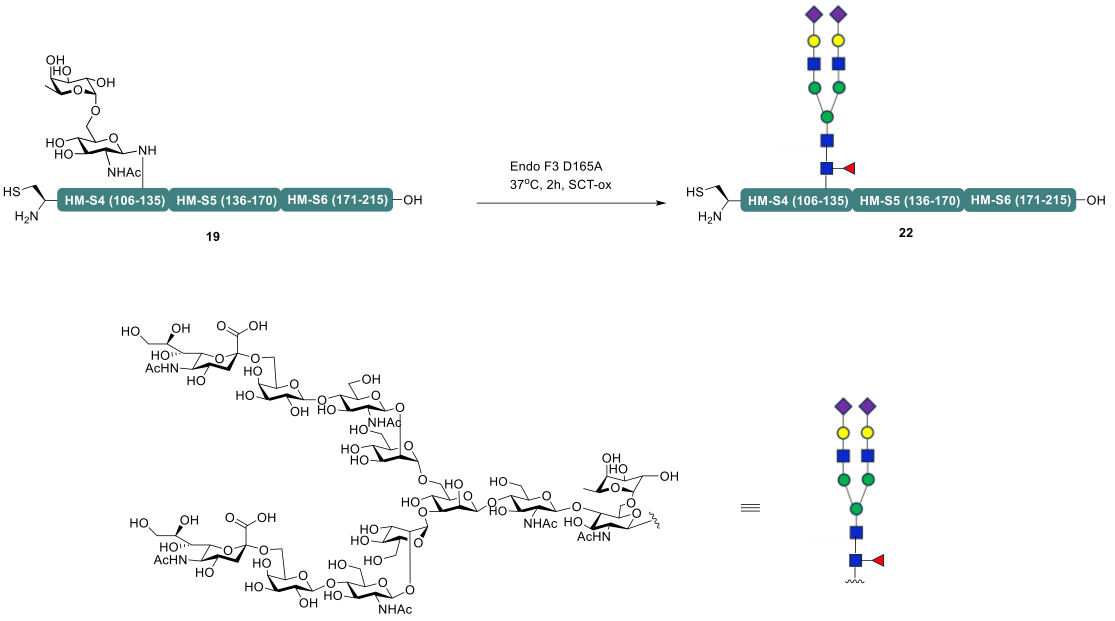

Figure S42. Synthetic route of ***22.***

Peptide carboxylic acid ***19*** (8.7 mg, 0.67 μmol) was dissolved in 10x PBS (pH=6.8). Then 125 μL Endo F3 D165A (4 μg/μL in 1x PBS) was added to the solution and mixed well. 10 equiv. SCTox was dissolved in 10x PBS and then added to the solution and mixed well. The mentioned solution was incubated at 37°C for 2h and monitored by UPLC-MS system. The crude product was purified by preparative reverse-phase HPLC (10 to 60% ACN/H_2O over 45 min, 0.1% TFA). After lyophilization, 3.5 mg ***22*** was obtained as a white solid in an isolated yield of 34.5%.

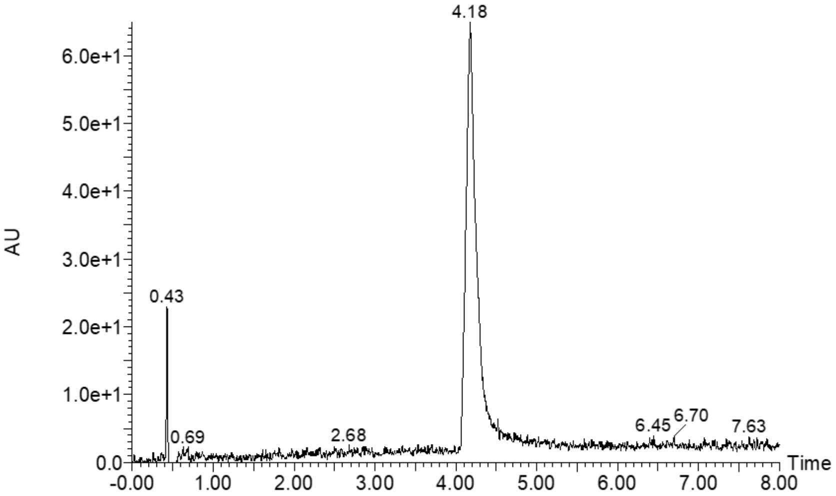

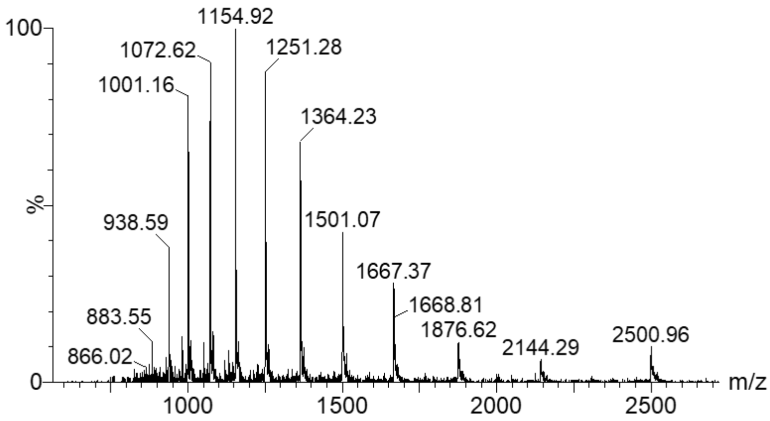

Figure S43. UV trace from analytical RP-UPLC and its ESI-MS of ***22***. Gradient: 10-60% ACN/H_2O containing 0.1% TFA over 5 min at a flow rate of 0.4 mL/min. ESI-MS calculated for $C_{626}H_{991}N_{149}O_{271}S_2$ $[M+6H]^{6+}$ m/z = 2501.79, found 2500.96, $[M+7H]^{7+}$ m/z = 2144.53, found 2144.29, $[M+8H]^{8+}$ m/z = 1876.59, found 1876.62, $[M+9H]^{9+}$ m/z = 1668.19, found 1667.37, $[M+10H]^{10+}$ m/z = 1501.47, found 1501.07, $[M+11H]^{11+}$ m/z = 1365.07, found 1364.23, $[M+12H]^{12+}$ m/z = 1251.39, found 1251.28, $[M+13H]^{13+}$ m/z = 1155.21, found 1154.92, $[M+14H]^{14+}$ m/z = 1072.77, found 1072.62, $[M+15H]^{15+}$ m/z = 1001.31, found 1001.16 , $[M+16H]^{16+}$ m/z = 938.79, found 938.59.

4. Assembly of N and C-terminus part of HMGB1 and its variants into linear proteins

4.1 Synthesis of *12*

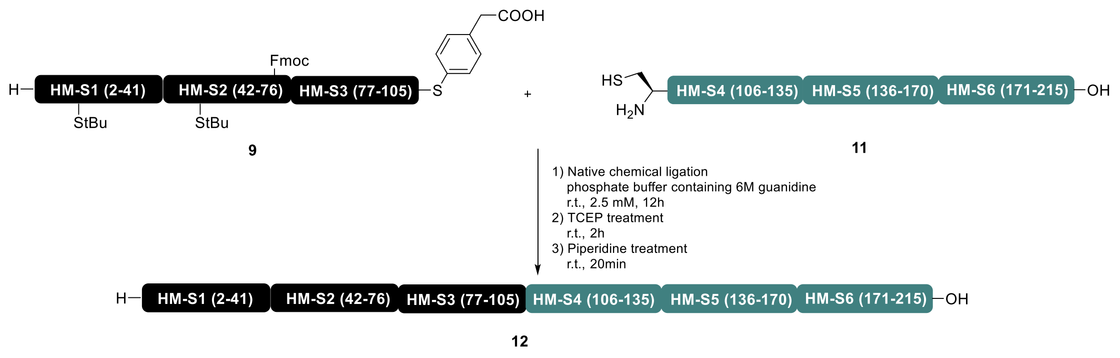

Figure S44. Synthetic route of ***12.***

Peptide MPAA ester ***9*** (3.1 mg, 0.25 μmol) and peptide carboxylic acid ***11*** (3.2 mg, 0.25 μmol) were dissolved in aqueous buffer containing 6 M Gn·HCl, 0.2 M NaH_2PO_4 and 100 mM MPAA (pH 7.0) at a concentration of 2.5 mM. The reaction was stirred at room temperature for 12h. Then, double volume of 0.1 M TCEP was added to the reaction solution for 2h to remove the S*t*Bu protection on cysteine and reduce the disulfide bond formed with MPAA during the reaction. After that, 20% piperidine (v/v) was added to the reaction solution for 20min to remove the Fmoc protection on lysine to afford ***12***. The crude product was purified by preparative reverse-phase HPLC (10 to 60% ACN/H_2O over 45 min, 0.1% TFA). After lyophilization, 1.9 mg ***12*** was obtained as a white solid in an isolated yield of 31.3% over 3 steps.

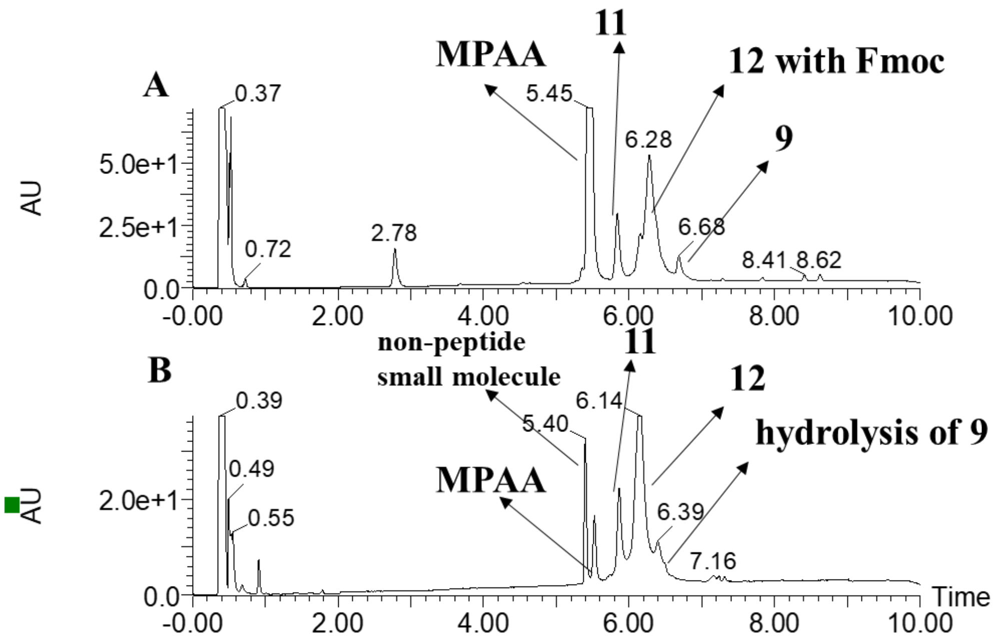

Figure S45. UV trace from analytical RP-UPLC of native chemical ligation reaction mixture between ***9*** and ***11***. (A) native chemical ligation at 12h after TCEP treatment. Gradient: 5-10-60% ACN/H_2O containing 0.1% TFA over 10 min at a flow rate of 0.4 mL/min. (B) Piperidine treatment at 20min. Gradient: 5-10-60% ACN/H_2O containing 0.1% TFA over 10 min at a flow rate of 0.4

mL/min.

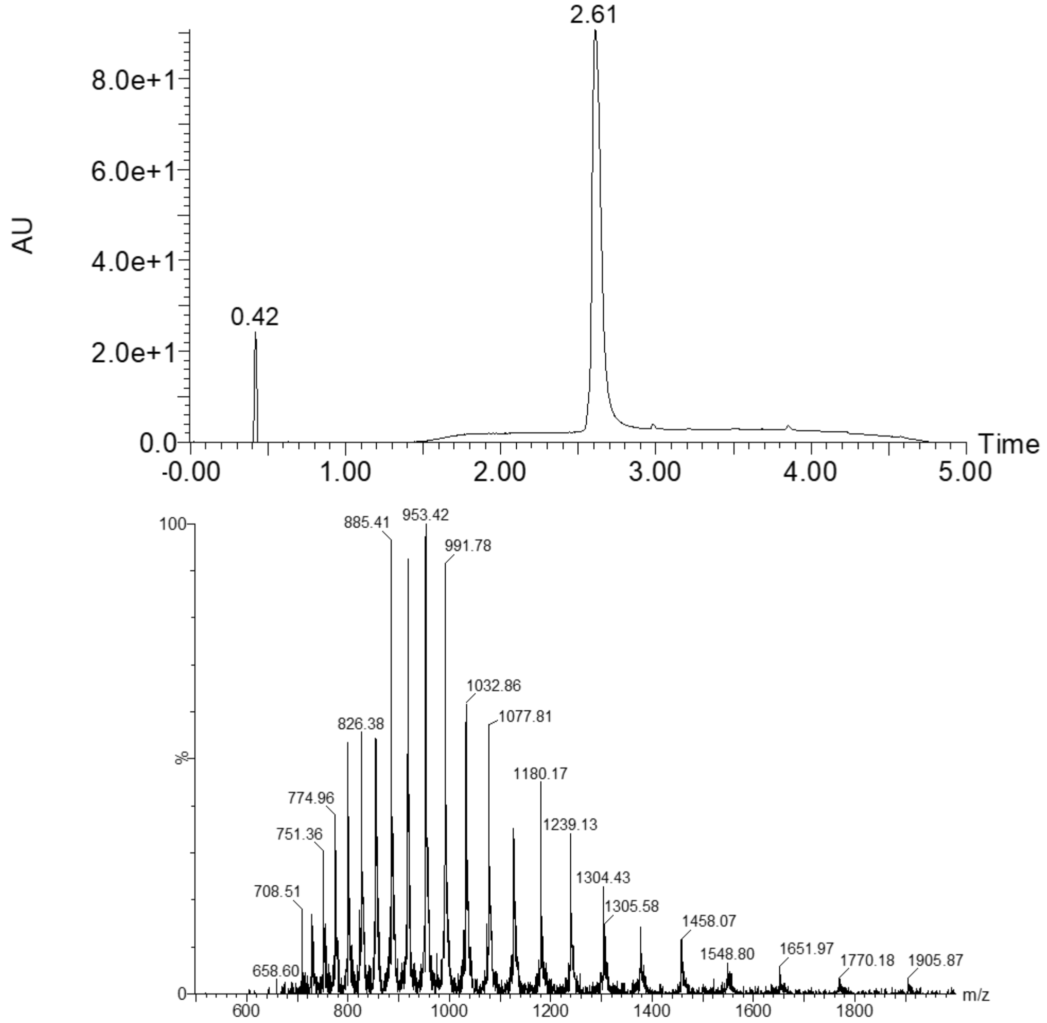

Figure S46. UV trace from analytical RP-UPLC and its ESI-MS of ***12***. Gradient: 5-95% ACN/H_2O containing 0.1% TFA over 5 min at a flow rate of 0.4 mL/min. ESI-MS calculated for $C_{1080}H_{1693}N_{295}O_{356}S_8$ $[M+13H]^{13+}$ m/z = 1905.82, found 1905.87, $[M+14H]^{14+}$ m/z = 1769.76, found 1770.18, $[M+15H]^{15+}$ m/z = 1651.84, found 1651.97, $[M+16H]^{16+}$ m/z = 1548.66, found 1548.80, $[M+17H]^{17+}$ m/z = 1457.62, found 1458.07, $[M+18H]^{18+}$ m/z = 1376.70, found 1377.00, $[M+19H]^{19+}$ m/z = 1304.30, found 1304.43, $[M+20H]^{20+}$ m/z = 1239.13, found 1239.13, $[M+21H]^{21+}$ m/z = 1180.17, found 1180.17, $[M+22H]^{22+}$ m/z = 1126.57, found 1126.71, $[M+23H]^{23+}$ m/z = 1077.64, found 1077.81, $[M+24H]^{24+}$ m/z = 1032.78, found 1032.86, $[M+25H]^{25+}$ m/z = 991.50, found 991.78, $[M+26H]^{26+}$ m/z = 953.41, found 953.42, $[M+27H]^{27+}$ m/z = 918.13, found 918.19, $[M+28H]^{28+}$ m/z = 885.38, found 885.41, $[M+29H]^{29+}$ m/z = 854.88, found 854.87, $[M+30H]^{30+}$ m/z = 826.42, found 826.38, $[M+31H]^{31+}$ m/z = 799.79, found 799.92, $[M+32H]^{32+}$ m/z = 774.83, found 774.96, $[M+33H]^{33+}$ m/z = 751.38, found 751.36, $[M+34H]^{34+}$ m/z = 729.31,

found 729.05, $[M+35H]^{35+}$ m/z = 708.50, found 708.51.

4.2 Synthesis of *20a*

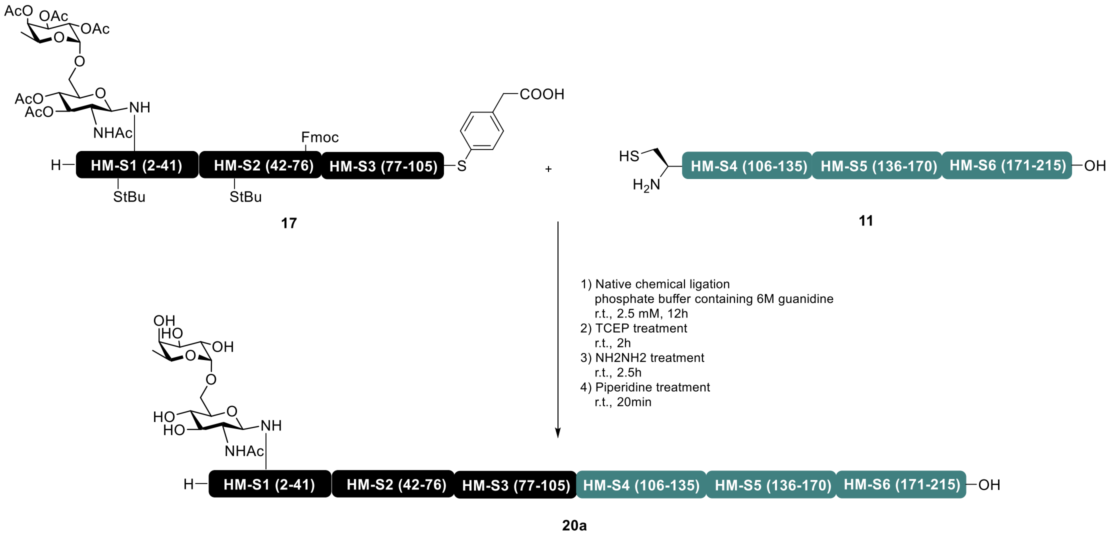

Figure S47. Synthetic route of ***20a.***

Peptide MPAA ester ***17*** (4.3 mg, 0.33 μmol) and peptide carboxylic acid ***11*** (5.0 mg, 0.40 μmol) dissolved in aqueous buffer containing 6 M Gn·HCl, 0.2 M NaH_2PO_4 and 100 mM MPAA (pH 7.0) at a concentration of 2.5 mM. The reaction was stirred at room temperature for 12h. Then, double volume of 0.1 M TCEP was added to the reaction solution for 2h to remove the S*t*Bu protection on cysteine and reduce the disulfide bond formed with MPAA during the reaction. Then 5% (v/v) of NH_2NH_2 was added to the reaction solution for 2.5h to remove the *O*-acetyl protection on the disaccharide hydroxyl groups. After that, 20% (v/v) of piperidine was added to the reaction solution for 20min to remove the Fmoc protection on lysine to afford ***20a***. The crude product was purified by preparative reverse-phase HPLC (10 to 60% ACN/H_2O over 45 min, 0.1% TFA). After lyophilization, 3.0 mg ***20a*** was obtained as a white solid in an isolated yield of 36.6% over 4 steps.

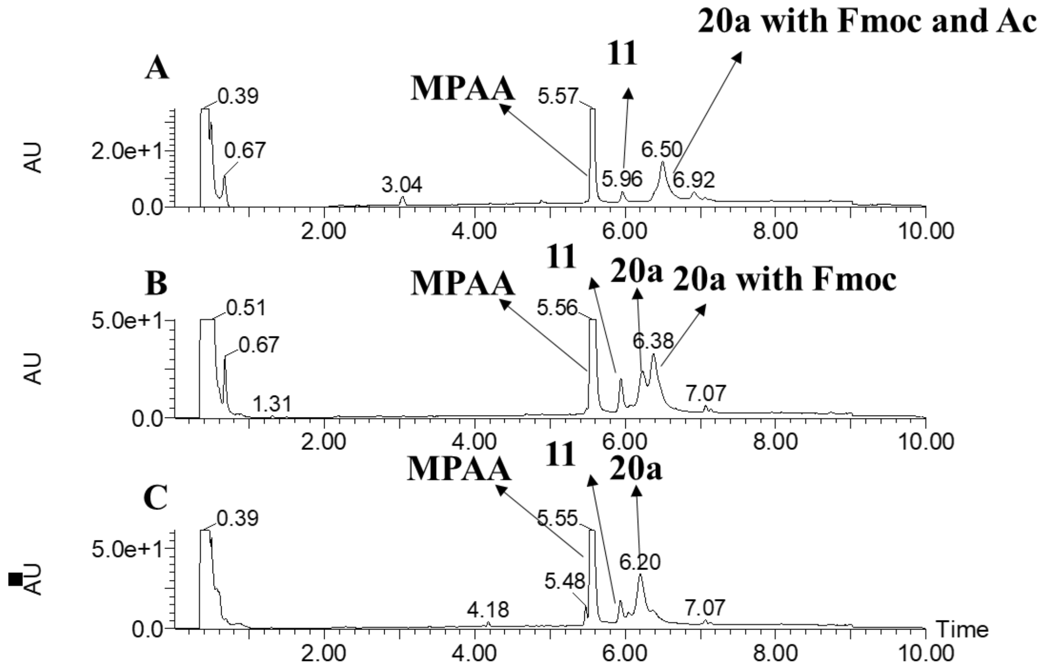

Figure S48. UV trace from analytical RP-UPLC of native chemical ligation reaction mixture between ***17*** and ***11***. **(A)** native chemical ligation at 12h after TCEP treatment. Gradient: 5-10-60% ACN/H_2O containing 0.1% TFA over 10 min at a flow rate of 0.4 mL/min. **(B)** Hydrazine treatment at 2h30min**.** Piperidine treatment at 20min. Gradient: 5-10-60% ACN/H_2O containing 0.1% TFA over 10 min at a flow rate of 0.4 mL/min. **(C)** Piperidine treatment at 20min. Gradient: 5-10-60% ACN/H_2O containing 0.1% TFA over 10 min at a flow rate of 0.4 mL/min.

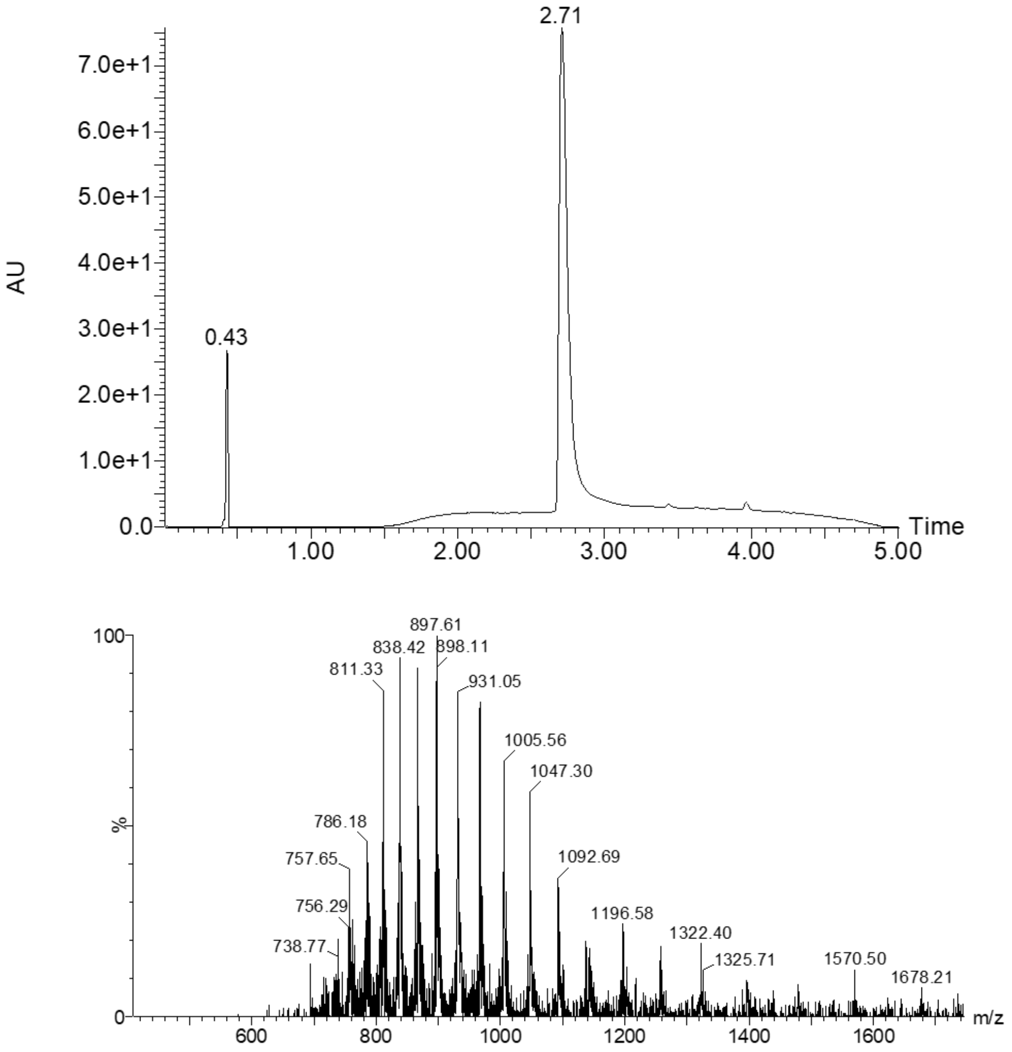

Figure S49. UV trace from analytical RP-UPLC and its ESI-MS of ***20a***. Gradient: 5-95% ACN/H_2O containing 0.1% TFA over 5 min at a flow rate of 0.4 mL/min. ESI-MS calculated for $C_{1094}H_{1716}N_{296}O_{365}S_8$ $[M+19H]^{19+}$ m/z = 1322.68, found 1322.40, $[M+20H]^{20+}$ m/z = 1256.60, found 1256.87, $[M+21H]^{21+}$ m/z = 1196.80, found 1196.58, $[M+22H]^{22+}$ m/z = 1142.45, found 1142.30, $[M+23H]^{23+}$ m/z = 1092.82, found 1092.69, $[M+24H]^{24+}$ m/z = 1047.33, found 1047.30, $[M+25H]^{25+}$ m/z = 1005.48, found 1005.56, $[M+26H]^{26+}$ m/z = 966.84, found 966.87, $[M+27H]^{27+}$ m/z = 931.07, found 931.05, $[M+28H]^{28+}$ m/z = 897.86, found 897.61, $[M+29H]^{29+}$ m/z = 866.93, found 867.38, $[M+30H]^{30+}$ m/z = 838.07, found 838.42, $[M+31H]^{31+}$ m/z = 811.06, found 811.33, $[M+32H]^{32+}$ m/z = 785.75, found 786.18.

4.3 Synthesis of *20b*

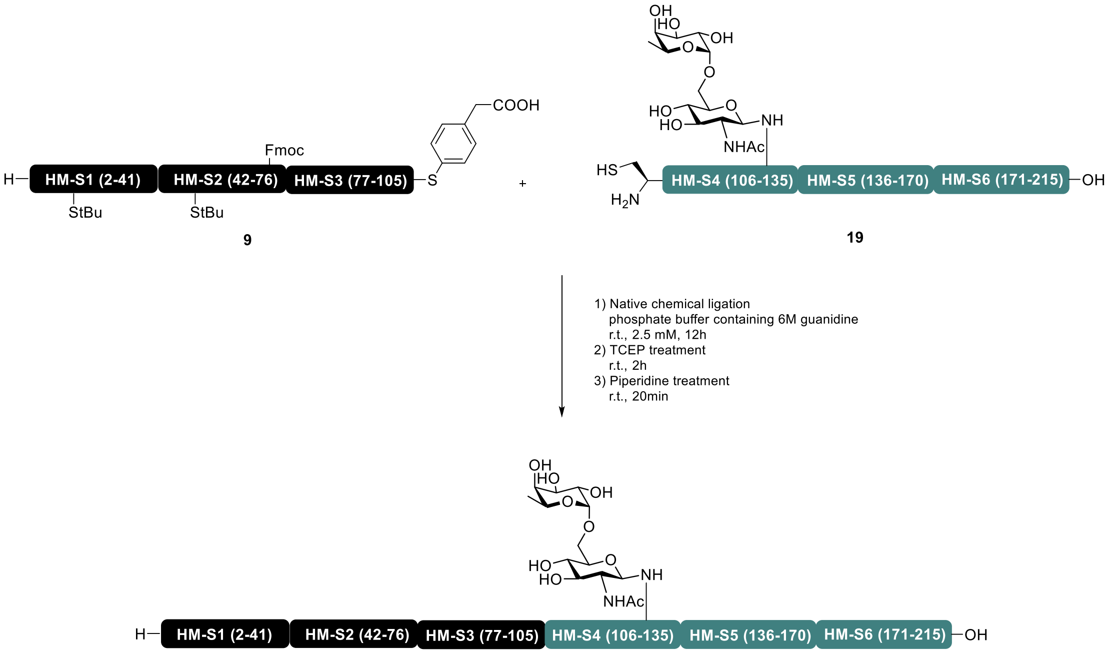

9

19

20b

Figure S50. Synthetic route of ***20b.***

Peptide MPAA ester ***9*** (2.9 mg, 0.23 µmol) and peptide carboxylic acid ***19*** (3.2 mg, 0.25 µmol) were dissolved in aqueous buffer containing 6 M Gn·HCl, 0.2 M NaH_2PO_4 and 100 mM MPAA (pH 7.0) at a concentration of 2.5 mM. The reaction was stirred at room temperature for 12h. Then, double volume of 0.1 M TCEP was added to the reaction solution for 2h to remove the S*t*Bu protection on cysteine and reduce the disulfide bond formed with MPAA during the reaction. After that, 20% piperidine (v/v) was added to the reaction solution for 20min to remove the Fmoc protection on lysine to afford ***20b***. The crude product was purified by preparative reverse-phase HPLC (10 to 60% ACN/H_2O over 45 min, 0.1% TFA). After lyophilization, 1.6 mg ***20b*** was obtained as a white solid in an isolated yield of 27.8% over 3 steps.

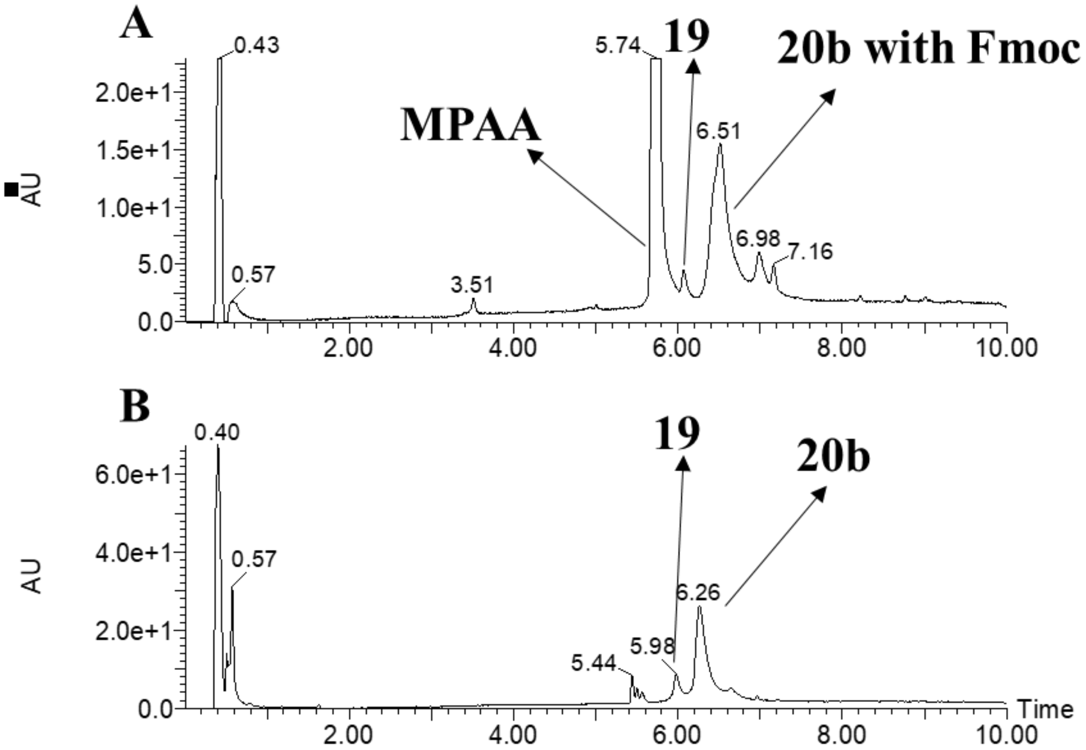

Figure S51. UV trace from analytical RP-UPLC of native chemical ligation reaction mixture between ***9*** and ***19***. **(A)** native chemical ligation at 12h after TCEP treatment. Gradient: 5-10-60% ACN/H_2O containing 0.1% TFA over 10 min at a flow rate of 0.4 mL/min. **(B)** Piperidine treatment at 20min. Gradient: 5-10-60% ACN/H_2O containing 0.1% TFA over 10 min at a flow rate of 0.4 mL/min.

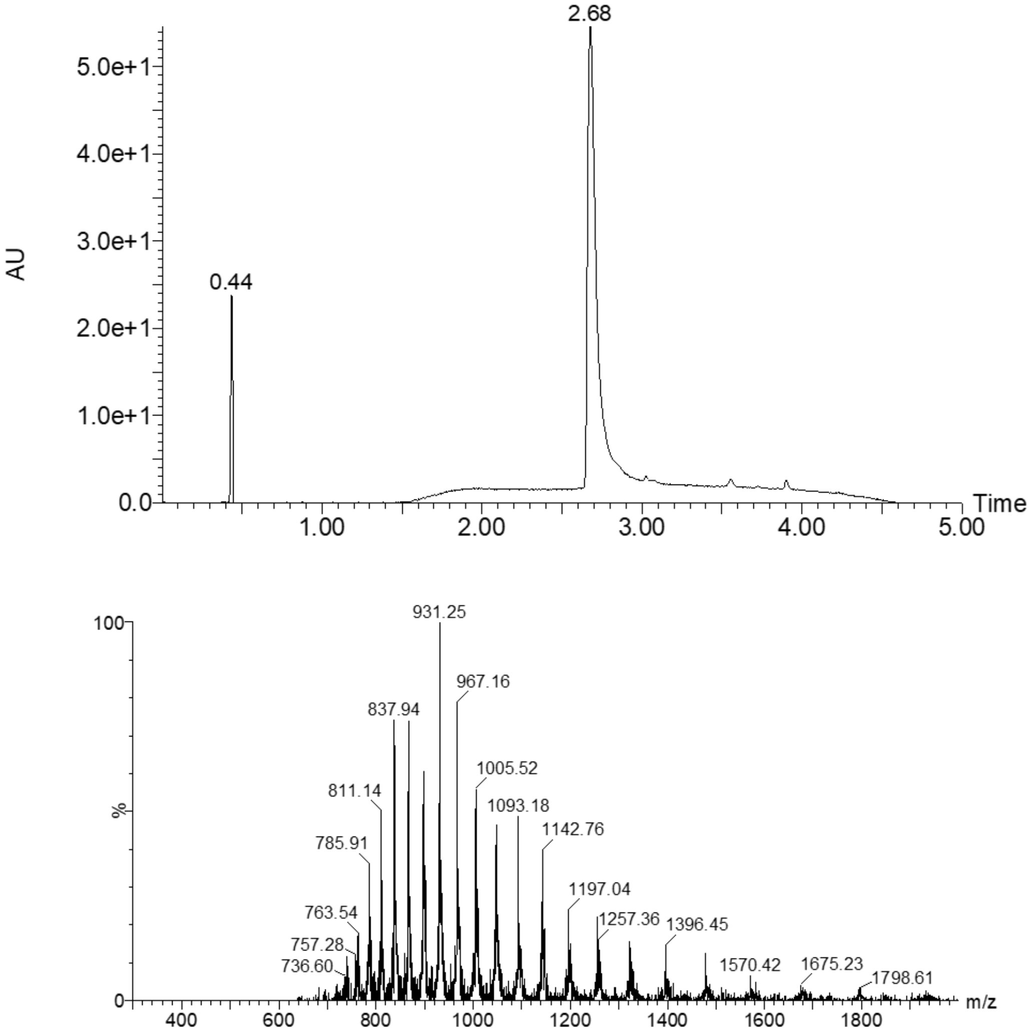

Figure S52. UV trace from analytical RP-UPLC and its ESI-MS of ***20b***. Gradient: 5-95% ACN/H_2O containing 0.1% TFA over 5 min at a flow rate of 0.4 mL/min. ESI-MS calculated for $C_{1094}H_{1716}N_{296}O_{365}S_8$ $[M+18H]^{18+}$ m/z = 1396.11, found 1396.45, $[M+19H]^{19+}$ m/z = 1322.68, found 1322.86, $[M+20H]^{20+}$ m/z = 1256.60, found 1257.36, $[M+21H]^{21+}$ m/z = 1196.80, found 1197.04, $[M+22H]^{22+}$ m/z = 1142.45, found 1142.76, $[M+23H]^{23+}$ m/z = 1092.82, found 1093.18, $[M+24H]^{24+}$ m/z = 1047.33, found 1047.55, $[M+25H]^{25+}$ m/z = 1005.48, found 1005.52, $[M+26H]^{26+}$ m/z = 966.84, found 967.16, $[M+27H]^{27+}$ m/z = 931.07, found 931.25, $[M+28H]^{28+}$ m/z = 897.86, found 897.92, $[M+29H]^{29+}$ m/z = 866.93, found 867.18, $[M+30H]^{30+}$ m/z = 838.07, found 837.94, $[M+31H]^{31+}$ m/z = 811.06, found 811.14, $[M+32H]^{32+}$ m/z = 785.75, found 785.91.

4.4 Synthesis of *20c*

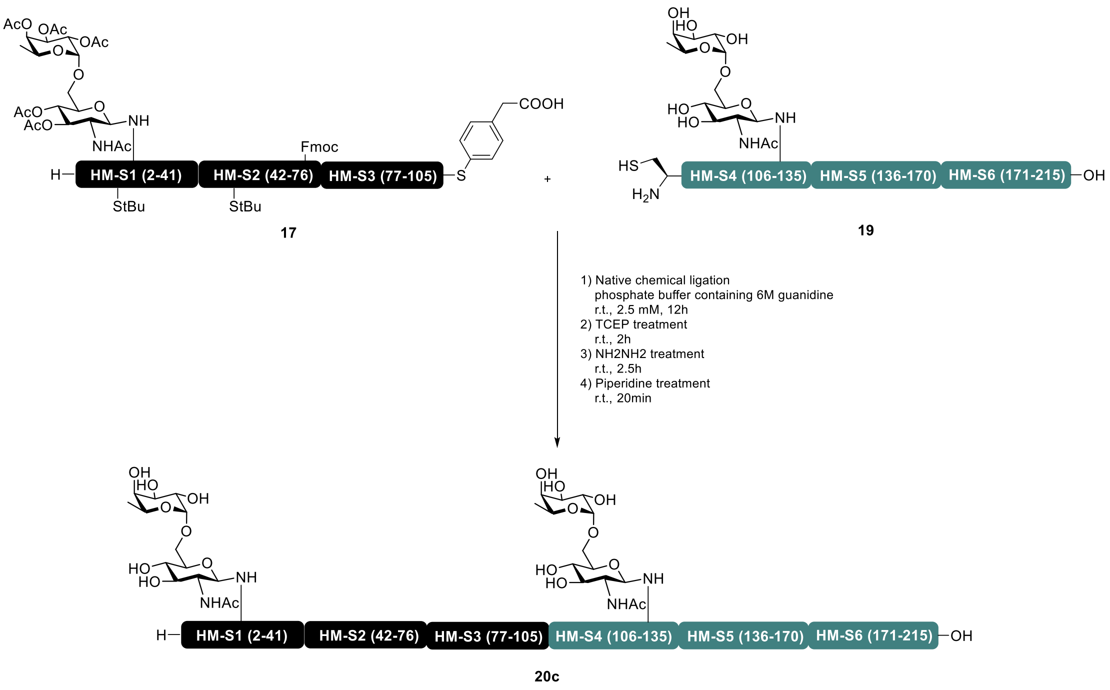

Figure S53. Synthetic route of ***20c.***

Peptide MPAA ester ***17*** (4.0 mg, 0.30 µmol) and peptide carboxylic acid ***19*** (5.0 mg, 0.39 µmol) dissolved in aqueous buffer containing 6 M Gn·HCl, 0.2 M NaH_2PO_4 and 100 mM MPAA (pH 7.0) at a concentration of 2.5 mM. The reaction was stirred at room temperature for 12h. Then, double volume of 0.1 M TCEP was added to the reaction solution for 2h to remove the S*t*Bu protection on cysteine and reduce the disulfide bond formed with MPAA during the reaction. Then 5% (v/v) of NH_2NH_2 was added to the reaction solution for 2.5h to remove the *O*-acetyl protection on the disaccharide hydroxyl groups. After that, 20% (v/v) of piperidine was added to the reaction solution for 20min to remove the Fmoc protection on lysine to afford ***20c***. The crude product was purified by preparative reverse-phase HPLC (10 to 60% ACN/H_2O over 45 min, 0.1% TFA). After lyophilization, 2.4 mg ***20c*** was obtained as a white solid in an isolated yield of 31.2% over 4 steps.

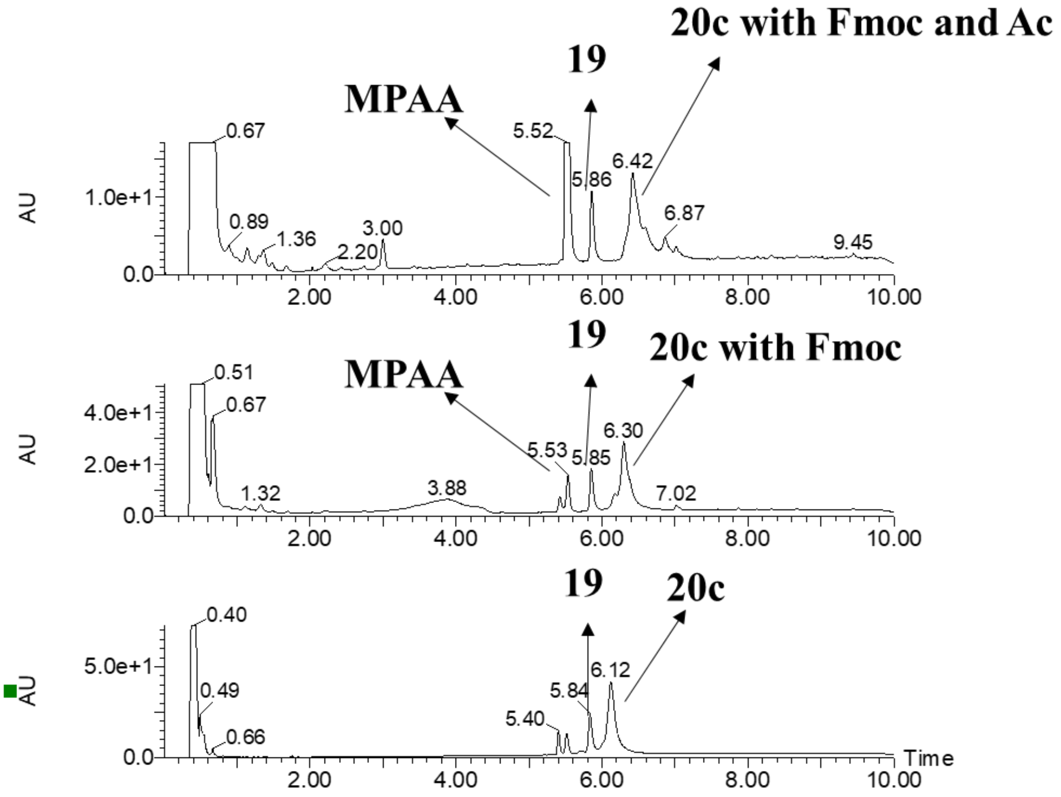

Figure S54. UV trace from analytical RP-UPLC of native chemical ligation reaction mixture between ***17*** and ***19***. **(A)** native chemical ligation at 12h after TCEP treatment. Gradient: 5-10-60% ACN/H_2O containing 0.1% TFA over 10 min at a flow rate of 0.4 mL/min. **(B)** Hydrazine treatment at 2h30min**.** Piperidine treatment at 20min. Gradient: 5-10-60% ACN/H_2O containing 0.1% TFA over 10 min at a flow rate of 0.4 mL/min. **(C)** Piperidine treatment at 20min. Gradient: 5-10-60% ACN/H_2O containing 0.1% TFA over 10 min at a flow rate of 0.4 mL/min.

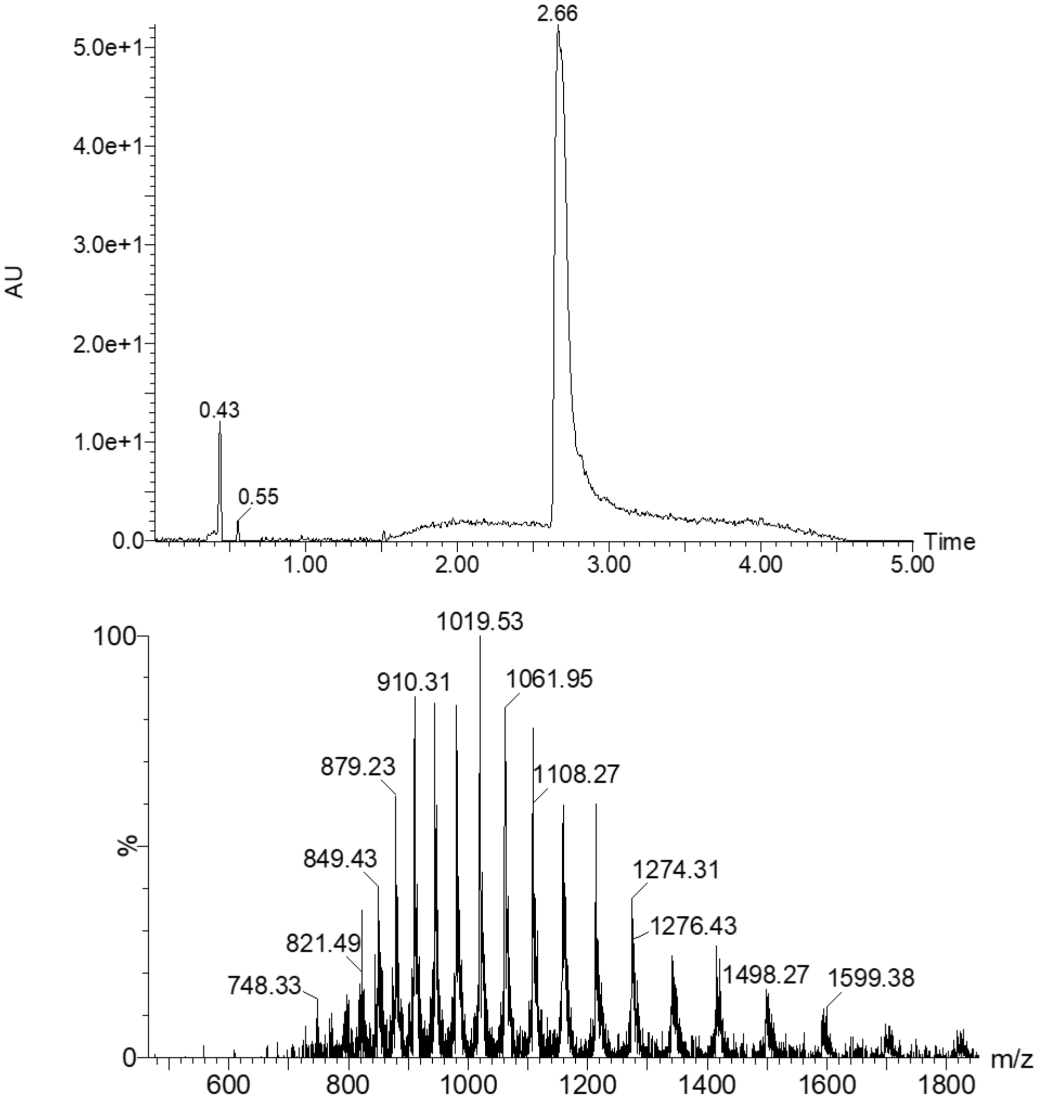

Figure S55. UV trace from analytical RP-UPLC and its ESI-MS of ***20c***. Gradient: 5-95% ACN/H_2O containing 0.1% TFA over 5 min at a flow rate of 0.4 mL/min. ESI-MS calculated for $C_{1108}H_{1739}N_{297}O_{374}S_8$ $[M+17H]^{17+}$ m/z = 1498.72, found 1498.27, $[M+18H]^{18+}$ m/z = 1415.52, found 1415.72, $[M+19H]^{19+}$ m/z = 1341.07, found 1341.46, $[M+20H]^{20+}$ m/z = 1274.07, found 1274.31, $[M+21H]^{21+}$ m/z = 1213.44, found 1213.94, $[M+22H]^{22+}$ m/z = 1158.33, found 1158.56, $[M+23H]^{23+}$ m/z = 1108.01, found 1108.27, $[M+24H]^{24+}$ m/z = 1061.89, found 1061.95, $[M+25H]^{25+}$ m/z = 1019.45, found 1019.53, $[M+26H]^{26+}$ m/z = 980.28, found 979.57, $[M+27H]^{27+}$ m/z = 944.01, found 944.43, $[M+28H]^{28+}$ m/z = 910.33, found 910.31, $[M+29H]^{29+}$ m/z = 878.98, found 879.23, $[M+30H]^{30+}$ m/z = 849.71, found 849.43.

4.5 Synthesis of *20e*

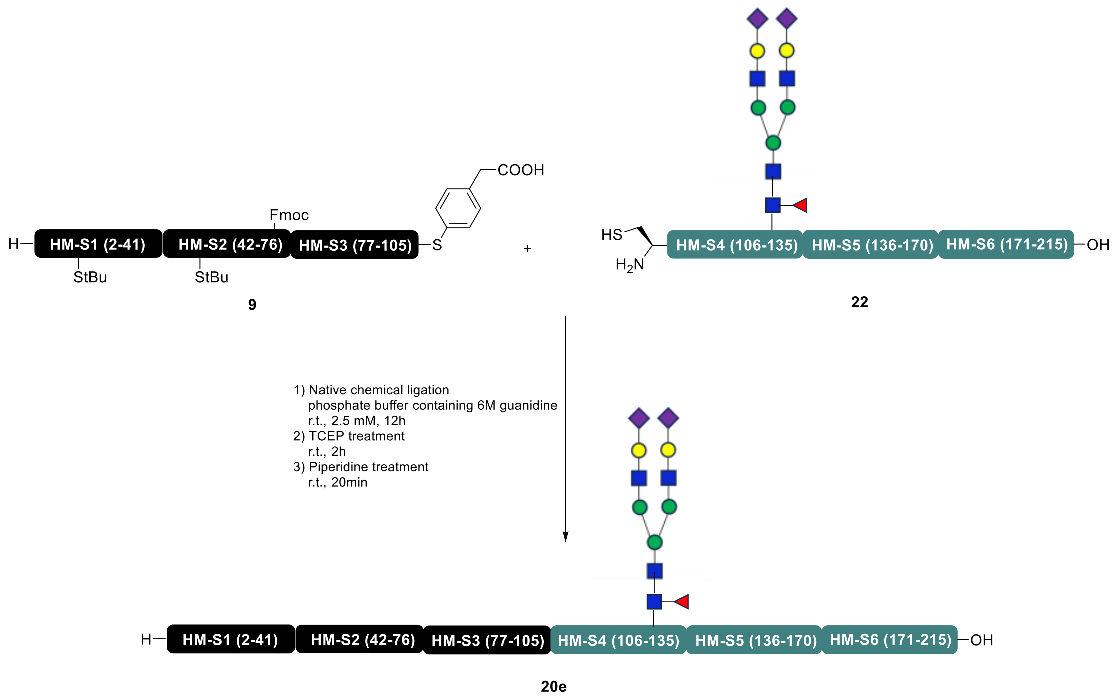

Figure S56. Synthetic route of ***20e.***

Peptidyl MPAA ester ***9*** (5.8 mg, 0.46 μmol) and peptidyl carboxylic acid ***22*** (5.6 mg, 0.37 μmol) dissolved in aqueous buffer containing 6 M Gn·HCl, 0.2 M NaH_2PO_4 and 100 mM MPAA (pH 7.0) at a concentration of 2.5 mM. The reaction was stirred at room temperature for 12h. Then, double volume of 0.1 M TCEP was added to the reaction solution for 2h to remove the S*t*Bu protection on cysteine and reduce the disulfide bond formed with MPAA during the reaction. After that, 20% piperidine (v/v) was added to the reaction solution for 20min to remove the Fmoc protection on lysine to afford ***20e***. The crude product was purified by preparative reverse-phase HPLC (10 to 60% ACN/H_2O over 45 min, 0.1% TFA). After lyophilization, 2.6 mg ***20e*** was obtained as a white solid in an isolated yield of 25.7% over 3 steps.

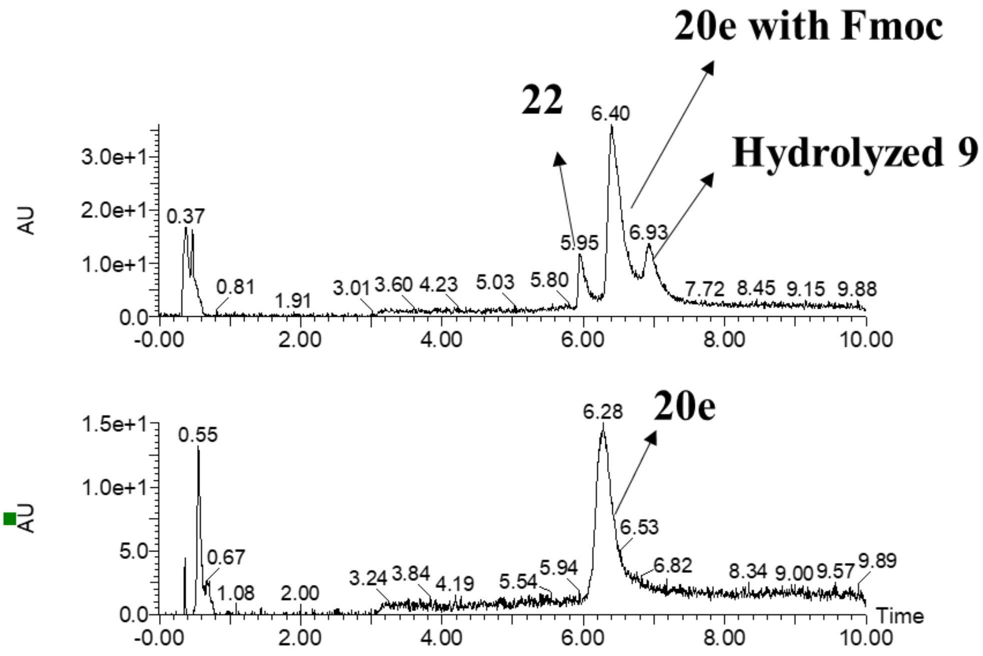

Figure S57. UV trace from analytical RP-UPLC of native chemical ligation reaction mixture between ***9*** and ***22***. **(A)** native chemical ligation at 12h after TCEP treatment. Gradient: 5-10-60% ACN/H_2O containing 0.1% TFA over 10 min at a flow rate of 0.4 mL/min. **(B)** After HPLC purification and lyophilization, piperidine treatment at 20min. Gradient: 5-10-60% ACN/H_2O containing 0.1% TFA over 10 min at a flow rate of 0.4 mL/min.

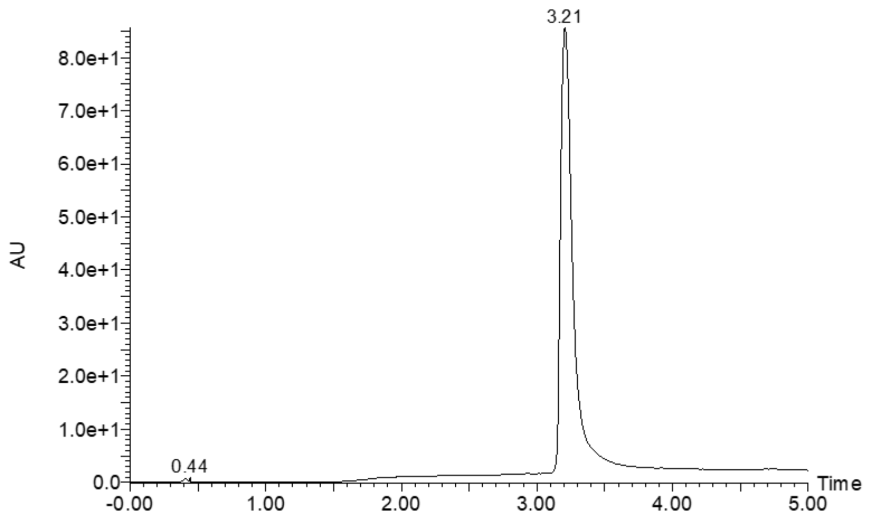

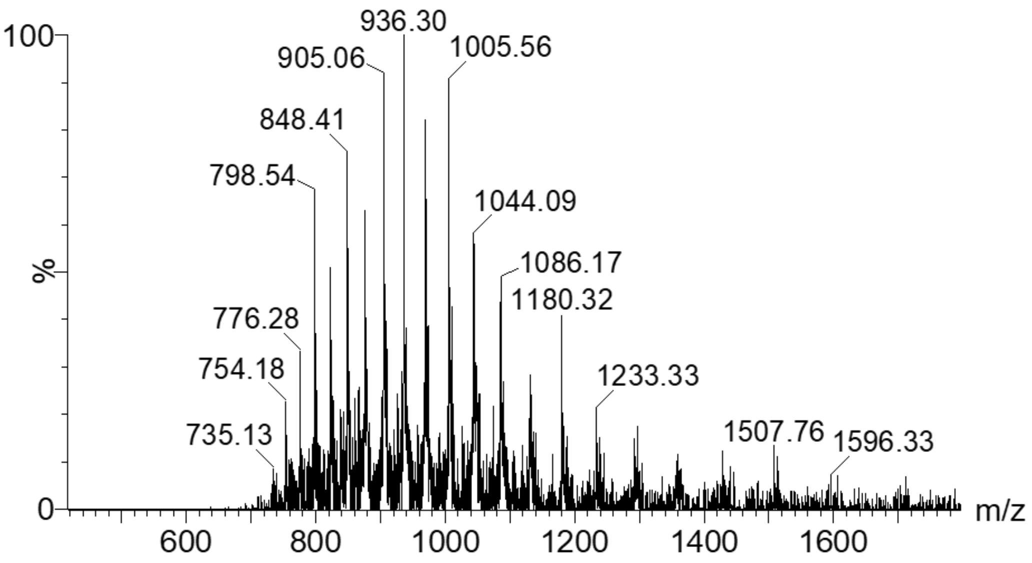

Figure S58. UV trace from analytical RP-UPLC and its ESI-MS of ***20e***. Gradient: 10-60% ACN/H_2O containing 0.1% TFA over 5 min at a flow rate of 0.4 mL/min. ESI-MS calculated for $C_{1170}H_{1839}N_{301}O_{421}S_8$ $[M+22H]^{22+}$ m/z = 1233.45, found 1233.33, $[M+23H]^{23+}$ m/z = 1179.90, found 1180.32, $[M+24H]^{24+}$ m/z = 1130.78, found 1131.13, $[M+25H]^{25+}$ m/z = 1085.59, found 1086.17, $[M+26H]^{26+}$ m/z = 1043.88, found 1044.09, $[M+27H]^{27+}$ m/z = 1005.25, found 1005.56, $[M+28H]^{28+}$ m/z = 969.38, found 969.58, $[M+29H]^{29+}$ m/z = 935.99, found 936.30, $[M+30H]^{30+}$ m/z = 848.33, found 848.41, $[M+31H]^{31+}$ m/z = 822.65, found 822.93, $[M+32H]^{32+}$ m/z = 798.49, found 798.54, $[M+33H]^{33+}$ m/z = 775.71, found 776.28, $[M+34H]^{34+}$ m/z = 754.19, found 754.18.

4.6 Synthesis of *20d*

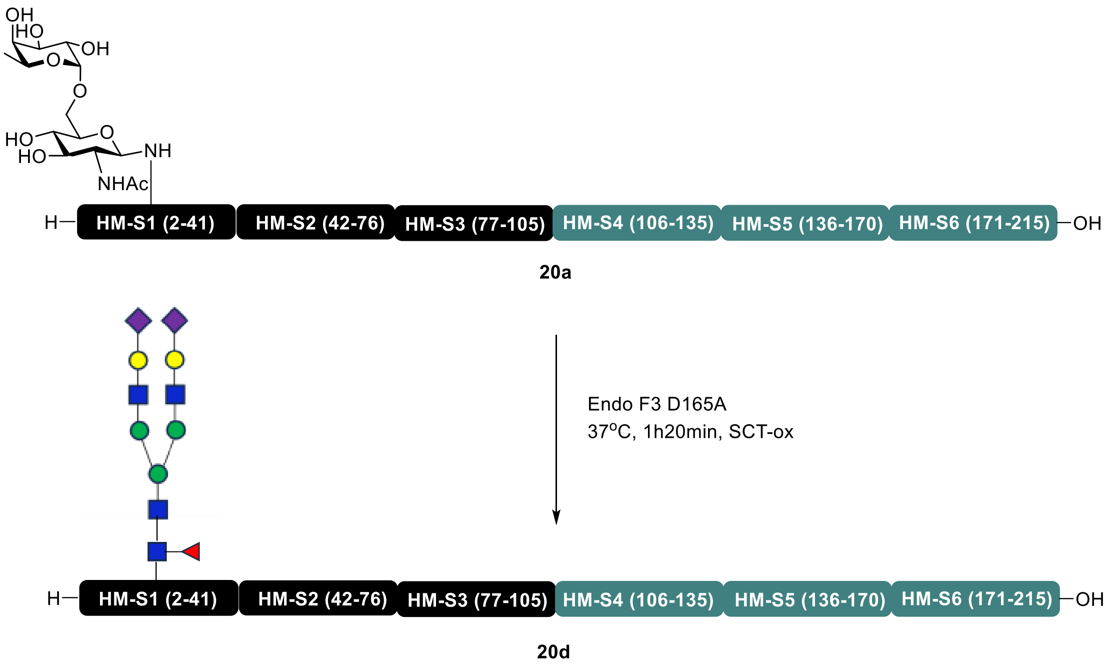

Figure S59. Synthetic route of ***20d.***

Peptide carboxylic acid ***20e*** (2.2 mg, 0.088 µmol) was dissolved in 10x PBS (pH 6.8). Then 86 µL Endo F3 D165A (5 µg/µL in 1x PBS) was added to the solution and mixed well. 50 equiv. SCTox was dissolved in 10x PBS and then added to the solution and mixed well. The mentioned solution was incubated at 37°C for 1h20min and monitored by UPLC-MS system. The crude product was purified by preparative reverse-phase HPLC (10 to 60% ACN/H_2O over 45 min, 0.1% TFA). After lyophilization, 1.0 mg ***20d*** was obtained as a white solid in an isolated yield of 42.1%.

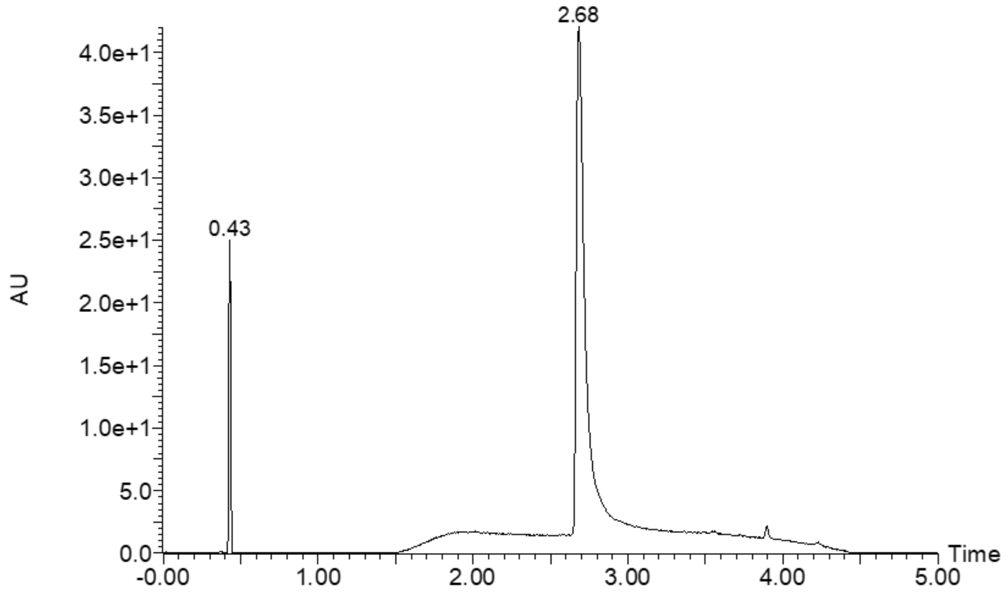

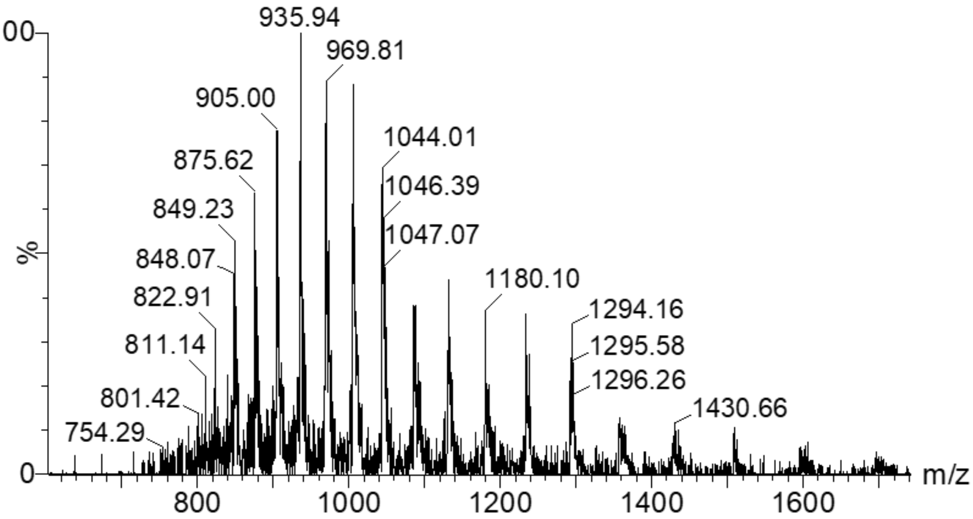

Figure S60. UV trace from analytical RP-UPLC and its ESI-MS of ***20d***. Gradient: 5-95% ACN/H_2O containing 0.1% TFA over 5 min at a flow rate of 0.4 mL/min. ESI-MS calculated for $C_{1170}H_{1839}N_{301}O_{421}S_8$ $[M+21H]^{21+}$ m/z = 1292.18, found 1294.16, $[M+22H]^{22+}$ m/z = 1233.45, found 1233.69, $[M+23H]^{23+}$ m/z = 1179.90, found 1180.10, $[M+24H]^{24+}$ m/z = 1130.78, found 1131.61, $[M+25H]^{25+}$ m/z = 1085.59, found 1085.84, $[M+26H]^{26+}$ m/z = 1043.88, found 1044.01, $[M+27H]^{27+}$ m/z = 1005.25, found 1005.58, $[M+28H]^{28+}$ m/z = 969.38, found 969.81, $[M+29H]^{29+}$ m/z = 935.99, found 935.94, $[M+30H]^{30+}$ m/z = 848.33, found 849.23, $[M+31H]^{31+}$ m/z = 822.65, found 822.91.

4.7 Synthesis of *20f*

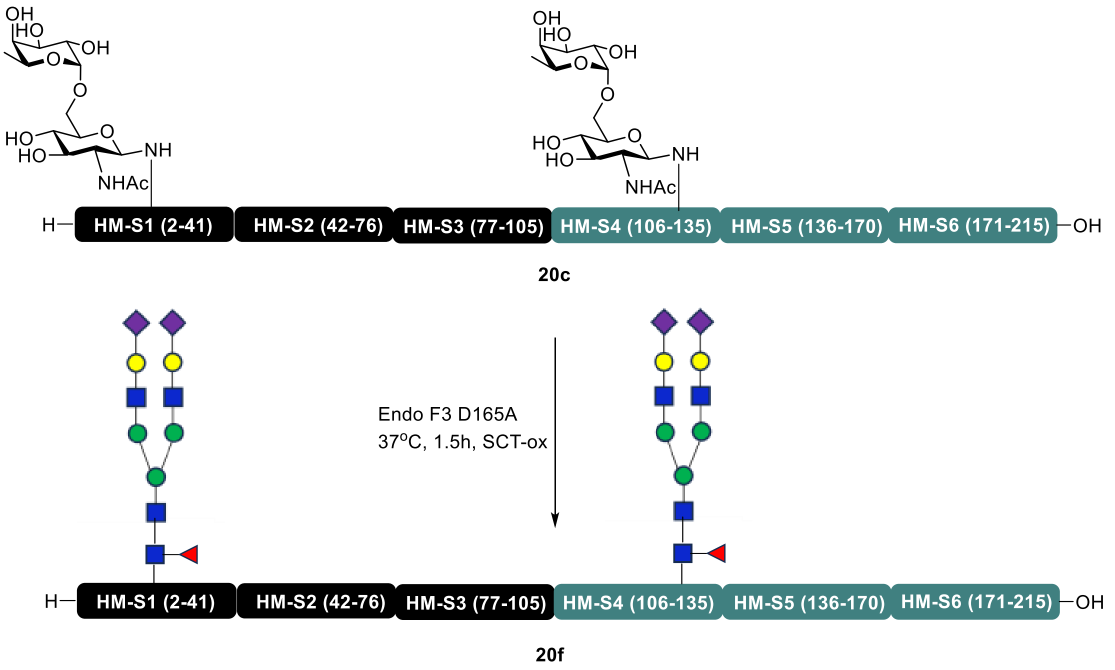

Figure S61. Synthetic route of ***20f.***

Peptide carboxylic acid ***20c*** (2.2 mg, 0.086 μmol) was dissolved in 10x PBS (pH 6.8). Then 200 μL Endo F3 D165A (5 μg/μL in 1x PBS) was added to the solution and mixed well. 200 equiv. SCTox was dissolved in 10x PBS and then added to the solution and mixed well. The mentioned solution was incubated at 37°C for 1.5h and monitored by UPLC-MS system. The crude product was purified by preparative reverse-phase HPLC (10 to 60% ACN/H_2O over 45 min, 0.1% TFA). After lyophilization, 1.3 mg ***20f*** was obtained as a white solid in an isolated yield of 51.0%.

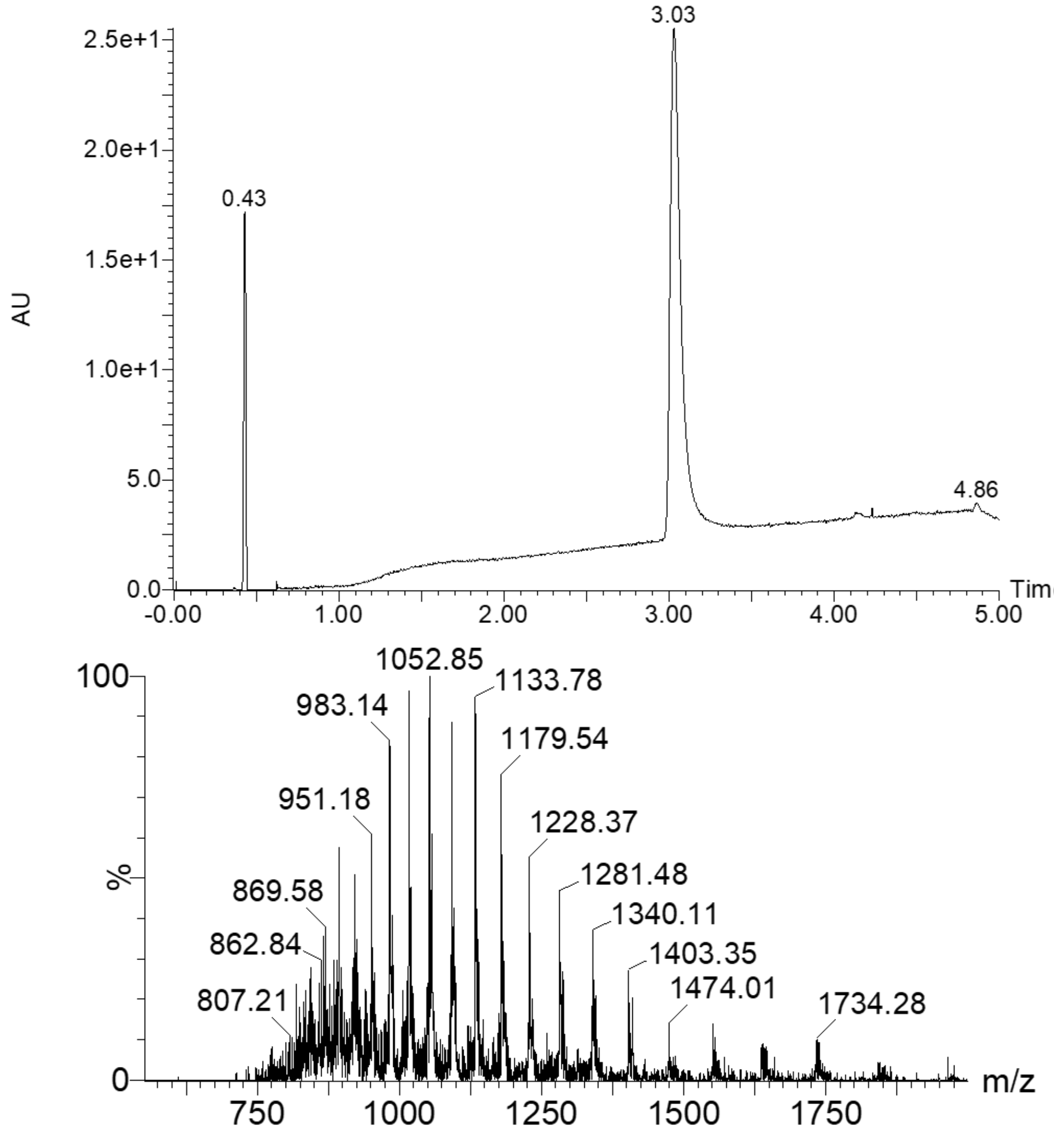

Figure S62. UV trace from analytical RP-UPLC and its ESI-MS of ***20f***. Gradient: 10-60% ACN/H_2O containing 0.1% TFA over 5 min at a flow rate of 0.4 mL/min. ESI-MS calculated for $C_{1260}H_{1985}N_{307}O_{486}S_8$ $[M+20H]^{20+}$ m/z = 1474.34, found 1474.01, $[M+21H]^{21+}$ m/z = 1404.19, found 1403.35, $[M+22H]^{22+}$ m/z = 1340.40, found 1340.11, $[M+23H]^{23+}$ m/z = 1281.48, found 1282.17, $[M+24H]^{24+}$ m/z = 1179.68, found 1179.54, $[M+25H]^{25+}$ m/z = 1134.34, found 1133.78, $[M+26H]^{26+}$ m/z = 1092.37, found 1091.68, $[M+27H]^{27+}$ m/z = 1053.39, found 1052.85, $[M+28H]^{28+}$ m/z = 1017.10, found 1016.81, $[M+29H]^{29+}$ m/z = 983.23, found 983.14, $[M+30H]^{30+}$ m/z = 951.54, found 951.18, $[M+31H]^{31+}$ m/z = 921.84, found 921.80.

5. Protein Folding and Circular Dichroism (CD) Measurement

The protein folding was done following previously reported protocols in our lab [4]. To further verify

the redox status was not changed, we did a control experiment. Briefly, E. coli HMGB1 was treated with TCEP in phosphate buffer containing 6 M Gn·HCl and then desalted using preparative HPLC. After lyophilization, completely reduced E. coli HM was firstly subjected to HR-ESI-MS analysis to confirm its reduced state. (**Figure S63**) Then the reduced HMGB1 was diluted in refolding buffer (0.5mg/mL), which is consisted of 50 mM Tris, 500 mM NaCl under pH 7.5. The refolding process was incubated for 24 hours at 4 ^{o}C. Subsequently, the protein buffer was desalted and analyzed with high resolution ESI-MS again. (**Figure S64**) The nearly same detected high-resolution mass after deconvolution was consistent with the synthetic unmodified HMGB1, which proves the refolding process would not introduce disulfide bond within HMGB1 molecule. The folded chemical synthesized HMGB1 and its glycosylated variants and E. coli expressed HMGB1 were dissolved and diluted in PBS buffer (pH 7.4). The final concentrations of those proteins and the recombinant one were around 0.1-0.3 mg/mL. (The protein concentration was determined by Thermo Scientific NanoDrop UV-Vis spectrophotometers) The CD spectra were measured by a J-815 circular dichroism spectrometer (JASCO), each sample was scanned in 197-260 nm for 1 time at room temperature using 1x PBS buffer (pH 7.4) as blank in a 0.1 cm cell. The CD spectra of E. coli-HM and three N-glycosylated HMGB1 variants along with temperature change were shown in **Figure S65**. At 65 ^{o}C (red curves in **Figure S65**), E. coli-HM almost lost the typical α-helical structure while glycosylated HMGB1 still presented apparent structures though the signal gradually decreased following temperature increase. This result indicates the *N*-glycosylation helps maintain the secondary structure of HMGB1 against degradation with temperature increase.

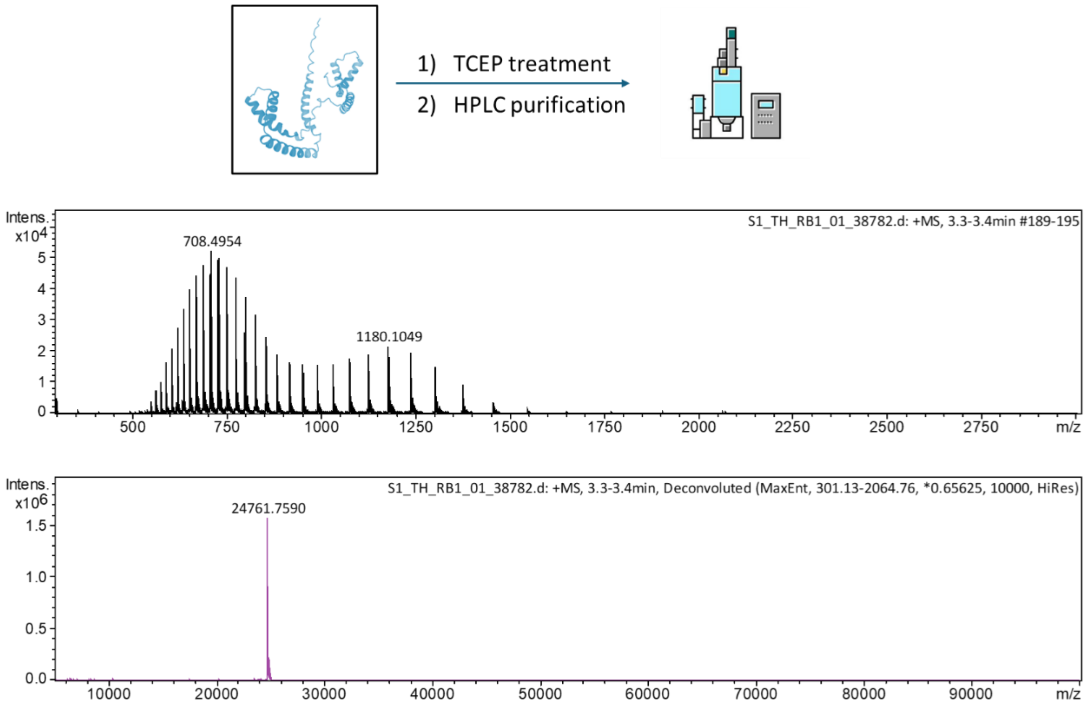

Figure S63. High resolution ESI-MS analysis of reduced *E. coli* expressed HMGB1.

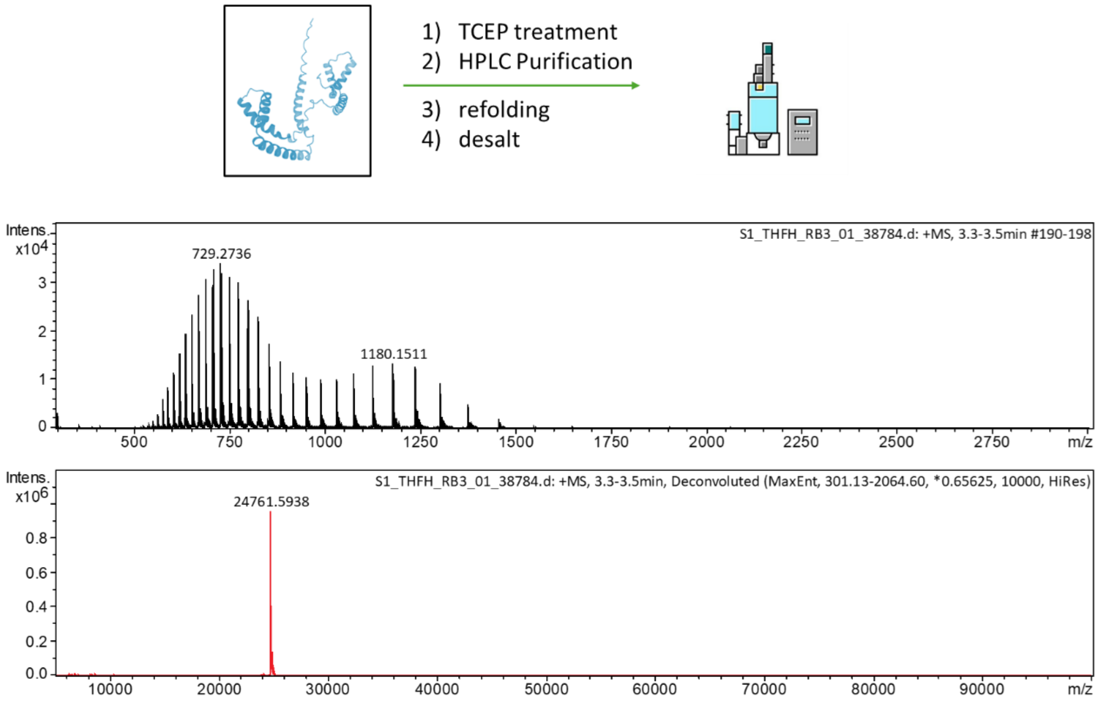

Figure S64. High resolution ESI-MS analysis of refolded reduced HMGB1.

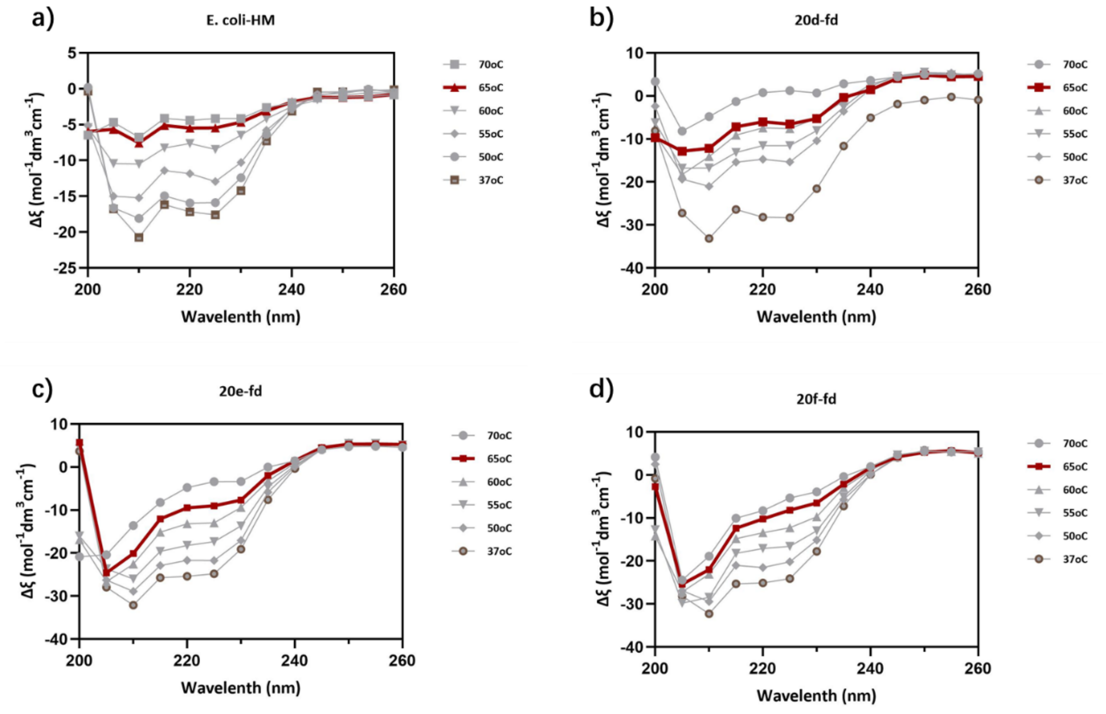

Figure S65. CD spectra of **E. coli-HM**, **20d-fd**, **20e-fd** and **20f-fd** at different temperatures.

III. Microscale Thermophoresis (MST) Binding Affinity Assay

To serve as targets, HEK 293T cell derived hRAGE (MedChemExpress) and NS0 cell-derived hTLR-4 (R&D systems) were labelled using the Alexa Fluor 647 NHS ester dye (Thermo Fisher Scientific) according to the manufacturer's instructions and diluted in 1x PBS supplemented with 0.1% Tween 20 to appropriate concentrations for detection. While the synthetic and E. coli expressed HMGB1 proteins as ligands were maintained in 1x PBS. The labelled targets and unlabelled ligands were mixed in a 1:1 volume ratio and incubated for 10 min at room temperature in the dark. MST was measured using a Monolith X instrument (NanoTemper Technologies) at an ambient temperature 25 ^{o}C. Instrument parameters were adjusted to autodetected suitable LED power and medium MST power. Data of three to four independently measurements were also analyzed Monolith X using the signal from an MST (670nm)-on time of 1.5 s.

IV. Cell Culture and Enzyme-Linked Immunosorbent Assay (ELISA)

NIH/3T3 cells were purchased from Cell Bank/Stem Cell Bank, Chinese Academy of Sciences and cultured in DMEM (Gibco) medium supplemented with 10% New Born Calf Serum (NCBS) (Gibco) ,1% penicillin-streptomycin (Gibco) at 37 ^{o}C with 5% CO_2. 5x10^4 NIH/3T3 cells were seeded in each well of 96-well plate, followed by 12 hours incubation stimulated with or without

HMGB1 proteins at a concentration of 180 nM. The supernatants were collected and subjected to sandwich ELISA using a purchased CXCL12 ELISA kit (R&D systems) following the manufacture's protocols. The O.D. values at 450 nm were measured using a microplate reader (BioTeK Instruments, INC).

V. Cell Migration Assay

NIH/3T3 cells were seeded in 24-well transwell inserts (CoStar, 8μm pore size, polycarbonate) with 7.5 x 10^4 cells per insert with serum free medium. The lower chambers were placed with normal culture medium with or without HMGB1 proteins. The cells were incubated under normal conditions for 9 hours. Then, inserts were removed and washed twice with 1x PBS (Gibco), fixed with 4% paraformaldehyde (Sigma Aldrich) and stained with 0.1% crystal violet solution (Sigma Aldrich). The staining in the cells was eluted out by 33% acetic acid (Sigma Aldrich) and O.D. values at 590 nm were measured using a microplate reader (BioTeK Instruments, INC). The UV absorbance at 590 nm was positively correlated with the cell numbers.

VI. Molecular Dynamics Simulations

The initial structures of the RAGE-HMGB1 complexes were predicted using SeedFold. [5] Four systems were constructed, corresponding to RAGE in complex with 12-fd (non-glycosylated HMGB1), 20d-fd (HMGB1 glycosylated at Asn37), 20e-fd (HMGB1 glycosylated at Asn134), and 20f-fd (HMGB1 glycosylated at both Asn37 and Asn134). The predicted complex structures were subsequently processed using CHARMM-GUI [6,7] for system building, solvation, and generation of simulation input files.

For the MD simulations, the protein components were described using the AMBER ff19SB force field [8], and the systems were solvated with the OPC water model [9]. For the glycosylated systems, AMBER-compatible glycan parameters were assigned during the CHARMM-GUI preparation workflow. All simulations were carried out using GROMACS [10].

Each system was first subjected to energy minimization using the steepest descent algorithm for 5000 steps, with an energy minimization tolerance of 1000.0 kJ mol^{-1} nm^{-1}. During this stage, positional restraints were applied to the solute using the settings generated in the CHARMM-GUI protocol, with backbone and side-chain restraint force constants of 400.0 and 40.0 kJ mol^{-1} nm^{-2}, respectively, together with dihedral restraints of 4.0. A Verlet cutoff scheme was used. Short-range van der Waals and Coulomb interactions were truncated at 0.9 nm, while long-range electrostatic interactions were treated using the particle mesh Ewald (PME) method [11]. All bonds involving hydrogen atoms were constrained using the LINCS algorithm [12].

Following minimization, the systems were equilibrated in the NVT ensemble for 125 ps with a 1 fs

integration time step. The temperature was maintained at 300 K using the velocity-rescaling thermostat [13], with separate coupling groups for solute and solvent. The same positional and dihedral restraints were retained during equilibration. Initial velocities were generated at 300 K.

Production simulations were then performed in the NPT ensemble using a 2 fs time step. The temperature was maintained at 300 K with the velocity-rescaling thermostat [13], and the pressure was kept at 1.0 bar under isotropic coupling using the C-rescale barostat [14]. The cutoff scheme remained identical to that used during minimization and equilibration, with 0.9 nm cutoffs for the neighbor list, van der Waals interactions, and short-range electrostatic interactions, and PME treatment for long-range electrostatics. Dispersion correction was applied to both energy and pressure. A total of 10^8 steps were performed (=200 ns).

For the analyses shown in this work, RMSD was monitored over 0-200 ns, whereas RMSF, hydrogen-bond analysis, and B-factor analysis were conducted using the 100-200 ns interval as the equilibrated segment. The B-factor-mapped HMGB1 structures shown in the figure correspond to time-averaged coordinates over 100-200 ns rather than a single snapshot. For clarity, RAGE was omitted from the B-factor structural display, although the analyzed conformations were all taken from the RAGE-HMGB1 complexes.

VII. Binding Free Energy Calculations Using gmx_MMPBSA

Binding free energies for the RAGE-HMGB1 complexes were estimated using the gmx_MMPBSA package [15] based on the single-trajectory MM/PBSA approach. Snapshots were extracted from the equilibrated portion of the MD trajectories. According to the input settings used in this study, frames from 1001 to 2001 (100 ns to 200 ns) were included in the default calculation, with an interval of 1 frame, corresponding to the analyzed segment of the production trajectory.

The PB calculations were performed at 300 K with an ionic strength of 0.150 M. The solvent dielectric constant was set to 80.0, and the solute dielectric constant (indi) was set to 4.0 in the main calculations. Other parameters were set as follows: PBRadii = 3, fillratio = 4.0, radiopt = 0, inp = 2, scale = 2.0, linit = 1000, and probe radius = 1.4 Å. The nonpolar contribution was estimated using a solvent-accessible surface area model with cavity_surften = 0.0378 and cavity_offset = -0.5692.

Because the systems studied here involve glycosylated proteins, indi = 4.0 was used as the primary setting for MM/PBSA analysis. For comparison, additional calculations were also carried out with alternative indi values and different frame numbers, as shown in Figure X. The reported binding free energies are presented as mean ± standard deviation over the sampled frames.

VIII. Reference

1. Wu, H.; Sun, Z.; Li, X. *N,O*-Benzylidene Acetal Dipeptides (NBDs) Enable the Synthesis of Difficult Peptides via a Kinked Backbone Strategy. *Angewandte Chemie International Edition* **2023,** *62* (44), e202310624.

2. Huang, W.; Li, J.; Wang, L.-X. Unusual Transglycosylation Activity of Flavobacterium meningosepticum Endoglycosidases Enables Convergent Chemoenzymatic Synthesis of Core Fucosylated Complex N-Glycopeptides. *ChemBioChem* **2011,** *12* (6), 932-941.

3. Lee, C. L.; Liu, H.; Wong, C. T. T.; Chow, H. Y.; Li, X. Enabling N-to-C Ser/Thr Ligation for Convergent Protein Synthesis via Combining Chemical Ligation Approaches. *Journal of the American Chemical Society* **2016,** *138* (33), 10477-10484.

4. Wei, T.; Liu, J.; Li, C.; Tan, Y.; Wei, R.; Wang, J.; Wu, H.; Li, Q.; Liu, H.; Tang, Y.; Li, X. Revealing the extracellular function of HMGB1 N-terminal region acetylation assisted by a protein semi-synthesis approach. *Chemical Science* **2023,** *14* (37), 10297-10307.

5. Zhou, Y.; Lu, C.; Ma, Y.; Qu, W.; Ye, F.; Zhang, K.; Wang, L.; Gui, M.; Gu, Q. SeedFold: Scaling Biomolecular Structure Prediction. arXiv:2512.24354, 2025.

6. Jo, S.; Kim, T.; Iyer, V. G.; Im, W. CHARMM-GUI: A Web-Based Graphical User Interface for CHARMM. *J. Comput. Chem.* **2008**, *29*, 1859-1865.

7. Lee, J.; Cheng, X.; Swails, J. M.; Yeom, M. S.; Eastman, P. K.; Lemkul, J. A.; Wei, S.; Buckner, J.; Jeong, J. C.; Qi, Y.; Jo, S.; Pande, V. S.; Case, D. A.; Brooks, C. L.; MacKerell, A. D.; Klauda, J. B.; Im, W. CHARMM-GUI Input Generator for NAMD, GROMACS, AMBER, OpenMM, and CHARMM/OpenMM Simulations Using the CHARMM36 Additive Force Field. *J. Chem. Theory Comput.* **2016**, *12*, 405-413.

8. Tian, C.; Kasavajhala, K.; Belfon, K. A. A.; Raguette, L.; Huang, H.; Migues, A. N.; Bickel, J.; Wang, Y.; Pincay, J.; Wu, Q.; Simmerling, C. ff19SB: Amino-Acid-Specific Protein Backbone Parameters Trained against Quantum Mechanics Energy Surfaces in Solution. *J. Chem. Theory Comput.* **2020**, *16*, 528-552.

9. Izadi, S.; Anandakrishnan, R.; Onufriev, A. V. Building Water Models: A Different Approach. *J. Phys. Chem. Lett.* **2014**, *5*, 3863-3871.

10. Abraham, M. J.; Murtola, T.; Schulz, R.; Pall, S.; Smith, J. C.; Hess, B.; Lindahl, E. GROMACS: High Performance Molecular Simulations through Multi-Level Parallelism from Laptops to Supercomputers. *SoftwareX* **2015**, *1-2*, 19-25.

11. Darden, T.; York, D.; Pedersen, L. Particle Mesh Ewald: An N.log(N) Method for Ewald

Sums in Large Systems. *J. Chem. Phys.* **1993**, *98*, 10089-10092.

12. Hess, B.; Bekker, H.; Berendsen, H. J. C.; Fraaije, J. G. E. M. LINCS: A Linear Constraint Solver for Molecular Simulations. *J. Comput. Chem.* **1997**, *18*, 1463-1472.

13. Bussi, G.; Donadio, D.; Parrinello, M. Canonical Sampling through Velocity Rescaling. *J. Chem. Phys.* **2007**, *126*, 014101.

14. Bernetti, M.; Bussi, G. Pressure Control Using Stochastic Cell Rescaling. *J. Chem. Phys.* **2020**, *153*, 114107.

15. Valdes-Tresanco, M. S.; Valdes-Tresanco, M. E.; Valiente, P. A.; Moreno, E. gmx_MMPBSA: A New Tool to Perform End-State Free Energy Calculations with GROMACS. *J. Chem. Theory Comput.* **2021**, *17*, 6281-6291.
